# A Polyomavirus-Positive Merkel Cell Carcinoma Mouse Model Supports a Unified Origin for Somatic and Germ Cell Cancers

**DOI:** 10.3390/cancers17172800

**Published:** 2025-08-27

**Authors:** Wendy Yang, Sara Contente, Sarah Rahman

**Affiliations:** Department of Pathology, Uniformed Services University of Health Sciences, 4301 Jones Bridge Road, Bethesda, MD 20814, USA; sara.contente@usuhs.edu (S.C.); sarah.rahman.ctr@usuhs.edu (S.R.)

**Keywords:** malignant somatic transformation, primordial germ cell, primordial germ cell like cell, virus-positive Merkel cell carcinoma, small cell neuroendocrine carcinoma, global DNA hypomethylation, Weismann Barrier, induced pluripotent stem cell, embryonic germ cell, embryonic germ cell like cell

## Abstract

Background/Objectives: The Germ Cell Theory of cancer posits that human primordial germ cells (hPGCs) are the cells of origin for malignancies. While this theory is well established for germ cell cancers, a germ cell origin for somatic cancers has been largely overlooked despite clinical observations of malignant somatic transformation (MST), wherein germ cell cancers give rise to diverse somatic cancer phenotypes, often without additional mutations. Methods: To test the Germ Cell Theory experimentally in somatic cancer, we established a virus-driven MST model linking hPGC-like cells (hPGCLCs) to Merkel cell polyomavirus (MCPyV)-positive Merkel cell carcinoma (MCC), a highly aggressive somatic cancer with a germ cell cancer-like, low-mutation epigenetic profile. The MCPyV genome was transduced into human induced pluripotent stem cells (hiPSCs) or hPGC-like cells by lentiviral transfection, followed by xenotransplantation. Results: Virus-positive MCC (VP-MCC)-like tumors were consistently induced without additional oncogenic mutations. These tumors recapitulated VP-MCC’s high-grade neuroendocrine carcinoma histology and molecular profiles. DNA methylation analysis revealed near-complete global hypomethylation in VP-MCC-like tumors, matching the unique epigenetic state of late-stage hPGCs. Notably, pluripotent intermediates were neither necessary nor sufficient for MST; transformation required acquisition of a late-hPGC-like epigenetic state. Conclusions: This is the first MST model of a somatic cancer arising through an aberrant germline-to-soma transition. Our findings unify VP-MCC and germ cell cancer biology, challenge mutation- and soma-centric paradigms, and provide a tractable platform to investigate developmental and epigenetic mechanisms of oncogenesis. This MST model supports a unifying germ cell origin for both germ cell and non-germ cell somatic malignancies.

## Simple Summary

Cancer research has long focused on mutations in normal body cells, but this approach has not produced major breakthroughs for most cancers. Our study explores a different concept that some aggressive cancers may actually arise from early reproductive cells called primordial germ cells, which normally develop into eggs and sperm. We created a new experimental model showing how a virus can transform human primordial germ cell-like cells into virus-positive Merkel cell carcinoma, a rare but deadly skin cancer. This model shows that cancers can emerge through changes in developmental states rather than relying solely on genetic mutations. By linking cancer development to early germ cells, our findings suggest a unifying explanation for both germ cell cancers and body cancers. This new perspective may guide more effective approaches to study, diagnose, and treat cancer by focusing on early human development rather than only DNA mutations and later developmental stages.

## 1. Introduction

Cancer has traditionally been classified into two primary groups based on presumed cellular origin: germ cell cancers, arising from reproductive germline cells which constitute less than 1% of malignancies [1], and somatic cancers, originating from non-germline somatic cells, representing the vast majority of malignancies. Despite this traditional separation, all cancers share phenotypic hallmarks, many of which recapitulate early embryonic/germ cell development, suggesting developmental arrest or “blocked ontogeny” [2,3]. This principle, encapsulated as “oncology recapitulates ontogeny,” has significantly influenced germ cell cancer research and clinical practice yet remains largely overlooked in somatic cancer studies [4,5] despite its clinical relevance to grading and prognosis [6].

The Germ Cell Theory, grounded in developmental biology, has yielded transformative insights into germ cell tumor biology and curative treatment strategies [1,4]. Germ cell cancers are genetically simple and epigenetically distinct [1,4]. Germ cell cancers originate from human primordial germ cells (hPGCs) [1,7] that are founder cells of the germline specified during weeks 2–3 of development [8]. hPGCs exhibit latent pluripotency and intrinsic cancer stem cell-like properties, predisposing them to malignant transformation with minimal genetic alteration [1]. In germ cell cancers, transformed unipotent late-hPGCs and totipotent embryonal carcinoma cells constitute the cancer stem cell compartments of seminomas or non-seminomatous germ cell cancers, respectively. Embryonic carcinoma cells are believed to be the in vivo totipotent parthenogenic derivatives of transformed late-hPGCs [1]. Seminomas are composed entirely of transformed hPGCs, while non-seminomatous germ cell cancers such as embryonic carcinomas or malignant teratomas consist solely of embryonic carcinoma cells or arise through differentiation into somatic lineages across all three germ layers from embryonic carcinoma cells, respectively [1].

Mounting biological and clinical evidence increasingly challenges the traditional separation between germ cell cancers and somatic cancers, suggesting a shared developmental origin. Notably, approximately 3–6% of germ cell tumors undergo malignant somatic transformation (MST) into somatic cancer phenotypes, often without additional mutations [4,9,10,11]. GCTs can also manifest as extragonadal GCTs, demonstrating the capacity to arise at virtually any anatomical site, albeit with a predilection for midline locations [12]. Many somatic cancers aberrantly express cancer testis antigens normally restricted to germ cells [13,14], and acquisition of a primordial germ cell (PGC)-like state has been shown to be essential for metastasis [15]. Furthermore, global DNA hypomethylation is a ubiquitous epigenetic hallmark of all cancers, observed in both GCCs and somatic cancers [1,16]. Despite these converging features, somatic cancer research remains dominated by the fragmented, mutation-centric Somatic Mutation Theory, which lacks a unifying biological framework and has yielded limited improvements in patient outcomes [5]. Although the cancer stem cell theory has more recently gained recognition in somatic cancer research, it fundamentally differs from the germ cell theory in its soma-centric view by positing somatic adult stem cells as the origin of somatic cancers [17]. However, the precise identity of these somatic adult stem cells remains undefined. Given the substantial biological and clinical parallels between germ cell cancers and somatic cancers, it is reasonable to propose that hPGCs (or a PGC-like state) may represent a shared stem cell origin for both, at least in some cases. However, no experimental model has yet directly linked hPGCs to somatic cancer via MST [18], despite the longstanding use of teratoma assays to study GCT development and tumorigenesis [19]. These assays, which generate teratomas (a form of non-seminomatous germ cell tumor) in immunocompromised mice following injection of human pluripotent stem cells, remain the gold standard for confirming pluripotency due to the intrinsic coupling of pluripotency and teratoma formation [20]. While MST most commonly arises in clinical teratomas, it has not been recapitulated in these models. This is likely due to the limited duration of in vivo assays relative to the prolonged latency observed in human disease [18].

Virus-positive Merkel cell carcinoma (VP-MCC), an aggressive high grade neuroendocrine carcinoma of the skin, comprises ~80% of MCC cases and is defined by the presence of randomly but clonally integrated, C-terminus truncated Merkel cell polyomavirus (MCPyV) genomes (~5 kb) [21,22,23]. It presents two key features that seem to make it ideally suited for developing an MST model. First, VP-MCC exhibits extreme genomic stability, in stark contrast to virus-negative MCC (VN-MCC), which comprises the remaining ~20% and is among the most highly mutated human cancers. Similarly, other virus-associated cancers, such as HPV- and EBV-positive tumors, typically show higher mutational burdens than their virus-negative counterparts [24]. This exceptional genomic stability mirrors that of germ cell tumors and would enable the development of a genetically simple, experimentally tractable MST model. Although MCPyV is ubiquitous in normal skin flora, VP-MCC is very rare, and MCPyV transduction of various cutaneous lineage cells have failed to reproduce its molecular and histologic features. This underscores the paramount importance of cellular context in VP-MCC carcinogenesis, a principle is also similar to germ cell cancer biology [22,23,25]. Second, the small cell neuroendocrine carcinoma phenotype of MCC represents a poorly differentiated, aggressive end-stage state to which diverse somatic cancers converge upon progression [26]. Small cell neuroendocrine carcinomas, including MCC, also exhibit stem-like properties and multilineage differentiation potential [27,28], suggesting that VP-MCC may be composed predominantly of somatic cancer stem cells, functionally analogous to seminomas or embryonal carcinomas in germ cell cancers. Together, these features support the potential of developing a VP-MCC-based MST model capable of capturing transformation events rapidly with immediate somatic differentiation arrests, within the temporal limits of mouse xenograft assays, where MST has otherwise not been observed [18]. To directly test the Germ Cell Theory in somatic cancer, we proceeded to establish a mouse model based on the hypothesis that VP-MCC arises through MCPyV-driven MST of extragonadal hPGCs residing in the adult cutaneous niche. Multiple lines of evidence support our hypothesis. MCPyV tropism toward hPGCs is indicated by the relatively high level of MCPyV DNA detected in seminoma [29], a germ cell cancer composed of transformed hPGCs [1]. Inactivating mutations of p53 have been associated with MST [1] while MCPyV is known to indirectly inhibit p53 [23]. The skin microenvironment is enriched in stem cell factor (SCF) and stromal cell-derived factor 1 (SDF-1), key regulators of hPGC survival and chemotaxis [30,31]. Furthermore, the male predominance and head and neck predilection of MCC reflect Y chromosome-mediated hPGC viability and their characteristic midline migratory trajectory [1,12].

To investigate the proposed MCPyV-driven VP-MCC tumorigenic pathway, we designed a teratoma assay-like experiment aiming to prove MST from MCPyV transfected hPGCs to VP-MCC-like tumors in the adult mouse cutaneous niche. We transduced a truncated MCPyV genome (L82) using a lentiviral vector into in vitro surrogates of hPGCs and two relevant human pluripotent stem cell types: human embryonic germ cells (hEGCs) and human embryonic stem cells (hESCs), namely hPGC-like cells (hPGCLCs), hEGC-like cells (hEGCLCs), and hiPSCs. Germ cells, especially hPGCs, are known to possess latent or poised totipotent/pluripotent potential [32]. To distinguish them from adult human stem cells, which have limited unipotent or multipotent developmental capacity, we refer to hPGCs and hPGCLCs, together with human pluripotent stem cells such as hESCs, hiPSCs, hEGCs, and hEGCLCs, as “primeval stem cells” [4]. We use this term in our study to conceptually group stem cells with either latent or explicit totipotent/pluripotent potential, in contrast to adult stem cells with restricted developmental potency.

To trace tumorigenic pathways from primeval stem cells, we used global DNA methylation as an epigenetic marker to distinguish different cell states. Although global DNA methylation is dynamic during development, it can serve as a relatively stable marker that preserves lineage memory. Cancer cells may maintain DNA methylation signatures reflective of their cell of origin despite reprogramming. In normal human development, only two waves of global DNA demethylation occur, each producing a transient hypomethylation nadir during early embryogenesis. The first bottom occurs in totipotent naïve hESC/inner cell mass stage (~40% median global DNA methylation); the second and the lowest bottom occurs in the late hPGC stage (~6% median global DNA methylation) [33,34] (Figure S1). The second bottom’s extremely low global DNA methylation with near complete global DNA methylation erasure is unique to late hPGCs, while the first nadir’s moderate DNA hypomethylation characterizes the totipotent naïve hESC/inner cell mass state.

## 2. Materials and Methods

### 2.1. Cell Culture

The human iPSCA4 line (hiPSC_A4) generated from commercially obtained Caucasian male (46XY) neonatal foreskin fibroblasts and the derived GFP+ long-term culture human primordial germ cell like cell (LTC-hPGCLC) A4 line (hPGCLC_A4) were gifted by the Shioda lab [35]. The human embryonic germ cell like cell (hEGCLC) A4 line (hEGCLC_A4) was derived from hPGCLC_A4 based on a 10-day conversion protocol facilitated by SCF and FGF2 from the Shioda lab [35]. Both hiPSC and hEGCLC cultures were maintained in Essential 8 medium (Gibco, Brooklyn, NY, USA, Cat#A1517001) or occasionally Essential 8 Flex medium (Gibco, Brooklyn, NY, USA, Cat#A2858501) on human ESC-qualified Matrigel (Corning, New York, NY, USA, Cat#354277) and passaged at every 3–7 days using ReLeSR (StemCell Technologies, Vancouver, BC, Canada, Cat#100-0483) in the presence of the ROCK inhibitor Y-27632 (StemCell Technologies, Vancouver, BC, Canada, Cat#72304). Karyotyping analyses were performed every 10–15 passages to assure the genetic integrity of hiPSCs, hEGCLCs, and hPGCLCs and their lentivirus transfected counterparts (Figure S2). The six VP-MCC cell lines CVG-1 (RRID:CVCL_VL95), MKL-1 (RRID:CVCL_2600), MKL-2 (RRID:CVCL_D027), MS-1 (RRID:CVCL_E995), PeTa (RRID:CVCL_LC73), and WaGa (RRID:CVCL_E998) were obtained from Merkel cell carcinoma research labs (Yuan Chang and Patrick Moore Lab, Isaac Brownell Lab and James DeCaprio Lab) [36,37,38,39,40,41,42], cultured in RPMI (Roswell Park Medical Institute, Buffalo, NY, USA) 1640 medium (Gibco, Brooklyn, NY, USA, Cat#11875093) with 10% FBS (Sigma, Burlington, VT, USA, Cat#F2442-500ML) as suspension cultures, and passaged every 3–4 days with a 1:2 to 1:10 split. The two variant VN-MCC cell lines MCC-13 (RRID:CVCL_2583) and UISO (RRID:CVCL_E996) obtained from Yuan Chang and Patrick Moore Lab [41,43,44] and the TCam2 cell line (RRID:CVCL_T012) obtained from Alan Epstein lab [45] were also cultured in RPMI 1640 medium (Gibco, Brooklyn, NY, USA, Cat#11875093) with 10% FBS (Sigma, Burlington, NY, USA, Cat#F2442-500ML) as adherent cultures and passaged every 3–4 days with incubation in 0.25% trypsin (Gibco, Brooklyn, NY, USA, Cat#15090046) for up to 8 min. Mycoplasma contamination testing was performed every six months or when there was a suspicion for contamination, which was ruled out by performing a PCR based mycoplasma detection kit based on manufacturer’s protocol (PCR mycoplasma detection kit, Thermo Scientific, Waltham, MA, USA, Cat#J66117.AMJ).

### 2.2. Derivation of MCPyv+ Primeval Stem Cell Lines hPGCLC_A4_L82, hiPSC_A4_L82, and hEGCLC_A4_L82

The lentivirus virus vector plenti.puro.MCV.ER.RAZ.2 (a gift from Reety Arora and Sudhir Krishna Addgene plasmid #114382; http://n2t.net/addgene:114382, accessed on 6 February 2024; RRID:Addgene_114382) abbreviated as “L82” containing a C-terminus truncated MCPyV genome and puromycin resistance gene, a second generation packaging vector psPAX2 (AddGene#12260), and an envelope vector pMD2.G (AddGene#12259) were purchased from AddGene.Org. Lentivirus productions of MCPyV containing lentivirus were performed with FuGene 6 transfection reagent (Promega, Madison, WI, USA, Cat#E2691) and OptiMem serum-free medium (Invitrogen, Carlsbad, CA, USA, Cat#31985). Stable lentivirus transfections followed by puromycin selection to produce MCPyV integrated derivatives hPGCLC_A4_L82 and hiPSC_A4_L82 followed the standard AddGene protocol with polybrene (Santa Cruz Biotechnology, Dallas, TX, USA, Cat#sc-134220) with minor modification due to the requirement of daily Essential 8 medium change for pluripotent stem cell line hiPSC_A4. MCPyV integrated hEGCLC_A4_L82 was derived from hPGCLC_A4_L82 following the same 10-day in vitro conversion protocol [35] facilitated by SCF and FGF2, which were used for converting hPGCLCs to hEGCLCs.

### 2.3. MCPyV+ Human Primeval Stem Cell Line-Derived Mouse Xenograft Study

The aim of our teratoma assay-like mouse study was to prove MST from MCPyV transfected hPGCs to VP-MCC like tumors in the adult mouse cutaneous microenvironment. Based on prior studies on human pluripotent stem cell-induced mouse teratoma xenografts, we estimated that three mice per condition would provide a >99% success rate of forming mouse xenograft tumors [46]. Subcutaneous flank injections were performed in a total of eighteen adult male NSG mice, divided into six groups of three mice each (Table 1). Each subcutaneous injection represents an experimental unit. First, hPGCLC_A4 and its MCPyV transfected counterpart hPGCLC_A4_L82 were injected to Group #1 or Group #2 mice, respectively. Group #1 was the control group for Group #2 to confirm MCPyV-driven conversion of MST to VP-MCC-like tumors. **hPGCLCs** are derived from human pluripotent stem cells in vitro and recapitulate the early-hPGC state [47,48]. Both normal hPGCs and hPGCLCs exhibit latent pluripotency. They retain germline unipotency in vivo without chimera [32] or teratoma-forming capacity [20,49]. However, they can undergo parthenogenetic reversion to pluripotent hEGCs or hEGC-like cells (hEGCLCs) in vitro [35,50]. Due to limited culture yield, a low number (~3 × 10^5^) of cells of either hPGCLC_A4 (Figure S3A, provided by the Shioda lab [35]) or MCPyV-transduced hPGCLC_A4_L82 (Figure S3B) were injected into only the right flanks of three mice per group (*n* = 3 injections per group). Second, we want to study if MCPyV can also drive **hiPSCs**, derived from reprogramming of somatic cells and resembling hESCs [51], to form VP-MCC-like tumors. Based on our hypothesis, an indirect route from human pluripotent stem cells to VP-MCC-like tumors can theoretically be established through hPGCLC specification from competent human pluripotent stem cells [47]. Totipotent naïve hESCs of the inner cell mass at the early pre-implantation blastocyst stage and pluripotent formative-hESCs at the peri-implantation blastocyst stage are competent for hPGC induction, while more developmentally advanced pluripotent primed hESCs of the late post-implantation blastocyst stage are not [8,47]. Only a small subset of conventionally cultured hESCs or hiPSCs maintain a naïve hESC state [52], while the vast majority remain at the primed hESC state [53], reflecting developmental plasticity between totipotent and primed hESC states [1,53]. Given that only a small subset of hiPSCs is capable of hPGCLC differentiation [47,52,53], we tested dose-dependent tumorigenicity by injecting MCPyV transfected hiPSC_A4_L82 (Figure S3C) with doses of a conventional number (1 × 10^6^) and high number (2 × 10^7^) of cells into bilateral flanks of three mice as Group #3 and Group #4 (*n* = 6 injections per group). Third, we investigated whether MCPyV can drive pluripotent **hEGCLCs** to form VP-MCC-like tumors in vivo as well. hEGCLC and hEGC [35,50] are in vitro parthenogenetic derivatives of unipotent hPGCLC and hPGC, respectively. Therefore, hEGCLC serves as a surrogate for hEGC. Given that VP-MCC is a somatic cancer, and based on the normal and teratoma developmental trajectories [1], we considered the possibility that MST may require parthenogenetic reversion of an MCPyV-transformed hPGC to a pluripotent or totipotent intermediate [1], prior to neuroendocrine lineage differentiation toward the VP-MCC phenotype. This would position hEGCs as a more direct candidate cell of origin than hPGCs, supporting the inclusion of hEGCLCs to test for direct conversion to VP-MCC-like tumors in vivo. It also remained unclear whether MCPyV-transfected hEGCLC possesses equivalent developmental potency to non-parthenogenetic human pluripotent stem cell counterparts like MCPyV-transfected hiPSCs, particularly in achieving a naïve hESC state to support hPGCLC induction and thus an indirect MST route to VP-MCC-like tumors. To evaluate both possibilities, MCPyV-transfected hEGCLC_A4_L82 was injected at both cell doses (1 × 10^6^ and 2 × 10^7^) into bilateral flanks of three mice per group for Group #5 and Group #6 (*n* = 6 injections per group), respectively. For Groups #3 and #4 or Groups #5 and #6, no corresponding MCPyV-negative (hiPSC_A4 or hEGCLC_A4) control groups were included in the mouse study as it has been well established that pluripotency alone is coupled to teratoma formation without MST in teratoma assays [18]. The decision to exclude these negative control groups was therefore made in accordance with the 3R principle outlined in the *Guide for the Care and Use of Laboratory Animals* and the ARRIVE guidelines in order to minimize the number of mice used without compromising scientific rigor.

All animals used in this study were handled in accordance with an animal study protocol (PAT-24-157) approved by the Uniformed Services University of Health Sciences Institutional Animal Care and Use Committee. As MCC occur in adults with a male dominance, 4- to 6-week-old adult and male immunocompromised NSG mice [NOD.Cg PrkdcscidIl2rgtm1Wjl/SzJ, Jackson Laboratory, Farmington, CT, USA, Stock# 005557] were used for the study. All mice were housed in an immunocompromised mouse room using microisolator individually ventilated cages, with a minimum acclimation period of three days. Simple physical restraint with the double-hand method was used for injecting cells subcutaneously into mouse flank. Mice were monitored regularly for health status and were sacrificed by CO_2_ asphyxiation upon reaching humane endpoint criteria. All human primeval stem cells, including hPGCLC, hiPSC, and hEGCLC, were suspended in 50 ul with 10 µM ROCK inhibitor Y-27632 (StemCell Technologies, Vancouver, BC, Canada, Cat#72304) and mixed with an equal volume of Matrigel (Corning, New York, NY, USA, Cat#354277) before injected subcutaneously into the flanks of the mice [35,46]. Mice were monitored regularly for tumor development and sacrificed upon reaching humane endpoint criteria. Tumors were collected by dissection. Friable portions of tumors were collected in sterile RPMI 1640 media (Gibco, Brooklyn, NY, USA, Cat#11875093) to prepare cell suspension by mechanical disruption using scissors and forceps. Cell pellets obtained by centrifugation at 400× *g* were then used for both single cells suspension for flow cytometry and to extract total RNA using the PureLink RNA extraction mini kit (Thermo Fisher, Waltham, MA, USA, Cat#2592159), as well as cryopreserved in FBS (Sigma, Burlington, MA, USA, Cat#F2442-500ML) with 8% DMSO (Thermo Fisher, Waltham, MA, USA, Cat#M3148-250 mL). Cryopreserved tumor cells were later thawed to extract DNA using the PureLink™ Genomic DNA Mini Kit (Thermo Fisher, Waltham, MA, USA, Cat#182001). The rest of the tumors were fixed using 10% neutral buffered formalin (Azer Scientific, Morgantown, WV, USA, Cat#NBF-1-G) and subjected to standard H&E as well as IHC staining. Cell line authenticity for all VP-MCC-like tumor (+) tumors was confirmed by no variation at any loci of five mononucleotide repeats and two pentanucleotide repeats (NR-21, BAT-26, BAT-25, NR-24, MONO-27; Penta C, Penta D) among themselves and all three human primeval stem cell lines (hPGCLC_A4_L82 and hiPSC_A4_L82) using the Promega OncoMate MSI Analysis System (Promega, Madison, WI, USA, Cat. # MD2140) and DNA extracted from the tumor cells and cell lines (Figure S21).

### 2.4. RT-qPCR

Total RNA of cell lines was also extracted using the PureLink RNA extraction mini kit (Thermo Fisher, Waltham, MA, USA, 2592159). Reverse transcription was performed using Luna one-step RNA-to-Ct RT-qPCR kit (New England Biolabs, Ipswich, MA, USA, Cat#E3005E), 5 µM gene-specific primer pairs, 5 ng RNA per final 20-µL RT-qPCR reaction, and the Azure Ciel 3 qPCR detection system (Azure Biosystems, Dublin, GA, USA) with the cycling/melting curve RT-qPCR program recommended by the manufacturers. All primer sequences are listed in Table S4. mRNA expression of each gene was normalized relative to the mRNA expression of housekeeping gene RnaseP as ΔCq. All RT-qPCR experiments were repeated at least three times.

### 2.5. Immunohistochemistry (IHC) Study

Sections of xenograft mouse tumors or pellets of cell lines were first deparaffinized by incubating in xylene, followed by rehydration with ethanol solutions (100% and 95%) and distilled water. Antigen retrieval was performed using EDTA Antigen Retrieval Buffer (pH 8.0, 10X) (Vitro Vivo Biotech, Rockville, MD, USA, Cat#VB-6007) in a steamer, with sections boiled for 40 min–1 h in 1X EDTA Buffer (Table S6). After washing, endogenous peroxidase activity was blocked with 3% hydrogen peroxide prepared from 30% hydrogen peroxide solution (Lab Alley, Austin, TX, USA, Cat#HPL30), and sections were then blocked with 5% goat serum (Gibco, Brooklyn, NY, USA, Cat#16210064) in TBST buffer made from QuickSilver Powdered Buffer Packs (Accuris Instruments, Edison, NJ, USA, Cat#EB-1203) for 1 h. Primary antibodies were diluted based on manufacturer’s recommendation and empirical testing. All primary antibodies’ host animals, manufacturers, catalogue numbers, dilution factors, and incubation times are listed in Table S6. The Rabbit IgG isotype control (Invitrogen, Carlsbad, CA, USA, Cat# 31235) or mouse IgG isotype control (Invitrogen, Carlsbad, CA, USA, Cat#31903) was diluted to the same final concentrations as the corresponding primary antibodies to act as negative controls. If the primary antibodies are of rabbit host, they were incubated overnight at 4 °C with the only exception being the 5mC antibody (Invitrogen, Carlsbad, CA, USA, Cat#MA5-24694), which was incubated for one hour at room temperature. Following antibody incubation, SignalStain^®^ Boost IHC Detection Reagent (HRP, Rabbit) (Cell Signaling Technology, Danvers, MA, USA, Cat#8114S) was applied per the manufacturer’s instruction, and sections were incubated for 30 min. However, if the primary antibodies were of mouse host, to exclude false positive signal from mouse tissue, the sections were stained for primary and secondary antibodies by M.O.M.^®^ (Mouse on Mouse) ImmPRESS^®^ HRP (Peroxidase) Polymer Kit (Vector Laboratories, Burlingame, CA, USA, Cat#MP-2400) per the manufacturer’s protocol. SignalStain^®^ DAB Substrate Kit (Cell Signaling Technology, Danvers, MA, USA, Cat#8059S) was applied to visualize the antigen–antibody complex, and counterstaining was performed with hematoxylin (Cell Signaling Technology, Danvers, MA, USA, Cat#14166S). The sections were subsequently dehydrated using 95% and 100% ethanol followed by xylene before being mounted with SignalStain^®^ Mounting Medium II (Cell Signaling Technology, Danvers, MA, USA, Cat#84583S) under a coverslip. Semi-quantitative 5mC IHC stains were performed three times on cell lines, tumor sections, mouse somatic tissue positive controls, and isotype negative controls.

### 2.6. Flow Cytometry 5mC Analysis for Assessment of Global DNA Methylation

Single cell suspensions were prepared for VP-MCC-like tumor (+) mouse tumors, VP-MCC cell lines, and hiPSC_A4_L82 line, as well as VN-MCC cell lines and TCam2 cell line via different methods. Single-cell suspensions were made for friable mouse VP-MCC-like tumors by mechanical disruption with scissors and forceps in sterile RPMI 1640 media (Gibco, Brooklyn, NY, USA, Cat#11875093). Single-cell suspensions of the floating VP-MCC cell lines and attached hiPSC_A4_L82 line required cell separation by incubation with ReLeSR™ (StemCell Technologies, Vancouver, BC, Canada, Cat#100-0483) for 20+ min and up to 10 min at 37 °C, respectively. Single-cell suspensions of attached VN-MCC cell lines and TCam2 cell line required cell separation by incubation with 1.25% trypsin (Gibco, Brooklyn, NY, USA, Cat#15090046) for up to 8 min. All separated cells were then filtered through 100 um cell strainers (Ibis Scientific, Las Vegas, CA, USA, Cat#229486). Prepared single cell suspensions were then stained with eBioscience™ Fixable Viability Dye eFluor™ 780 (Invitrogen, Carlsbad, CA, USA, Cat#65-0865-14) for 30 min in a blocking buffer of 2% FBS (MilliporeSigma, Burlington, VT, USA, Cat#F2442-500ML) in PBS (Cytiva, Marlborough, MA, USA, Cat#SH30256.02). eBioscience™ Foxp3/Transcription Factor Staining Buffer Set (Invitrogen, Carlsbad, CA, USA, Cat#00-5523-00) was used for cell fixation and antibody staining per the manufacturer’s protocol. Briefly, the cell suspensions were fixed and stained with 5-methylcytosine (5mC) recombinant rabbit monoclonal antibody (RM231) (Invitrogen, Carlsbad, CA, USA, Cat#MA5-24694) at 1:1000 final dilution and/or the rabbit IgG isotype control (Invitrogen, Carlsbad, CA, USA, Cat#31235) at the same final concentration for 30 min on ice, followed by staining with goat anti-rabbit IgG (H+L) highly cross-adsorbed secondary antibody with Alexa Fluor™ 790 (Invitrogen, Carlsbad, CA, USA, Cat#A11369) at 1:500 final dilution for 30 min. The cells were then stained with Alexa Fluor™ 594-conjugated rabbit Sox2 antibody (Santa Cruz Biotechnology, Dallas, TX, USA, Cat# sc-365823) for 30 min on ice. Cell suspensions were then placed in the eBioscience™ Flow Cytometry Staining Buffer (Invitrogen, Carlsbad, CA, USA, Cat#00-4222-26) and analyzed using a Cytek Aurora flow cytometer (Fremont, CA, USA). The FlowJo_v10 Software (BD Biosciences, San Diego, USA) was used to analysis flow data by gating the Sox2(+) population for VMLT cells, and the median fluorescence intensity (MFI) of Alexa Fluor™ 790 as well as the standard error of the mean (SEM) were calculated using FlowJo_v10.

### 2.7. Statistical Analysis

All RT-qPCR experiments were repeated at least three times. For semi-quantitative 5mC flow cytometry, multiple paired MFI/SEM (median/CV) results calculated by FlowJo software for each sample were consolidated to reach a combined MFI and combined SEM by the pooled variance using the law of total variance. The pooled variance is a weighted average of the variances in different groups and reflects both within-and between-group variability [54]. Briefly, if n is the number of MFI/SEM pairs measured per sample, MFI_combined_ = (MFI_1_ + MFI_2_ + … MFI_n_)/n, SDi = SEMi × MFIi, SD_combined_ = square root [(SD_1_^2^ + SD_2_^2^ +…+ SD_n_^2^)/n], and SEM_combined_ = SD_combined_/MFI_combined_.

For group comparison, *p* values were calculated by either paired or unpaired one tail Student *t*-test. A *p* value with <0.05 was considered statistically significant.

Based on a binomial distribution of single injection success rate of 80–100% for teratoma formation from subcutaneous injection of human pluripotent stem cells mixed with Matrigel [46], we estimated three mice per group to be the minimum number to ensure the achievement of a high success rate (99.2%) of tumor formation.

## 3. Results

Tumors containing both VP-MCC–like and teratoma components developed exclusively from low-number (3 × 10^5^) hPGCLC_A4_L82 injections and high-number (2 × 10^7^) hiPSC_A4_L82 injections. In contrast, only teratoma components formed in mice injected with the conventional number (1 × 10^6^) of hiPSC_A4_L82 cells, as well as in all hEGCLC_A4_L82-injected mice, regardless of cell number (1 × 10^6^ or 2 × 10^7^). No tumors were observed in MCPyV-negative hPGCLC control mice (Table 2 and Table S1), consistent with their known unipotency in vivo, as previously reported [20,49]. Results were fully reproducible across all three mouse replicates in all six experimental groups, with the sole exception of Group #2: three mice injected with MCPyV-transfected hPGCLC_A4_L82. In this group, two mice developed tumors containing both VP-MCC-like and teratoma components, while the third mouse (#86.3) failed to develop any tumor, including a teratoma. We interpret the absence of tumor formation in this third hPGCLC_A4_L82-injected mouse as most likely due to failed injection or engraftment, rather than reflecting a less robust MST potential due to the absence of teratoma. Our records indicated that this mouse (86.3) was especially difficult to handle during injection. Moreover, the injected cell number (3 × 10^5^) was substantially lower than the conventional 1 × 10^6^ used in standard teratoma assays, which may have contributed to suboptimal engraftment. The absence of teratoma thus supports the interpretation of technical failure in the third mouse (86.3). When using teratoma formation as an indication of successful engraftment, our success rate for MST was thus 100% (2/2). The robustness of our MST model was further demonstrated by the 100% success rate (6/6) of VP-MCC–like tumor formation in addition to a teratoma component from all high-number hiPSC_A4_L82 injections. All bilateral injections of hiPSC_A4_L82 line or hEGCLC_A4_L82 line in the same mouse resulted bilateral mouse xenograft tumors of essentially the same gross and histologic characteristics (Table 2 and Table S1).

There was no gross or histological evidence of distant metastasis from any xenograft tumors with VP-MCC-like tumors. This finding is consistent with the known much less aggressive phenotype with only rare distant metastasis from VP-MCC tumor cell line-derived mouse tumors in prior studies [55]. The reason for the marked difference of VP-MCC tumors’ aggressiveness in human versus mouse is unknown and may reflect species differences.

### 3.1. Confirmation of MST to VP-MCC-like Tumors

#### 3.1.1. Morphology Confirmation of MST to VP-MCC-like Tumors

**Figure 1:**
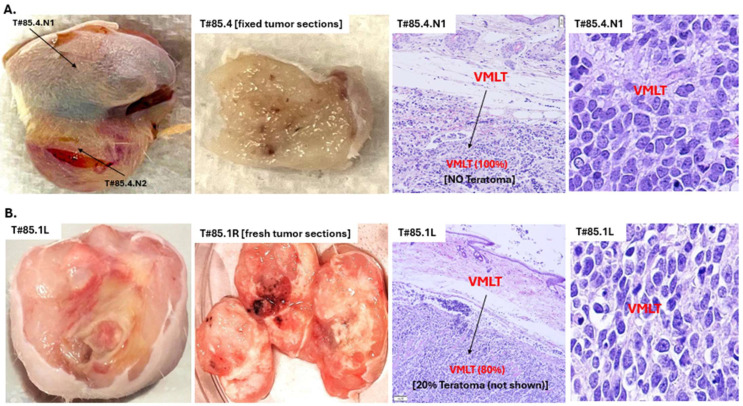
Gross appearance and histology of representative mouse VP-MCC-like tumors (VMLTs) derived from human primeval stem cells. Injected with hPGCLC_A4_L82 (~3 × 10^5^ cells): (**A**) Mouse #85.4. Injected with hiPSC_A4_L82 (~2 × 10^7^ cells): (**B**) Mouse #85.1. Each mouse was identified by a unique number based on the cage number it resided in followed by an individual ID number (Cage.ID). The tumors formed in a mouse were identified as T followed by the mouse’s unique number. If there were bilateral tumor formation, R for the right side and L for the left side were added at the end (T#Cage.ID.R or L). For any tumor contained multiple discrete tumor nodules, N followed by a tumor nodule number was added at the end of the tumor’s name, i.e., T#85.4.N1 or T#85.4.N2.

Grossly, VP-MCC-like tumor (+) tumors, which were composed of both VP-MCC-like tumor and teratoma components, were markedly different from VP-MCC-like tumor (−) tumors, which were composed only of a teratoma component. All VP-MCC-like tumor (+) tumors were solid and homogeneous with pale and friable texture grossly (Figure 1 and Figure S4), while teratoma-only tumors were cystic and mucinous with heterogeneous discoloration (Figures S5–S7). The finding mimicked the typical gross difference between clinical teratomas with MST and pure teratomas [56]. The WHO criteria for MST in human GCTs require a minimum 5 mm expansion of pure somatic type of malignancy component [57]. Our most salient tumor examples of MST to VP-MCC-like tumor were T#85.4, consisting of two discrete tumor nodules with the larger nodule (T#85.4.N1 ~20 mm) composed of pure VP-MCC-like tumor tissue (Figure S8A), and T#84.5R ~20 mm, composed almost entirely of VP-MCC-like tumor (Figure S9A,B). The rest of the VP-MCC-like tumors also occupied substantial portions of tumors in sizable continuous sheets (~20%–~80% of tumor masses) (Figures S9C, S10, S11, S12A and S13A).

Histologically, VP-MCC-like tumor (+) tumors exhibited a distinct overall spatial pattern: primitive VP-MCC-like tumor components localized superficially and peripherally, surrounding more differentiated teratoma components located deeper and centrally (Figures S9C, S10, S12A, S13A and S24). This organization contrasts with the typical “differentiation gradient” of human pluripotent stem cell-derived mouse xenograft pure teratomas like VP-MCC-like tumor (−) tumors, which display a less differentiated core and more mature peripheral regions [20]. Notably, the teratoma components within VP-MCC-like tumor (+) tumors did retain the expected maturation gradient of xenograft pure teratomas (Figure S24A,B). The opposing spatial arrangements of the overall VP-MCC-like tumor (+) tumors versus their teratoma components implicate a distinct VP-MCC-like tumor tumorigenic pathway requiring proximity to the cutaneous microenvironment.

#### 3.1.2. VP-MCC Like Histopathology of VP-MCC Like Tumors

Histologic sections and cytology smears of all VP-MCC-like tumors showed sheets of immature small blue cells with either the typical Merkel cell carcinoma small round cell cytomorphology in the two hPGCLC_A4_L82 derived tumors T#85.3 or T#85.4 (Figure 1, Figures S4, S8E, S11E and S14) or the atypical Merkel cell carcinoma large cell variant with admixed larger cells exhibiting prominent nucleoli and more abundant cytoplasm in all six high-number hiPSC_A4_L82 derived tumors, including T#84.5L and R, T#85.1L and R, and T#85.2L and R (Figure 1, Figures S4, S9F, S12D, S13E and S14). All VP-MCC-like tumors exhibited prominent histological characteristics of malignancy (Figure S15), as a functional indicator of its aggressive nature. These malignant features include extracapsular invasion into dermis, with destruction of dermal striated muscle layer and lymphovascular invasion, with VP-MCC-like tumor cells within lymphatic spaces (Figures S9E, S10, S11A,D, S12B,C and S13B–D).

#### 3.1.3. VP-MCC Like Molecular Profile of VP-MCC Like Tumors

**Figure 2:**
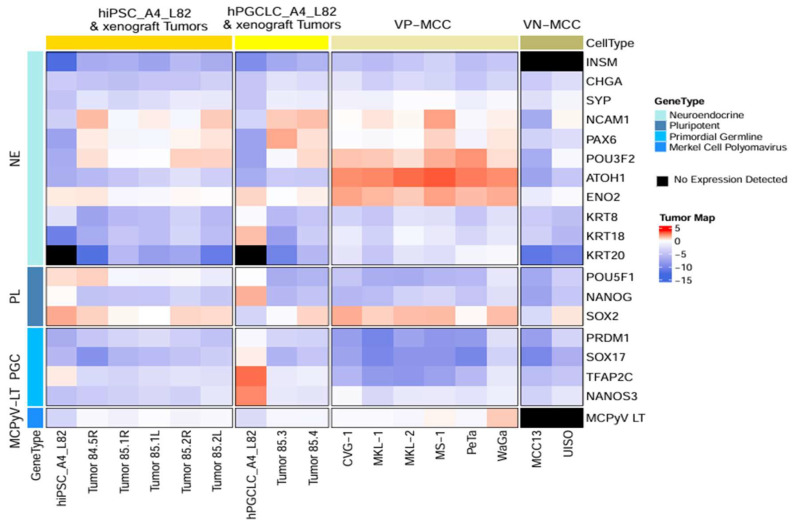
Heatmap of mRNA expression. Comparison of mRNA expression of key neuroendocrine lineage genes, core pluripotent genes, PGC marker genes, and MCPyV LT viral oncogene among transfected primeval stem cell lines (hiPSC_A4_L82 and hPGCLC_A4_L82), their derivative mouse xenograft tumors, and VP-MCC and VN-MCC cell lines.

**Figure 3:**
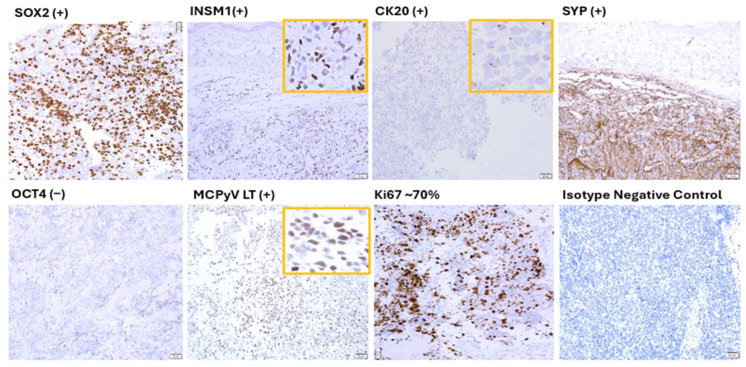
Representative immunohistochemical studies of mouse hPGCLC_A4_L82 derived VP-MCC-like tumor of T#85.4.N1. VP-MCC-like tumor of T#85.4 showed positive protein expression of SOX2, INSM1, CK20 (dot-like), synaptophysin, and viral MCPyV LT antigen, as well as negative protein expression of OCT4, characteristic of VP-MCC. Ki67 IHC stain highlighted a high proliferative index defining all high-grade neuroendocrine carcinomas like VP-MCC. Protein expressions of additional genes in T85.4.N1 are shown in Figures S16 and S17.

Molecular characterization by RT-qPCR (Figure 2, Table S2) and/or immunohistochemical (IHC) studies Figure 3 and Figures S16–S21, Table S3) of VP-MCC-like tumors confirmed the high-grade neuroendocrine tumor phenotype with expression of SOX2 and numerous neuroendocrine lineage gene as well as cytokeratin, including CK20. VP-MCC-like tumors, derived either from hiPSC_A4_L82 or hPGCLC_A4_L82, showed highly similar RT-qPCR gene expression profiles to each other (Table S2C). All VP-MCC-like tumors showed mRNA gene expression profile in neuroendocrine lineage genes, viral LT antigen, core pluripotency gene, and PGC similar to that of VP-MCCs with few exceptions (Table S2B) as well as a marked departure from that of the corresponding MCPyV-transfected primeval stem cells, hPGCLC_A4_L82, or hiPSC_A4_L82 (Figure 2 and Figure 3, Figures S16–S21, Tables S2D and S3). There is no statistically significant difference (*p* = 0.19, Table S2B) between the overall *p* values for all VMLTs vs. VP-MCC lines’ gene expression profile by RT-qPCR and the overall *p* values for that of all VMLTs vs. VN-MCC lines, indicating VP-MCC-like tumors are about equally similar to VP-MCC lines and VN-MCC lines in terms of the overall gene expression profile tested by RT-qPCR. However, the most sensitive and specific neuroendocrine gene INSM1 was expressed only in VP-MCC-like tumors and VP-MCC lines, but not in VN-MCC lines (Figure 2). There were only two significant exceptions in terms of highly similar gene expression profiles between VP-MCC-like tumors and VP-MCC lines. The high Oct4 expression of all hiPSC_A4_L82-derived VP-MCC-like tumors, in contrast to very low or absent expression in VP-MCC lines or hPGCLC_A4_L82 derived VP-MCC-like tumors (Figures S19–S21, Tables S2C,D and S3), raised concerns about a possible embryonic carcinoma component in these VP-MCC-like tumors. However, it was ruled out by PLAP (-) and CD30 (-) IHC stains on these VP-MCC-like tumors (Figure S22). The second exception was the significantly lower ATOH1 mRNA levels (*p* = 4.38 × 10^−7^, Table S2B) in all VP-MCC-like tumors than VP-MCC cell lines, which might be due to the incipient tumor status of VP-MCC-like tumors versus the advanced cancer stage of these VP-MCC cell lines [58].

### 3.2. Global DNA Methylation Reset Before MST

In vitro models of primeval stem cells mirror their developmental states with well-characterized global DNA methylation levels. hiPSCs and hEGCLCs (primed hESC-like) show high global DNA methylation (~70%), whereas hPGCLCs (early hPGC-like) show intermediate global DNA methylation (~50%) [35] (Figure S1). Germ cell cancers typically reflects this epigenetic hierarchy with few exceptions. Seminomas usually match the extremely low global DNA methylation of late-hPGCs, embryonic carcinomas usually show moderate global DNA hypomethylation of naïve hESCs, and malignant teratomas usually exhibit higher global DNA methylation due to somatic differentiation [1]. All germ cell cancers thus retain variable degrees of global DNA hypomethylation consistent with their late-hPGC origin [1]. Somatic cancers are also known to exhibit global DNA hypomethylation, positively correlating with degree of malignancy and cancer progression [16]. Small cell neuroendocrine carcinomas, including Merkel cell carcinoma, share marked global DNA hypomethylation [59,60] with seminomas and embryonic carcinomas, which are the stem-like compartments of germ cell cancers. They also share stemness and developmental plasticity. To investigate further, we compared global DNA methylation in VP-MCC-like tumors, Merkel cell carcinoma lines, and the only validated seminoma cell line TCam-2 [45]. One important caveat is that DNA methylation is highly sensitive to the microenvironment, and tumor cells maintained in vitro often display artificially elevated global DNA methylation [61]. For instance, like all seminomas, the only validated seminoma cell line TCam-2 is composed of transformed late-hPGCs, which are known to exhibit extremely low global DNA methylation (~6% median) [33,34]. However, the TCam-2 cell line exhibits a moderate global DNA methylation level [62] comparable to the cell lines of embryonic carcinoma mirroring naïve hESC/inner cell mass state^1^ (global DNA methylation ~40%) [33,34], rather than the extreme global hypomethylation observed in primary seminoma tissues [62], likely reflecting culture-induced epigenetic drift [61].

#### 3.2.1. Global DNA Methylation Assessment Using 5mC Flow Cytometry and Validation Using IHC

**Figure 4:**
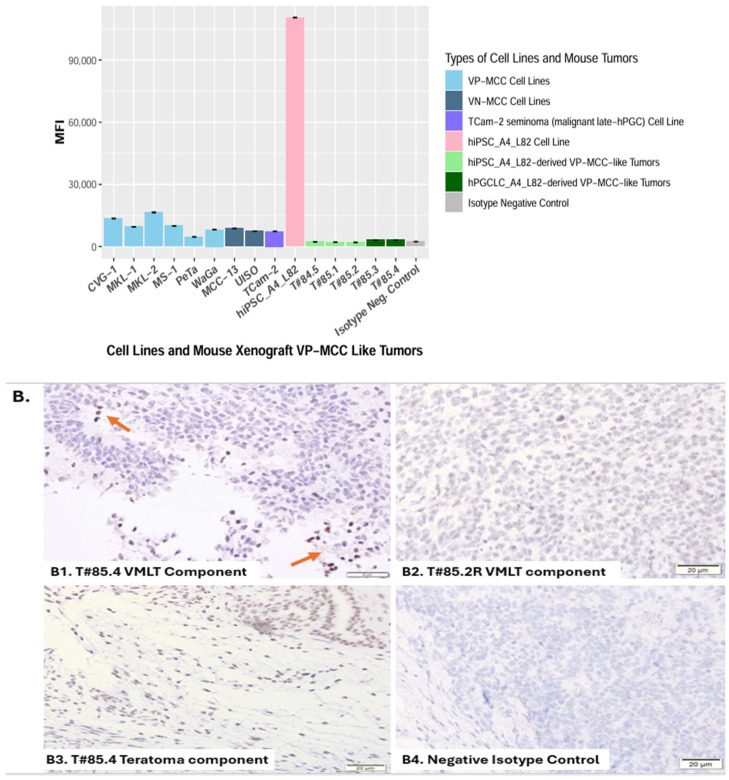
Global 5mC levels to estimate global DNA methylation using (**A**) flow cytometry and (**B**) immunohistochemical (IHC) studies. (**A**) Flow cytometry estimated global 5mC levels semi-quantitively for the hiPSC_A4_L82 cell line, seminoma cell line TCam2, VP-MCC-like tumors from five mice, six VP-MCC cell lines, two VN-MCC cell lines, and a composite isotype control derived from multiple above cell lines. Global 5mC levels were measured as median fluorescence intensity (MFI) with error bars indicating standard error of the mean (SEM). hiPSC_A4_L82 line and seminoma cell line Tcam2 exhibited very high (~110 K) and low (~7 K) global 5mC levels indicating global DNA methylation levels consistent with previous studies [35,62]. The composite isotype negative control (Table S4) exhibited an extremely low (~2.4 K) global 5mC level as the background signal for absence of global DNA methylation. This was similar (*p* = 0.38, Table S4B) to that of VP-MCC-like tumors indicating nearly complete global DNA methylation erasure. VP-MCC and VN-MCC cell lines showed low global 5mC levels similar to that of the TCam-2 cell line, which is composed of transformed late-hPGCs (Table S4A). (**B**) Global 5mC IHC studies of VP-MCC-like tumor components either derived from hPGCLC_A4_L83 (T#85.4, B1) or hiPSC_A4_L82 (T#85.2R, B2) and negative isotype control (B4), as well as the teratoma component from VP-MCC-like tumor (+) tumors T#85.4 (**B3**). VP-MCC-like tumors from T#85.4 (**B1**) and T#85.2R (**B2**) showed near negative 5mC IHC stains resembling the isotype negative control (**B4**), confirming the flow cytometry findings in 4A. The teratoma component from T#85.4 (**B3**) served as a positive control due to its more advanced somatic differentiation. In addition, a few scattered and positively stained mouse somatic white blood cells (**red arrows** in **B1**) also served as internal positive controls, in contrast to the neat negative staining of VP-MCC-like cells (**B1**).

5-Methylcytosine (5mC) is widely accepted as a surrogate marker for global DNA methylation levels, as it represents the most common and stable epigenetic modification of cytosine residues within the genome, particularly at CpG dinucleotides. Quantification of total 5mC content provides a reliable estimate of genome-wide methylation status across different cell types and developmental stages [63,64]. Semi-quantitative flow cytometry and immunohistochemical (IHC) studies to measure global 5-methylcytosine (5mC) levels for assessing global DNA methylation were validated using reference cell lines with well-characterized global DNA methylation profiles. The hiPSC_A4_L82 line, modeling the primed hESC state known to exhibit high somatic-type global DNA methylation (~70%) [35], showed a very high 5mC signal (MFI ~ 110 K) with flow cytometry. The seminoma cell line TCam-2, which reflects a moderately hypomethylated state similar to embryonic carcinomas or naïve hESCs (global DNA methylation ~ 40%) [33,34], displayed a much lower (*p* = 3.45 × 10^−9^, Table S4B) 5mC signal (MFI ~ 7 K) using flow cytometry. Isotype negative controls of various cell lines, including TCam2 line, yielded minimal signals (MFI ~ 1.6 K~3.2 K) and were combined to form a composite isotype control (~2.4 K, Table S4C) as the background reference for absence of global DNA methylation (Figure 4A; Table S4). IHC stains of hiPSC_A4_L82 line, Tcam2 line, and its isotype negative control showed similar trend (Figure S23). Positive IHC stain of pure teratoma from T#83.5 served as a positive control as well (Figure S23).

#### 3.2.2. Requisite Late-hPGC State Before MST to VP-MCC-like Tumors

- VP-MCC-like tumors exhibited unique late-hPGC global DNA methylation signature

**Figure 5:**
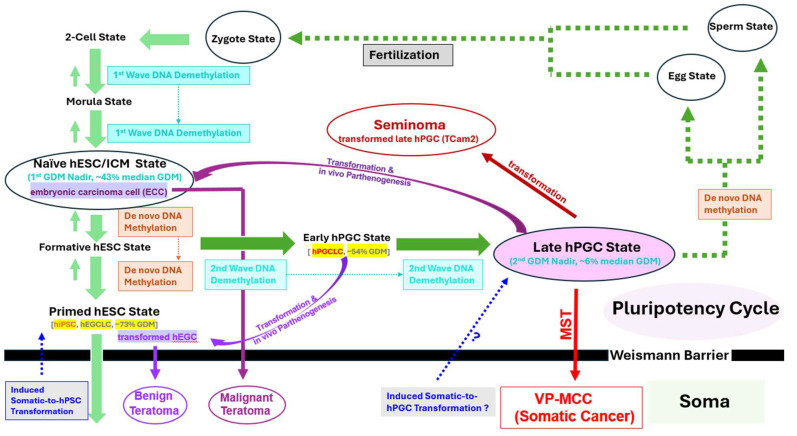
Schematic of normal human embryonic and germline development in comparison to the carcinogenesis of somatic VP-MCC and germ cell tumors. Light green arrows show normal early embryogenesis from fertilization to primed pluripotent hESCs before crossing the Weismann barrier into somatic differentiation to form the soma. The intrinsic plasticity between the two-cell stage and the primed hESC stage is reflected in the bidirectionality of the light green arrows [1]. Normal human germline development (dark green arrows) begins at the specification of hPGCs from formative hESCs and ends with the formation of sperm or eggs, guided by an intrinsic program suppressing somatic fate, yet exhibiting latent/poised pluripotency [32] with the germline being the only human cell lineage capable of physiological conversion to pluripotency (parthenogenesis) without genetic manipulation. Thus, the germline cycle may be considered a “pluripotency cycle” [32], consisting of both early embryogenesis (light green arrows) and germline development (dark green arrows), which are separated from the soma compartment by the Weismann barrier. Based on our mouse model, carcinogenesis of somatic VP-MCC (red straight arrow) also crosses the Weismann barrier to the somatic compartment, like teratomas which are a type of non-seminomatous GCT (dark purple and light purple straight arrows), and normal human embryogenesis [1]. However, unlike teratomas or embryogenesis, VP-MCC tumorigenesis crosses the Weismann barrier directly from the germline (late hPGCs) to the somatic side without a pluripotent intermediary, resulting in mono-lineage (neuroendocrine) somatic differentiation accompanied by developmental arrest. In contrast, tumorigenesis of malignant teratomas more closely mirrors normal embryogenesis, requiring a totipotent embryonic carcinoma cell intermediate to cross the Weismann barrier and enable somatic differentiation of three germ layers. These totipotent stem cells of malignant teratomas are believed to arise via parthenogenetic transformation of transformed hPGCs in vivo (dark purple and light purple curved arrows), rather than from fertilization-derived zygotes. Seminomas, another malignant germ cell tumor subtype, consist of transformed late hPGCs and therefore retain the epigenetic signatures characteristic of this stage [1]. Two waves of global DNA demethylation occur during development, each followed by de novo methylation. The first wave begins at the two-cell stage and reaches its bottom at the naïve hESC/inner cell mass stage, with partial erasure of global DNA methylation (~40%) [33,34]. The second, germline-specific wave occurs only during hPGC development, resulting in near-complete global DNA methylation erasure and reaching the lowest bottom at the late hPGC stage (global DNA methylation ~6%) [33,34]. The moderate global hypomethylation observed in two variant VN-MCC cell lines like that of seminoma cell line TCam2 and VP-MCC lines suggests a possible somatic-to-hPGC transformation (slanted dashed blue arrow with a question mark), potentially triggered by somatic mutations or other mechanisms. The somatic to hPGC transformation may also undergo indirect pathway through reprogramming of somatic cells into hiPSCs followed by specification into a hPGC-like state (vertical dashed blue arrow). The three human primeval stem cell types used in our mouse model, including hPGCLCs (modeling early hPGCs), hiPSCs (modeling primed hESCs), and hEGCLCs (modeling hEGCs), are highlighted in yellow.

Flow cytometry analysis of VP-MCC-like tumor cells revealed extremely low global 5mC levels (MFI: ~2 K–~3.1 K) (Figure 4A, Table S4), approaching (*p* = 0.38, Table S4B) the isotype negative controls (composite MFI: ~2.4 K). Global 5mC IHC studies of VP-MCC-like tumor (+) tumors T#85.4 and T#85.2R showed near negative staining for the VP-MCC-like tumor components (Figure 4B1,B2) and positive staining for the teratoma component (Figure 4B3). This is similar to pure teratoma from VP-MCC-like tumor (−) tumor T#83.5L (Figure S23B. These results thus indicated VP-MCC-like tumors exhibited unique late-hPGC global DNA methylation signature with near-complete global DNA methylation erasure, which suggests a model in which MST occurs directly from the late-hPGC state across the Weismann barrier, bypassing any intermediary stage marked by moderate to high global DNA methylation (Figure S1 and Figure 5). The VP-MCC-like tumors were derived either from hPGCLC_A4_L82 (modeling early hPGCs with ~50% global DNA methylation) [35] or from hiPSC_A4_L82 (modeling primed hESCs with ~70% global DNA methylation) [35], confirming a profound global DNA methylation reset preceding MST to VP-MCC-like tumors.

- Direct and indirect tumorigenic pathways to VP-MCC-like tumors

VP-MCC-like tumors’ late-hPGC global DNA methylation signature enables delineation of the tumorigenic pathways for VP-MCC-like tumors derived from hPGCLC_A4_L82 versus those derived from high-number hiPSC_A4_L82 cells. A direct pathway, for hPGCLC_A4_L82-derived VP-MCC-like tumors, involved further germline progression from an early-hPGC like state (modeled by hPGCLC_A4_L82) [35] to the requisite “late-hPGC” state in vivo, followed by MST to VP-MCC-like tumors (Figure 5). In contrast, an indirect pathway, for hiPSC_A4_L82-derived VP-MCC-like tumors, necessitated an extra initial hPGCLC specification step, in which a small subset of naïve hiPSC_A4_L82 cells first differentiated into hPGCLC_A4_L82 cells. These then proceeded to the “late-hPGC” state before undergoing MST to VP-MCC=like tumors (Figure 5). The observed dosage effect–VP-MCC-like tumor formation with a high number (2 × 10^7^) but not conventional number (1 × 10^6^) of hiPSC_A4_L82 injections, with no such effect in low-number (3 × 10^5^) hPGCLC_A4_L82-derived tumors. This can be attributed to this additional hPGCLC specification bottleneck. Moreover, clinical observations of rare MST from pure seminomas to somatic malignancies [65], including high-grade neuroendocrine carcinoma intermixed with seminoma cells [66], offer further clinical support for the plausibility of direct MST from a “late-hPGC”-like state to somatic cancer, reinforcing the pathway proposed here.

#### 3.2.3. Merkel Cell Carcinoma Lines and a Seminoma Line Show Similar Global DNA Methylation

- VP-MCC and TCam2 lines showed similar global DNA methylation

To further validate the findings supporting VP-MCC tumorigenesis via direct transformation from an obligatory “late-hPGC” state, we analyzed global 5mC levels in six VP-MCC cell lines and compared them to the seminoma cell line TCam-2, which consists of transformed late-hPGCs (1). Flow cytometry showed that VP-MCC lines exhibited moderate global DNA hypomethylation (MFI: ~5–16 K), similar (*p* = 0.44, Table S4B) to TCam-2 (~7 K), though slightly higher (*p* = 0.0013, Table S4B) than VP-MCC-like tumors (~2–3.1 K) (Figure 4A; Table S4). As both VP-MCC lines and TCam-2 were cultured under identical conditions (RPMI 1640 media (Gibco, Brooklyn, NY, USA, Cat#11875093) + 10% FBS (Sigma, Burlington, VT, USA, Cat#F2442-500ML)), their mildly elevated 5mC levels relative to that of VP-MCC-like tumors likely reflect epigenetic drift due to in vitro culture conditions as well [61].

- Variant VN-MCC and TCam2 lines also showed similar global DNA methylation

Interestingly, the two variant VN-MCC cell lines, MCC-13 and UISO, grown under the same culture condition also displayed comparable levels (*p* = 0.52, Table S4B) of moderate hypomethylation (MFI: ~7–~9K; Figure 4A, Table S4B) to that of VP-MCC cell lines, aligning with previous findings [59,60]. The similarity in global DNA methylation between variant VN-MCC lines and TCam-2 under matched culture conditions suggests that primary variant VN-MCC tumors may also exhibit extreme hypomethylation which is an epigenetic hallmark of the “late-hPGC” state observed in primary seminomas. In the case of variant VN-MCCs, this “late-hPGC”-like state may arise via reprogramming from somatic cells driven by extensive somatic mutations, including pRB and p53 mutations, characteristic of these tumors [15,23,60] (Figure 5).

## 4. Discussion

### 4.1. Functional Evidence Against Parthenogenic Intermediates for MST to VMLT

#### 4.1.1. Failure of hEGCLC_A4_L82 to Derive VP-MCC-like Tumor Supports No Pluripotent Intermediate for MST

Failure of hEGCLC_A4_L82 to form VP-MCC-like tumors in vivo indicating a lack of direct conversion to VP-MCC-like tumors from hEGCLC_A4_L82, which is the in vitro pluripotent derivative of hPGCLC_A4_L82. This finding therefore provides functional evidence against the involvement of a parthenogenetic pluripotent hESC-like intermediate in MST to VP-MCC-like tumors and supports a VP-MCC-like tumor tumorigenic pathway that is independent of teratoma formation and uncoupled from parthenogenetic pluripotency.

#### 4.1.2. Decreased Potency of hEGCLC_A4_L82 Suggests No Totipotent Intermediate for MST

Previous studies showed dichotomous parthenogenic paradigms for normal hPGCs vs. transformed hPGCs into non-seminomatous germ cell cancers [1,20,35]. The developmental potency of parthenogenetic derivatives of normal hPGCs, such as hEGCLCs or hEGCs, has been shown to decrease along the hPGC developmental trajectory. hEGCs derived from post-migratory late-hPGCs lose bona fide pluripotency, whereas hEGCLCs originating from hPGCLCs (modeling early hPGCs) regain pluripotency, as demonstrated by their inability or ability to form teratomas in vivo, respectively [20,35]. Thus, prior entry into the germline and a corresponding decline in developmental potency define the normal hPGC parthenogenesis paradigm. In contrast, transformed hPGCs in non-seminomatous germ cell cancers exhibit a reversed trajectory, with parthenogenetic derivatives showing increased developmental potency along germline progression. Transformed late-stage hPGCs give rise to totipotent embryonic carcinoma cells, while transformed early-stage hPGCs yield less potent, primed-state hESC-like derivatives. In this transformed paradigm, prior germline entry is associated with an upregulation of developmental potency which is a distinct hallmark of non-seminomatous germ cell cancer transformation [1].

The inability of high-number hEGCLC_A4_L82 cells to form VP-MCC-like tumors in mice, compared to the successful VP-MCC-like tumor formation by high-number hiPSC_A4_L82 cells, provides functional evidence that hEGCLC_A4_L82 had failed to transition to the totipotent naïve hESC-like state required for VP-MCC-like tumor induction via the indirect pathway through hPGCLC_A4_L82 specification. This reduced developmental potency relative to its non-parthenogenetic counterpart aligns with the normal, rather than non-seminomatous germ cell cancer-transformed, hPGC parthenogenesis paradigm. This observation reflects MCPyV’s inability to functionally enhance parthenogenetic potency in hPGCLCs_A4_L82, in contrast to the developmental potency upregulation characteristic of non-seminomatous germ cell cancer transformation. MCPyV’s apparent incapacity to induce non-seminomatous germ cell cancer-like transformation indicates that hPGCLC_A4_L82 cells, upon viral transduction, cannot parthenogenetically acquire a totipotent embryonic carcinoma-like state. These findings are consistent with the global DNA methylation evidence supporting the requirement of a “late hPGC” state before direct MST to somatic VP-MCC-like tumors, without the involvement of a totipotent parthenogenetic intermediate. Furthermore, MCPyV’s inability to induce non-seminomatous germ cell cancer-like transformation also suggests that the teratoma components observed in hPGCLC_A4_L82-derived VP-MCC-like tumor (+) tumors have arisen directly from an in vivo parthenogenic derivative competent for teratoma formation, from hPGCLC_A4_L82 modeling early-hPGCs [35] as spontaneous germline developmental progression in vivo to a “late-hPGC” state would have resulted in a parthenogenic derivative incapable of teratoma formation [20].

### 4.2. Independent Tumorigenic Pathways for Teratoma vs. VP-MCC-like Tumor

Based on the VP-MCC-like tumor tumorigenic pathway supported by the obligatory extremely low global DNA methylation unique for “late-hPGC” state and the known intrinsic pluripotency-teratoma coupling with teratoma components exhibiting much higher global DNA methylation in VP-MCC-like tumor (+) tumors (Figure 4B), an independent tumorigenic pathway is favored for the teratoma components of VP-MCC-like tumor (+) tumors, involving multi-lineage somatic differentiation from a pluripotent precursor [20]. Failure of hEGCLC_A4_L82 to form VP-MCC-like tumors also provided functional evidence to oppose the involvement of parthenogenic intermediates in VP-MCC-like tumor tumorigenesis as discussed above. The independent tumorigenic pathways for the VP-MCC-like tumor component versus the teratoma component can provide the rationale for the co-presence of VP-MCC-like tumor and teratoma components in this VP-MCC mouse xenograft model, while no mixed VP-MCC and teratoma tumor has been reported in clinical settings. The answers may lie in the fact that the experimental mouse teratomas derived from human pluripotent stem cells are known to be polyclonal [20], while clinical cancer is a clonal disease originating from a single transformed cell including VP-MCC that is characterized by a clonal viral insertion site [21].

### 4.3. Potential Tumorigenic Functions by MCPyV

The inability of hPGCLC_A4 to form any tumor, teratoma or VP-MCC-like tumor, compared to the formation of both components by hPGCLC_A4_L82 implicates several potential tumorigenic functions of MCPyV. These include the following: (i) promoting survival or anti-apoptotic activity in hPGCLC_A4_L82 (contributing to both VP-MCC-like tumors and teratomas); (ii) enabling parthenogenetic reacquisition of pluripotency (teratomas only); (iii) facilitating progression from an early to late hPGC state (VP-MCC-like tumors only); and (iv) directly transforming late-hPGC-like cells into VP-MCC-like tumors (VP-MCC=like tumors only). Given MCPyV’s inability to induce non-seminomatous germ cell cancer-like transformation and the established teratoma-forming capacity of normal hEGCLCs, its role in teratoma formation is likely minimal. Rather, as extragonadal hPGCs are capable of spontaneous germline differentiation into late-hPGCs or beyond in non-gonadal environments before undergoing apoptosis [1], the most plausible MCPyV-driven mechanisms are enhanced survival and direct transformation of late-hPGC-like cells into VP-MCC-like tumors. One mechanism for MCPyV driving MST from late-hPGCs to VP-MCC-like tumors could be through its inhibition of p53 indirectly [23] as inactivating mutations of p53 have been associated with MST [1]. Further studies are warranted to elucidate the molecular mechanisms underlying MCPyV-mediated VP-MCC-like tumor formation.

### 4.4. Early Developmental vs. Carcinogenic Pathways

Our model supports a fundamental divergence between normal human development and VP-MCC carcinogenesis at the level of the Weismann barrier. In canonical early human development, pluripotent hESCs generate somatic tissues through orderly, multi-lineage differentiation. In contrast, VP-MCC appears to originate from a “unipotent” late-hPGC with known intrinsic germline programming to suppress somatic fate yet bearing latent or poised pluripotency [32], that aberrantly crosses the Weismann barrier into the somatic compartment, differentiating exclusively along a neuroendocrine lineage and undergoing developmental arrest. These findings suggest that VP-MCC may arise via an aberrant oncogenic process in which the germline directly gives rise to a somatic malignancy, which is akin to the emergence of a highly abnormal new life form (Figure 5). This MST model also supports key distinctions between somatic cancers, such as VP-MCC, and germ cell cancers exhibiting multilineage somatic differentiation, namely malignant teratomas. The latter more closely parallels normal human embryogenesis, requiring a totipotent embryonal carcinoma cell intermediate to traverse the Weismann Barrier and undergo multilineage somatic differentiation with aberrant developmental arrest. These embryonic carcinoma cells are thought to arise via parthenogenetic transformation of late-stage hPGCs in vivo, rather than from fertilization-derived zygotes. Future investigations are needed to determine whether this unifying germ cell theory-based paradigm applies more broadly to other somatic malignancies. For instance, in variant VN-MCCs characterized by heavy mutation burdens, a functionally equivalent “late-hPGC” state may be induced by somatic mutations, substituting for the native “late-hPGC” state observed in VP-MCCs (Figure 5).

### 4.5. Basic Science Evidence for Direct Germline-to-Soma Transitions

Direct germline-to-somatic differentiation does not normally occur, as germline programs actively suppress somatic fate. However, studies in model organisms such as *C. elegans* [67] demonstrate that disruption of specific epigenetic regulators in germ cells can unlock aberrant “permissive states” that enable direct germline-to-soma transitions into defined somatic lineages. Each permissive state allows only the corresponding lineage-determining transcription factors to act, thereby channeling differentiation toward a particular somatic fate. These findings provide proof-of-principle that a direct hPGC-to-somatic transition can occur without fertilization and without an intervening pluripotent embryonic stem cell stage. Extending this insight, distinct somatic cancer lineages may originate from disruption of specific epigenetic regulators that normally safeguard the germline state against soma-directed reprogramming. Systematic loss-of-function screens could map which epigenetic regulators protect hPGCs from transition into particular somatic lineages, thereby clarifying how aberrant germline-to-soma transitions seed somatic malignant trajectories.

### 4.6. Why Are hPGCs Potentially the Most Promising Candidates as the Cell of Origin for Somatic Cancer Stem Cells

Although hPGCs are not malignant, they are unusually preadapted for oncogenic conversion into *somatic* cancer stem cells compared with typical somatic cells. Several intrinsic properties make this state uniquely permissive:

- **High developmental plasticity:** PGCs occupy a transient, loosely specified state typified by expression of pluripotency-network factors such as OCT4 and NANOG, which lowers barriers to lineage switching and dedifferentiation—key cancer stem cell traits [68].
- **Global DNA hypomethylation nadir:** During migration and gonadal colonization, hPGCs reach an extreme hypomethylation minimum, enabling rapid transcriptional reprogramming but also heightening vulnerability to aberrant soma-like oncogenic programs [33,68].
- **Germline immortality programs:** Robust telomerase activity and DNA-repair systems support long-term self-renewal. When checkpoints are compromised, these same features can fuel immortalization typical of cancer stem cells [35,69,70].
- **piRNA–transposon axis:** hPGCs undergo global DNA demethylation and temporary relaxation of epigenetic repression permits transposon activity normally constrained by piRNA machinery; defective control creates genomic/epigenomic instability while preserving self-renewal [71,72,73].
- **Migratory and invasive phenotype:** Physiologic migration (via KIT/SCF, CXCR4/CXCL12, etc.) equips hPGCs with motility and niche-seeking behaviors readily co-opted by metastatic cancer cells [74,75].
- **Germline immune privilege-like traits:** Restricted HLA expression and germline-specific immunoregulatory pathways reduce immunogenicity, paralleling cancer stem cell immune evasion strategies [76,77].
- **Growth factor dependence:** hPGCs rely heavily on **BMP** and **KIT/KITLG signaling** for specification, survival, and migration [33,47]. This dependency renders them vulnerable to **oncogenic hijacking**, maintaining abnormal self-renewal outside normal niches [1].

Together, these properties define a developmental state that is epigenetically open, inherently self-renewing, migratory, and immune-evasive—an ensemble closely mirroring cancer stem cell biology. Thus, oncogenic hits (e.g., viral oncoproteins, pathway dysregulation) require fewer steps to convert hPGCs into somatic cancer–initiating cells.

We were initially surprised that MCPyV-transfected hEGCLC_A4_L82 cells did not form xenograft VP-MCC-like tumors, indicating the absence of a pluripotent/totipotent intermediary. However, considering the biological differences between totipotent/pluripotent embryonic stem cells (hESCs) and hPGCs makes this result plausible. In contrast to hPGCs, totipotent or pluripotent embryonic stem cells (arising only after fertilization) exhibit explicit pluripotency/totipotency that indiscriminately permits differentiation into all somatic lineages. In adult tumorigenic environments, this typically produces disorganized, multilineage differentiation, as observed in teratomas [1]. hPGCs, by contrast, embody *poised or latent pluripotency*, a state in which all somatic fates are actively suppressed but can be selectively unmasked upon loss of specific epigenetic safeguards, as suggested by earlier studies in *C. elegans* [67]. This selective release aligns with the biology of somatic carcinogenesis, where tumors usually adopt a single dominant lineage phenotype but may occasionally show focal differentiation into alternative lineages—including rare foci of extra-embryonic differentiation such as choriocarcinoma within otherwise somatic cancers [78].

Notably, prior work has shown that virus-positive Merkel cell carcinoma (VP-MCC) exhibits a seemingly paradoxical profile of epigenetic “youth” (DNA hypomethylation) without pluripotency [79]. This observation is consistent with the paradigm that hPGCs, unlike pluripotent embryonic stem cells, are primed for aberrant somatic fates without requiring explicit pluripotency, making them potentially the most promising developmental candidates for the cell state of origin in somatic carcinogenesis.

### 4.7. Therapeutic Implications from the VP-MCC Mouse Model

Global DNA hypomethylation is a universal hallmark of cancer [16], and its extent, like lineage plasticity, correlates with malignancy [16,80]. Our VP-MCC mouse model, which demonstrates direct transition from late hPGCs to somatic cancer cells, recapitulates this extreme hypomethylation and the associated highly plastic cell state. Notably, metastatic fronts in diverse cancers are enriched for hPGC-like cells [15], and even non-neuroendocrine carcinomas such as colorectal cancer can upregulate neuroendocrine programs at metastasis [81]. Taken together, these findings support a dynamic continuum between hPGC-like cancer stem cells and somatic cancer cells driven by aberrant plasticity. Because metastasis is the defining feature distinguishing malignant from benign tumors, therapeutic strategies that block this transition could profoundly reduce cancer progression.

Our tractable, genetically simple VP-MCC mouse model provides a powerful platform to interrogate this transition in vivo and to uncover additional drivers of VP-MCC and other small cell carcinomas. Existing therapeutic approaches, including epigenetic modulators, differentiation-inducing agents, and immunotherapies targeting state-specific antigens, could be rationally deployed, though their precise mechanisms of action in this context remain unclear. Importantly, differentiation therapy represents a whole-package strategy aimed at reprogramming the cancer cell state; however, its success has so far been limited largely to acute promyelocytic leukemia [82], reflecting how incomplete understanding of cancer’s developmental origins has hampered its broader application.

By contrast, the dominant therapeutic strategies today, such as immunotherapies to counter immune evasion and targeted therapies against specific mutations to blunt uncontrolled proliferation, are fundamentally piecemeal. They address isolated features of cancer stem cell biology without accounting for how these features integrate into the malignant cell state. This fragmented approach helps explain why such treatments often fail to deliver durable cures in most somatic cancers.

Our findings point toward the potential of a different therapeutic paradigm. By fundamentally understanding how somatic cancers originate from germline-like states, we can identify the vulnerable transition point, which is the re-specification of somatic cancer cells into hPGC-like states, and block it. Targeting the entire cancer stem cell state, rather than its individual traits, offers the prospect of halting metastasis at its root. BMP inhibitors are particularly compelling candidates, given the essential role of BMP signaling in normal hPGC specification.

Finally, by situating cancer within a defined developmental framework, our model provides a roadmap for identifying the specific epigenetic regulators that normally safeguard against germline-to-soma transitions. Insights from model organisms such as *C. elegans* [67] underscore the feasibility of such mapping, which could lead to mechanism-based interventions and, ultimately, the first truly curative strategies for somatic cancers.

#### Concluding Perspective

Late human primordial germ cells (hPGCs) represent potentially the most promising developmental template for understanding the somatic cancer stem cell state. They naturally possess the full repertoire of traits later seen in cancer stem cells: distant migratory and invasive capacity, immortality, global hypomethylation, immune evasion, and lineage plasticity which is a poised/latent totipotency program that occasionally surfaces in somatic cancers as foci of divergent or even extra-embryonic differentiation (e.g., foci of choriocarcinoma) [78]. Crucially, in normal development these traits are highly regulated and essential for germline continuity, whereas in carcinogenesis they become dysregulated and drive malignant transformation. From this developmental perspective, somatic carcinogenesis may be best understood as normal germline-derived biology gone awry.

This framing suggests that effective therapies should move beyond piecemeal strategies that target individual hallmarks (such as immune evasion or uncontrolled proliferation) and instead address the cancer stem cell state as an integrated whole. Blocking the re-specification of somatic cancer cells into hPGC-like states or interrupting the dysregulated germline-to-soma transition, would directly dismantle the cell-state engine that underlies metastasis and progression. Such whole-state targeting, grounded in developmental biology, may offer the most promising path toward durable cures in somatic malignancies.

### 4.8. Limitations

#### 4.8.1. Future Additional Functional Studies to Characterize Xenograft VP-MCC-like Tumors

Our model was orthotopic in mouse skin and faithfully reproduced key histologic and molecular features of VP-MCC, including characteristic malignant features such as tissue infiltration and lymphovascular invasion, reflecting functional characteristics of the VP-MCC-like tumors. Although no metastases were observed, this finding is consistent with the well-established observation that VP-MCC cell line-derived xenografts rarely metastasize [52], in stark contrast to the clinically aggressive human disease. This discrepancy may reflect fundamental differences between the murine and human in vivo environments. Further functional interrogation of metastatic potential, immune evasion, and therapeutic response is beyond the scope of this initial report; future work will address these endpoints in humanized or immune-reconstituted orthotopic models designed and powered to assess dissemination and treatment response.

#### 4.8.2. Future Detailed Molecular Studies to Further Validate Tumorigenic Pathways

Although spatial, epigenetic, and functional data collectively support the existence of a direct and an indirect pathway from hPGC-like cells to MCC via a late-hPGC-like state, no lineage tracing, time-course analysis, or single-cell sequencing were performed to further support the progression. Further validation of these tumorigenic pathways, as well as elucidation of the underlying molecular mechanisms, could be achieved using such future studies.

#### 4.8.3. Future Mouse Study Design to Validate Reproducibility

This mouse model may benefit from further validation of reproducibility by using additional sets of primeval cell lines from hESCs rather than iPSCs. Primeval stem cells from both male and female donors are also desired, because of the male dominance in Merkel cell carcinoma patients.

## 5. Conclusions

In summary, we established an in vivo MST mouse model demonstrating MCPyV-driven transformation of hPGCLCs into somatic VP-MCC–like tumors. Global DNA methylation analyses identified a “late-hPGC” state as a necessary developmental precursor for direct MST to VP-MCC–like tumors. Spatial, epigenetic, and functional data collectively support the existence of independent tumorigenic trajectories for the VP-MCC–like and teratoma components within the same tumor, thereby opposing a parthenogenetic pluripotent or totipotent intermediate in VP-MCC tumorigenesis. This model directly establishes a developmental link between late hPGCs and a somatic cancer, supporting the view that germ cell cancers and somatic cancers may share the same cell (or cell-state) of origin. Finally, the genetic simplicity and experimental tractability of this VP-MCC-based MST system provide a robust platform for mechanistic dissection of VP-MCC pathogenesis and may yield critical insights into the biology of VN-MCC and other treatment-refractory high-grade neuroendocrine carcinomas. By defining a developmental origin for VP-MCC, this model advances a developmental biology-driven framework for somatic cancer research beyond mutation-centric and soma-centric paradigms.

## Figures and Tables

**Table 1:** Teratoma assay-like MST mouse xenograft model design: total 18 NSG adult male mice divided to 6 groups of 3 mice each. For Groups #1 and #2, only right flank side injection was performed per mouse (*n* = 3 total injections per group). For Groups #3–#6, bilateral flank injections were performed per mouse (*n* = 6 total injections per group). The experimental unit in this study is per injection.

Primeval stem cell type	hPGCLC		hiPSC		hEGCLC	
Cell line	hPGCLC_A4	hPGCLC_A4_L82	hiPSC_A4_L82		hEGCLC_A4_L82	
**Group** (cell number/injection)	**Group#1** (3 × 10^5^/injection)	**Group#2** (3 × 10^5^/injection)	**Group#3** (1 × 10^6^/injection)	**Group#4** (2 × 10^7^/injection)	**Group#5** (1 × 10^6^/injection)	**Group#6** (2 × 10^7^/injection)
# of Mice	3	3	3	3	3	3
Injected flank side	right only	right only	right and left	right and left	right and left	right and left
***n*** (# of injections/group)	***n* = 3**	***n* = 3**	***n* = 6**	***n* = 6**	***n* = 6**	***n* = 6**

**Table 2:** Summary of mouse tumor formation from subcutaneously injected human primeval stem cells: VMLT (VP-MCC-like tumors). Three possible outcomes from an injection: (1) formation of VMLT (+) tumor with both VMLT and teratoma; (2) formation of VMLT (−) tumor with only teratoma and no VMLT; (3) no tumor formation.

Primeval stem cell type		hPGCLC		hiPSC		hEGCLC	
Cell line		hPGCLC_A4	hPGCLC_A4_L82	hiPSC_A4_L82		hEGCLC_A4_L82	
**Group** (cell number/injection)		**Group #1** (3 × 10^5^/injection)	**Group #2** (3 × 10^5^/injection)	**Group #3** (1 × 10^6^/injection)	**Group #4** (2 × 10^7^/injection)	**Group #5** (1 × 10^6^/injection)	**Group #6** (2 × 10^7^ /injection)
# of Mice		3	3	3	3	3	3
Injected flank side		right only	right only	right and left	right and left	right and left	right and left
**n** (total # of injections/group)		***n* = 3**	***n* = 3**	***n* = 6**	***n* = 6**	***n* = 6**	***n* = 6**
**VMLT (+)** **Tumor**	# of mice	0	2	0	3	0	0
	# of injections	***n* = 0**	***n* = 2**	***n* = 0**	***n* = 6**	***n* = 0**	***n* = 0**
**VMLT (−)** **Tumor**	# of mice	0	0	3	0	3	3
	# of injections	***n* = 0**	***n* = 0**	***n* = 6**	***n* = 0**	***n* = 6**	***n* = 6**
**NO** **Tumor**	# of mice	3	1	0	0	0	0
	# of injections	***n* = 3**	***n* = 1**	***n* = 0**	***n* = 0**	***n* = 0**	***n* = 0**

## Data Availability

All data are available in the main text or the Supplementary Materials.

## Supplementary Materials

The following supporting information can be downloaded at: https://www.mdpi.com/article/10.3390/cancers17172800/s1, Figure S1: Summary of prior study findings on two global DNA methylation (GDM) nadirs following two waves of global DNA demethylation during early embryogenesis and gametogenesis of human development. Figure S2: Normal karyotype (46, XY) for all four primeval stem cell lines injected for the mouse study. Figure S3: Cell cultures of four primeval stem cell lines injected into NSG mice. Figure S4: Gross appearance and representative histology of additional mouse VP-MCC-like tumors (VMLTs) derived from human primeval stem cells. Figure S5: Gross appearance of mouse tumors composed of teratoma only. Figure S6: Bilateral teratomas formed after hiPSC_A4_L82 injection. Figure S7: Bilateral teratomas formed after hEGCLC_A4_L82 injection. Figure S8: Representative sections of T#85.4.N1 and T#85.4.N2. Figure S9: Sections of VMLTs of T#84.5R and T#84.5L. Figure S10: Tumor T#85.3. Figure S11: Representative sections of VP-MCC-like tumor in superficial mouse skin of T#85.3 with extensive necrosis and ulceration. Figure S12: Representative sections of VP-MCC-like tumors of T#85.1L and T#85.1R. Figure S13: Representative sections of VP-MCC-like tumors of T#85.2L and T#85.2R. Figure S14: Cytology smears of mouse xenograft VP-MCC-like tumors. Figure S15: VP-MCC-like tumors of T#85.3 and T#85.4 exhibit malignant histological features. Figure S16: Additional IHC studies of T#85.4. Figure S17: IHC studies of Ki67 and MCPyV LT in VP-MCC-like tumors. Figure S18: Immunohistochemical stains of T#85.3. Figure S19: Immunohistochemical stains of T#84.5R. Figure S20: Immunohistochemical stains of T#85.1L. Figure S21: Immunohistochemical stains of T#85.2L. Figure S22: Immunohistochemical stains of VP-MCC and VN-MCC cell lines. Figure S23: Additional 5mC IHC studies. Figure S24: Rim of VP-MCC-like tumor cells infiltrate deep tumor capsule covering teratoma component with a differentiation gradient in T#85.3 tumor. Figure S25: Promega OncoMate MSI Analysis System was used for authentication of injected primeval stem cell lines and derived VP-MCC-like tumor (+) tumors. Table S1: Mouse Xenograft Study Summary. Table S2: RT-qPCR results and statistical analysis of hPGCLC_A4_L82, hiPSC_A4_L82, seven VMLTs, VP-MCC cell lines, and VN-MCC cell lines. Table S3: Summary of IHC study results of VMLTs. Table S4: Global 5mC levels using flow cytometry. Median fluorescence intensity (MFI) and standard error of the mean (SEM) were calculated using FlowJo_v10software and downstream statistical analysis. Table S5: Primers for RT-qPCR. Table S6. Primary antibodies used for IHC studies.

## Abbreviations

hiPSC	human-induced pluripotent stem cell
VN-MCC	Virus-negative MCC
hEGCLC	hEGC-like cells
EC	embryonal carcinoma
ECC	embryonic carcinoma cell
hEGC	human embryonic germ cell
hEGCLC	hEGC-like cell
EGGCT	extragonadal germ cell tumor
hESC	human embryonic stem cell
HGNEC	high grade neuroendocrine carcinoma
GCC	germ cell cancer
GCT	germ cell tumor
GDM	global DNA methylation
ICM	inner cell mass
IHC	immunohistochemical
iPSC	induced pluripotent stem cells
hiPSC	human iPSC
LTC	long-term-culture
MCC	Merkel cell carcinoma
MCPyV	Merkel cell polyomavirus
MST	malignant somatic transformation
NSGCC	non-seminomatous germ cell cancer
PGC	primordial germ cell
hPGC	human primordial germ cell
hPGCLC	hPGC-like cell
hPSC	human pluripotent stem cell
SCF	stem cell factor
SCNC	small cell neuroendocrine carcinoma
SDF-1	stromal cell-derived factor 1
VN	virus-negative
VP-MCC	virus-positive Merkel cell carcinoma
VMLT	VP-MCC like tumor
VN-vMCC	virus-negative variant MCC
WB	Weismann barrier
