# Supplementary material for: A Polyomavirus-Positive Merkel Cell Carcinoma Mouse Model Supports a Unified Origin for Somatic and Germ Cell Cancers
Source: Cancers (Basel). 2025 Aug 27;17(17):2800. doi: 10.3390/cancers17172800 (PMC12427375; doi:10.3390/cancers17172800)
Supplement: Supplementary file 1 [file cancers-17-02800-s001.zip › Supplemental Information Except Table S2 & Table S4.pdf]

# Supplementary Information

## Virus-positive Merkel cell carcinoma model supports a unified cancer origin

**Authors:** Wendy Yang[1]*, Sara Contente[1], Sarah Rahman[1]
*Corresponding author. Email: wendy.yang@usuhs.edu

### The supplemental information includes:

Figures. S1 to S24
Tables S1, S3, S4, S5, S6

### Other Supplementary information for this manuscript include the following:

Table S2 (in Excel file)
Table S4 (In Excel file)

## Figures S1-S21

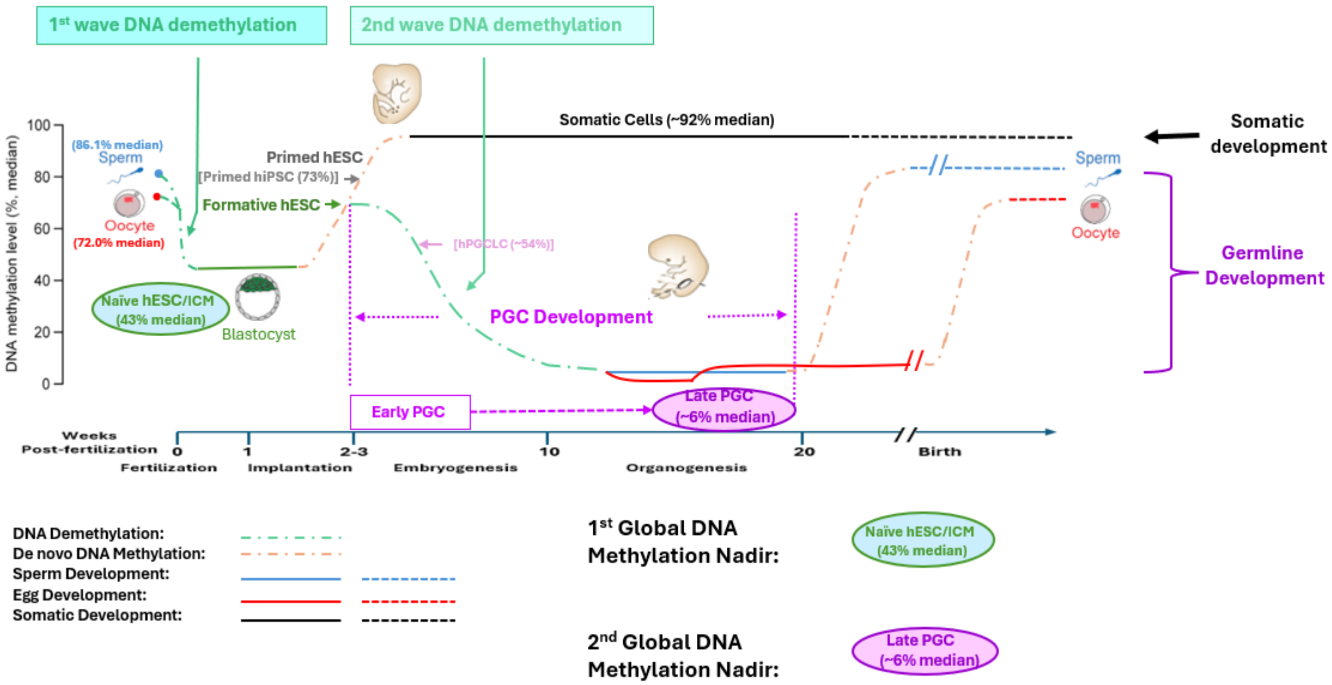


**Figure S1. Summary of prior study findings on two global DNA methylation (GDM) nadirs following two waves of global DNA demethylation during early embryogenesis and gametogenesis of human development** [33, 34, 35]. The 1st wave of global DNA demethylation during the 1st week of embryogenesis starts at the methylation level of the zygote after fertilization and decreases to the lowest level of naive hESCs/Inner Cell Mass (ICM) at the early blastocyst state, the 1st GDM nadir (~43% median). Subsequently, de novo methylation starts and reaches the formative hESC state which will bifurcate to either the somatic fate or the germline fate. The somatic fate trajectory entails continued de novo methylation from the formative hESC state to the primed hESC state (mimicked by primed hiPSC ~73%) followed by somatic lineage differentiation until they develop into mature somatic cells with stable and high methylation levels (~92% median). The germline fate trajectory entails germline-specific 2nd wave global DNA demethylation which starts when hPGCs are specified from formative hESCs with hPGCs becoming progressively demethylated along the hPGC developmental trajectory from early hPGCs (mimicked by hPGCLC ~54%) to late hPGCs, the 2nd GDM nadir (~6% median). Subsequently, de novo methylation starts along the gonadal gametogenesis trajectory with GDM of developing germ cells gradually increasing until fertilization (sperm ~86.1% and oocyte ~72.0%). Adapted and used with permission from Wen et al., 2019.

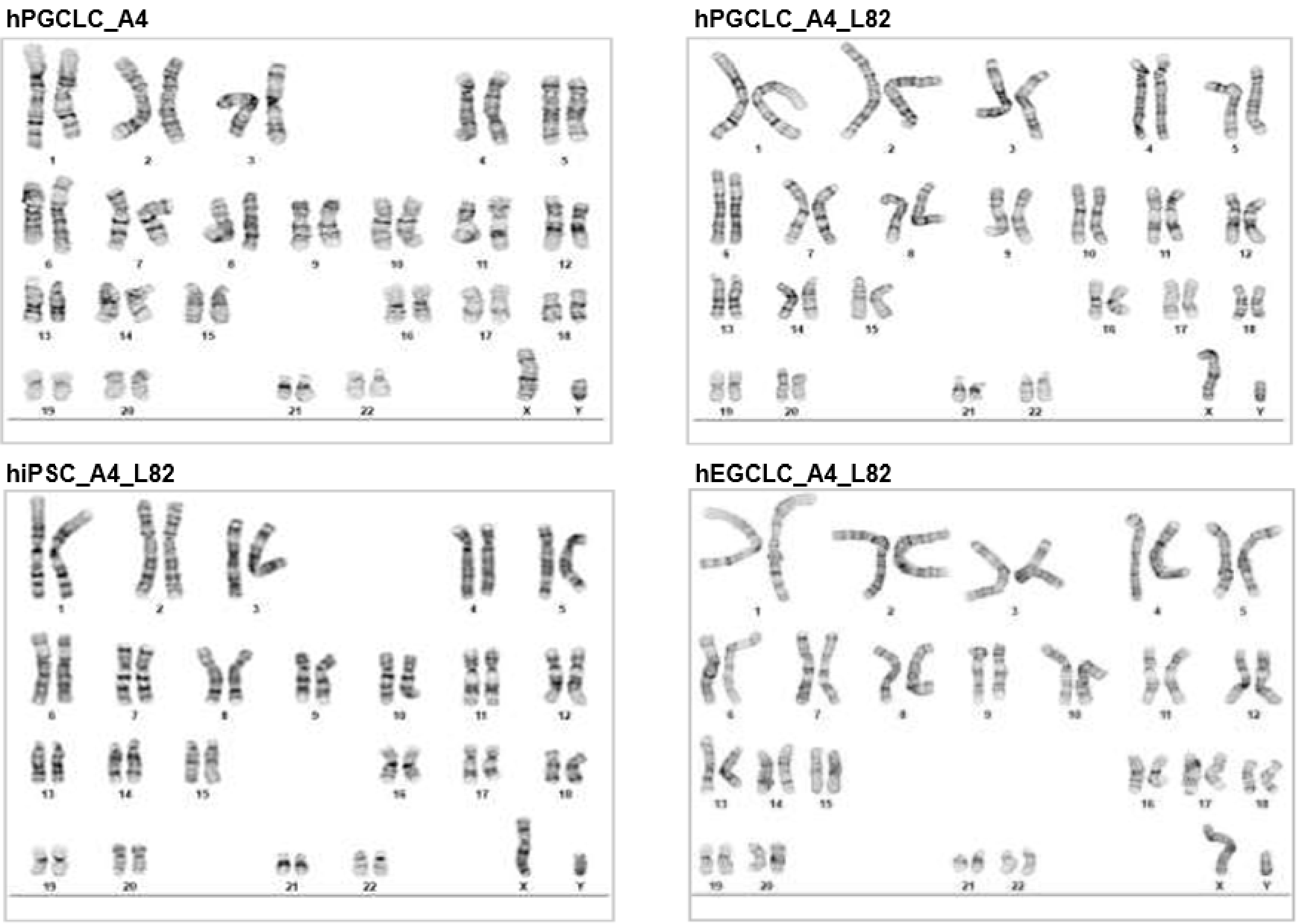


**Figure S2. Normal karyotype (46, XY) for all 4 primeval stem cell lines injected for the mouse study.**

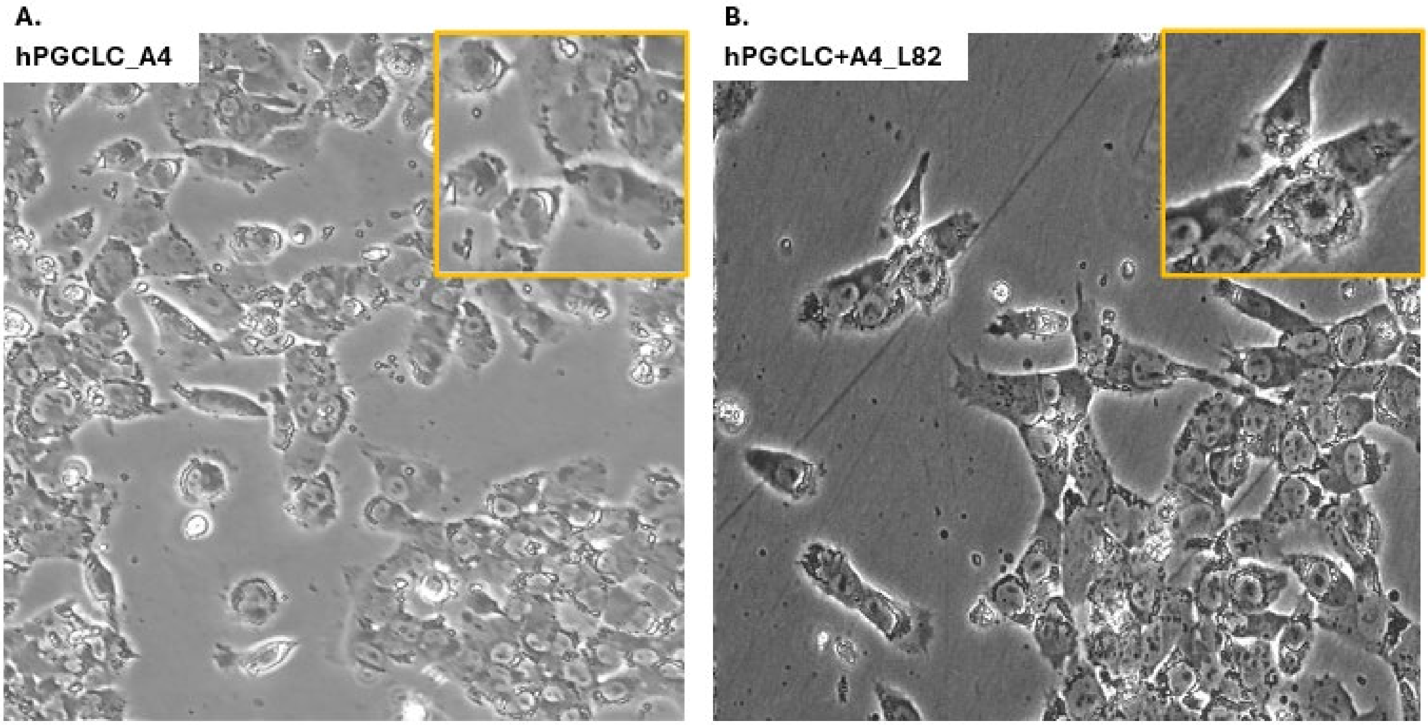

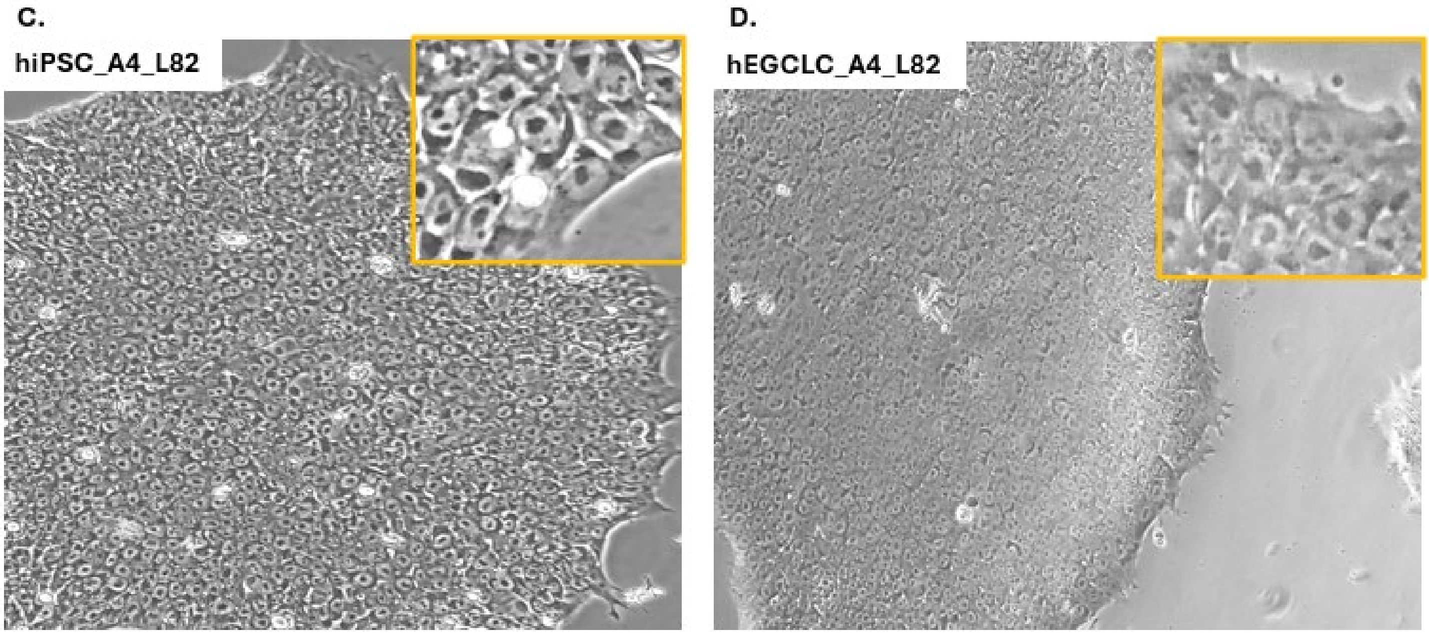


**Figure S3. Cell cultures of four primeval stem cell lines injected into NSG mice.** (**A**) hPGCLC_A4 (**B**) hPGCLC_A4_L82 (**C**) hiPSC_A4_L82 (**D**) hEGCLC_A4_L82

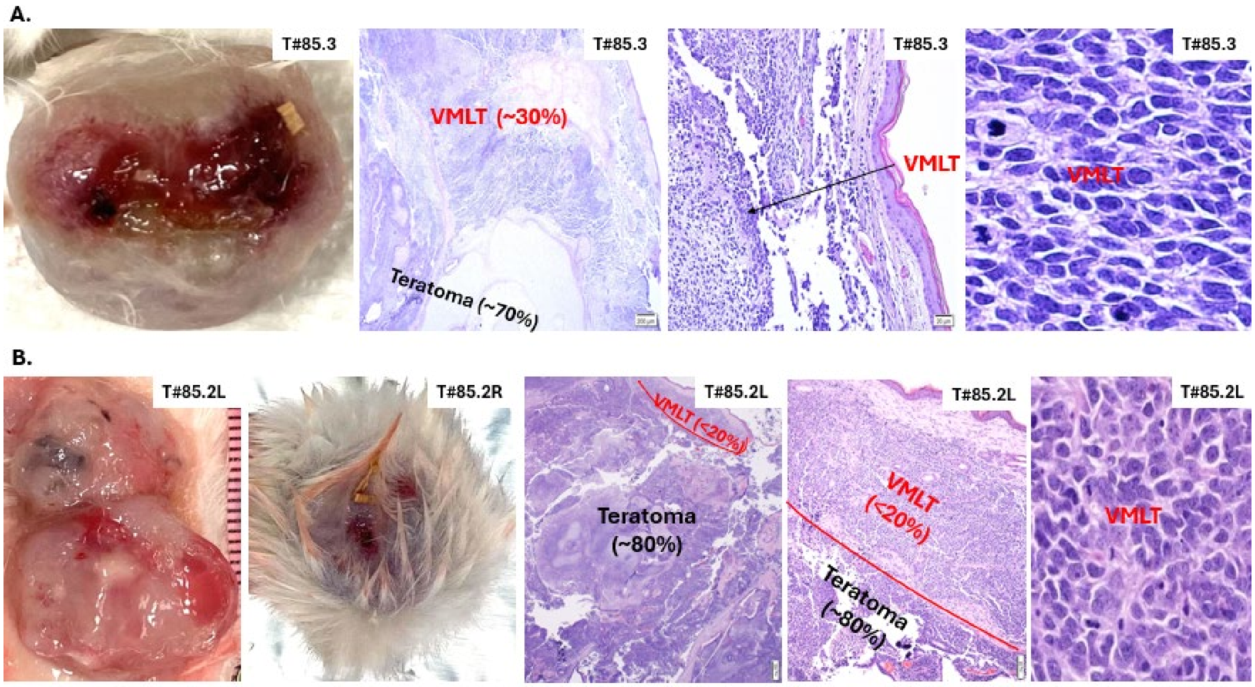

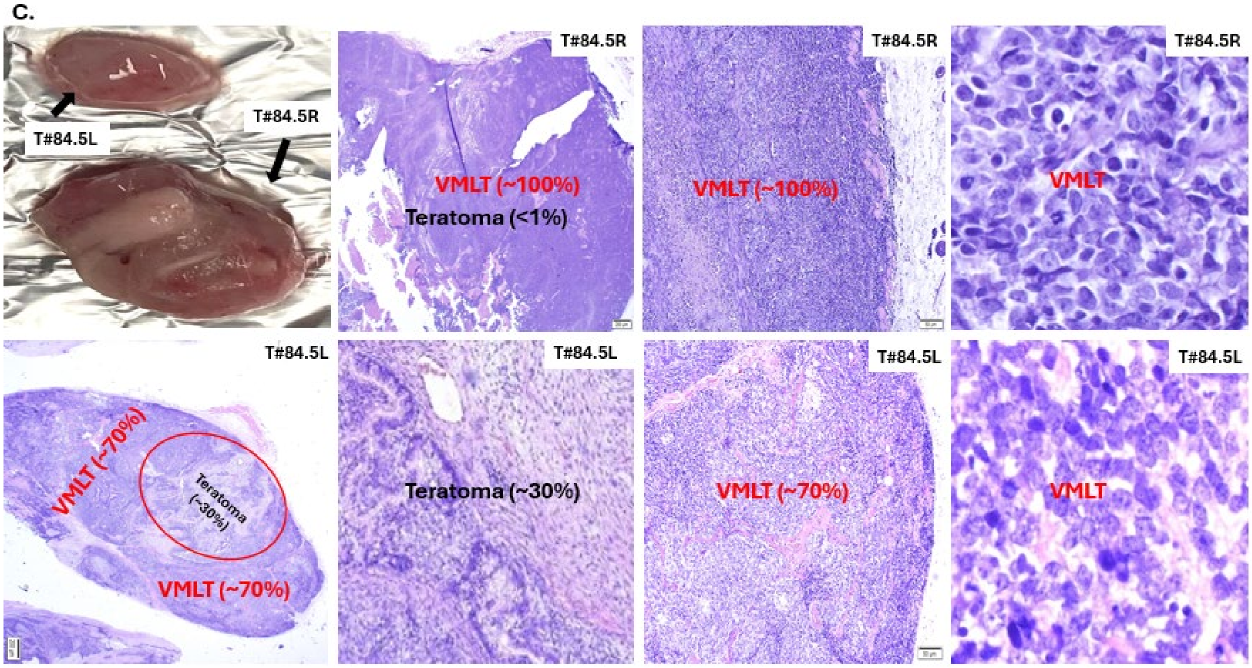


**Figure S4. Gross appearance and representative histology of additional mouse VP-MCC-like tumors (VMLTs) derived from human primeval stem cell**s: Injected with hPGCLC_A4_L82 (~ 3 × $10^5$ cells): (A) Mouse #85.3. Injected with hiPSC_A4_L82 (~ 2 × $10^7$ cells): (B) Mouse # 85.2 and (C) Mouse #84.5.

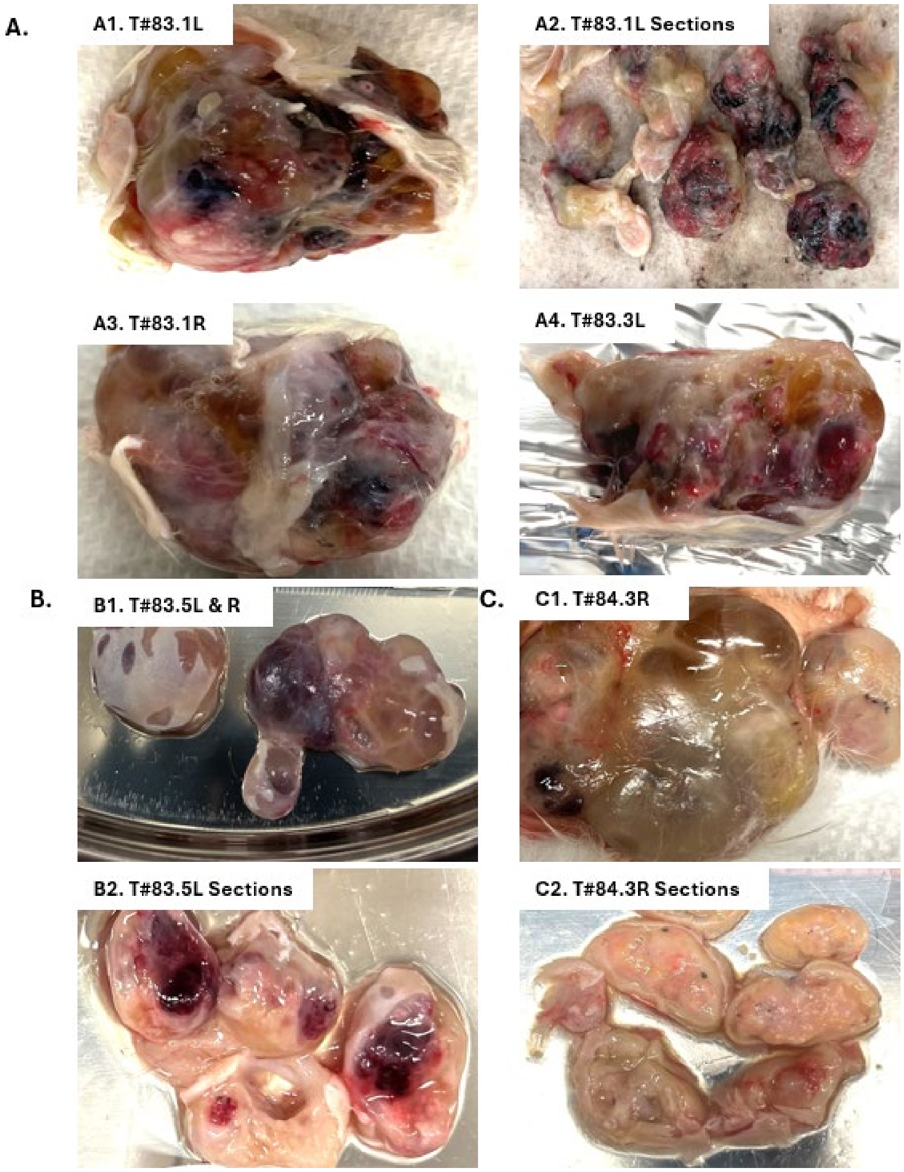


**Figure S5. Gross appearance of mouse tumors composed of teratoma only.** Tumors induced by injection of (A) conventional number of hiPSC-A4_L82 cells: T#83.1L, T#83.1R and T#83.3L (B) conventional number of hEGCLC_A4_L82 cells: T#83.5L (C) very high number of hEGCLC_A4_L82 cells: T#84.3L

**A. T#83.1L and T#83.1R (Teratoma, ~40% necrosis & cystic)**

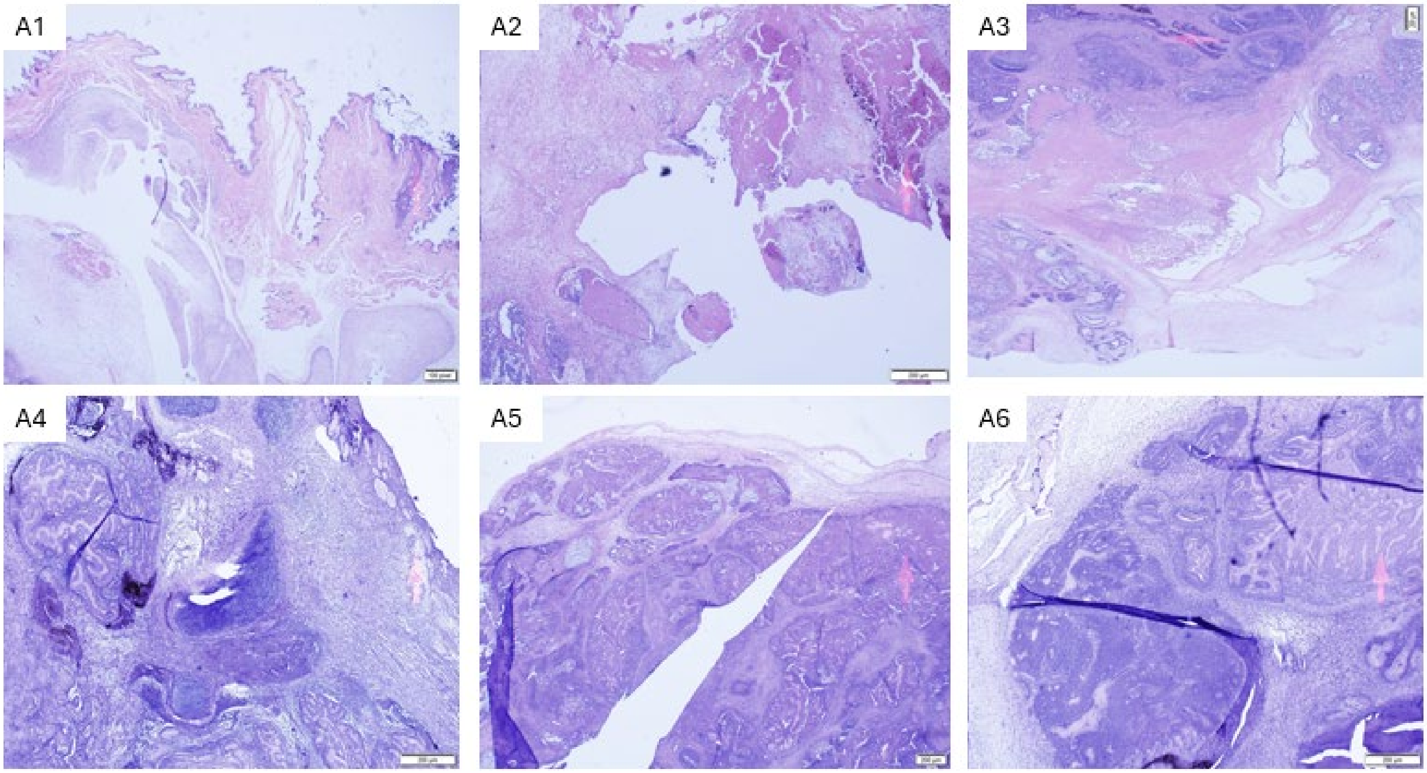


**B. T#83.2L and T#83.2R (Teratoma, ~30% necrosis & cystic)**

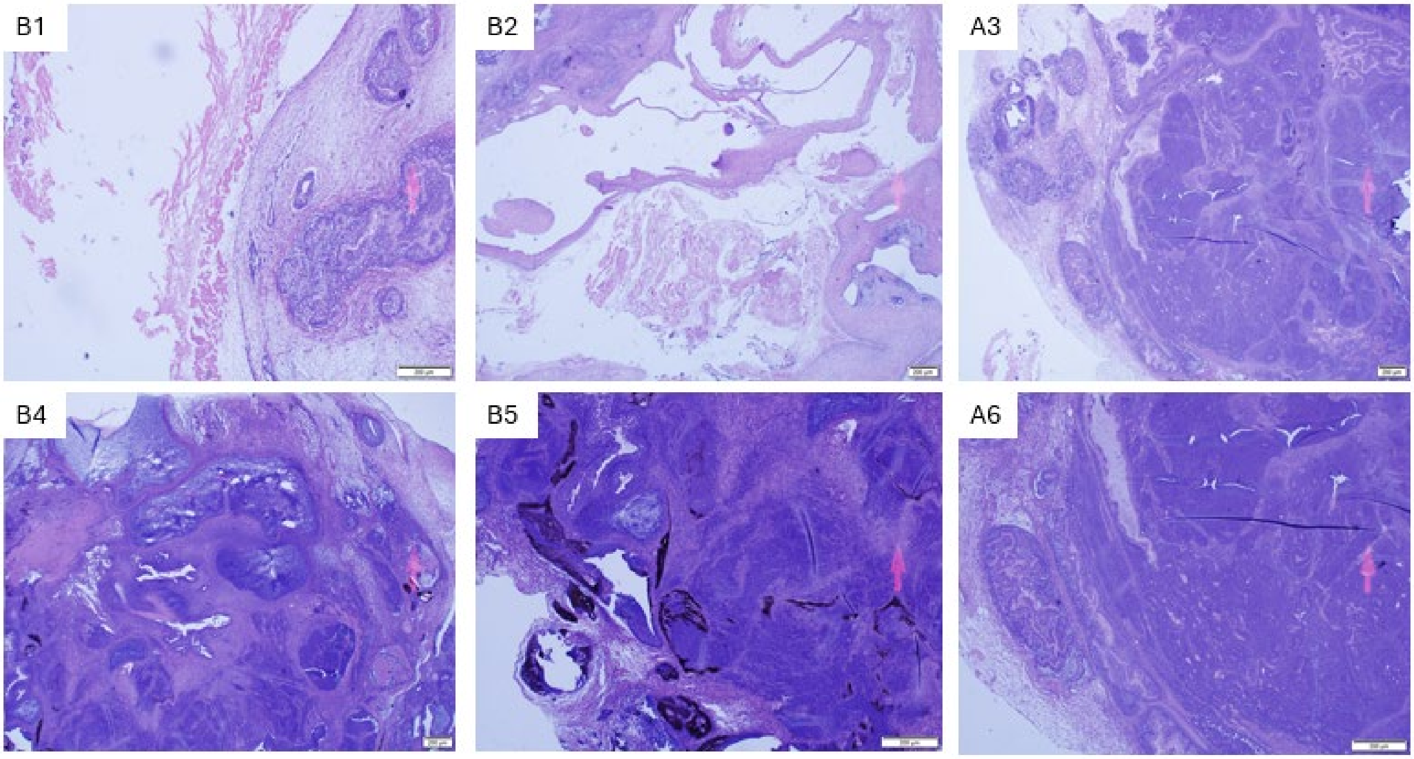

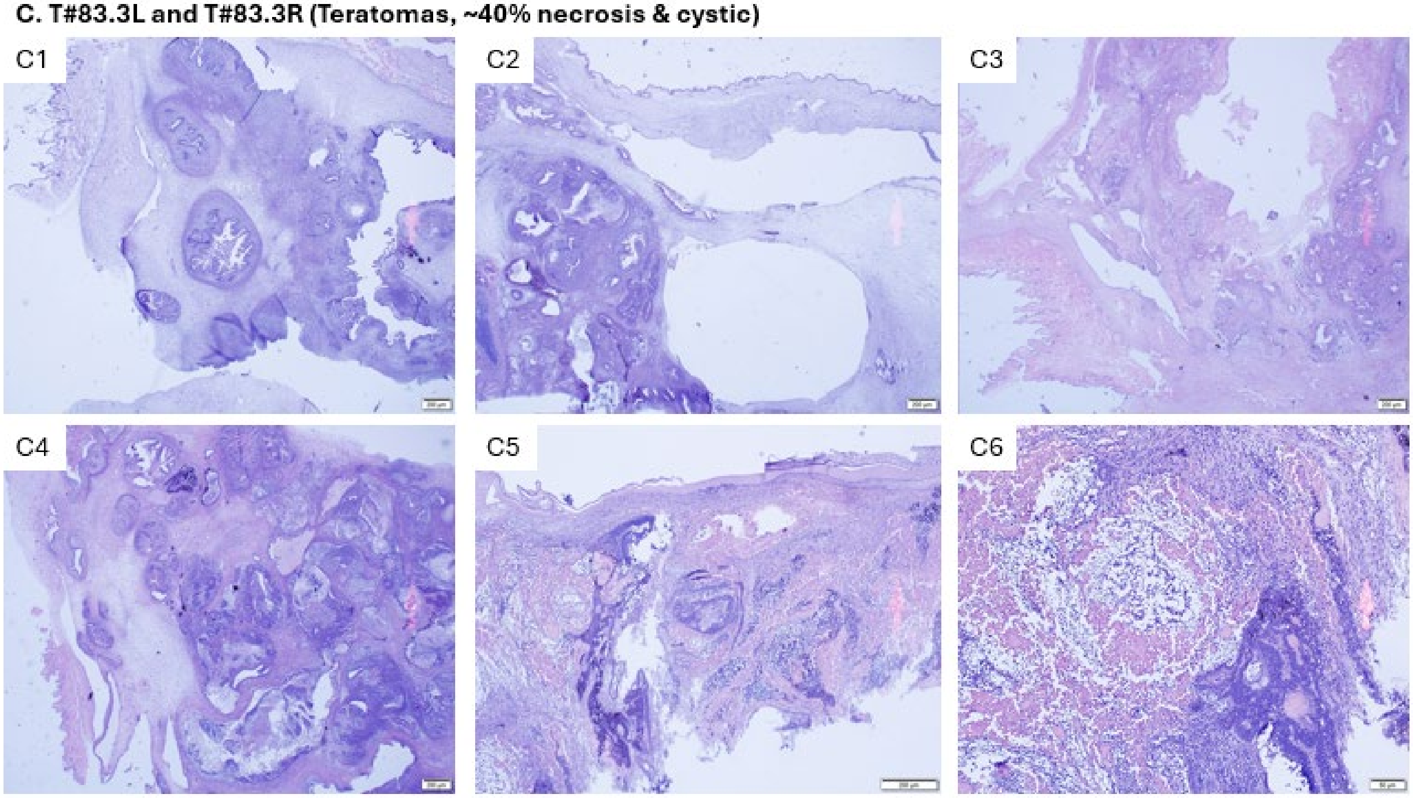


**Figure S6. Bilateral teratomas formed after hiPSC_A4_L82 injection.** Mice were injected with conventional numbers ($1 \times 10^{6}$ cells) of hiPSC_A4_L82. (A) mouse #83.1 (B) mouse #83.2 (C) mouse #83.3. Frequent foci of cyst formation and necrosis are noted. Although some areas of immature neuroectodermal tissues were noted, no large infiltrating sheets of small blue cells to suggest malignant somatic transformation to MCC-like tumors were identified. The teratomas exhibited no malignant histological features with round and lobulated rather than infiltrating borders.

A. T#83.4L and T#83.4R (Teratoma, ~40% necrosis and cystic)

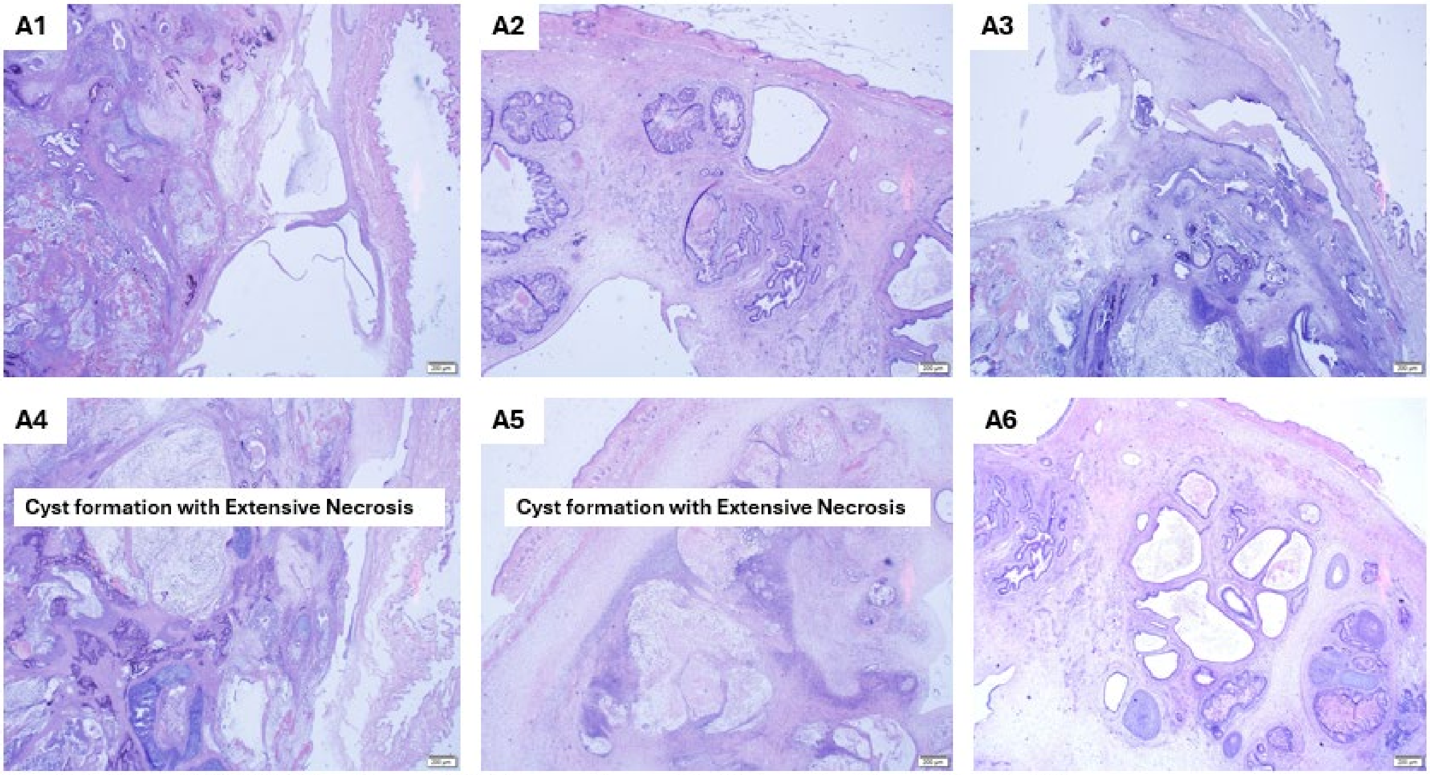


B. T#83.5L and T#83.5R (Teratoma, ~30% necrosis, cystic & abundant hematopoiesis)

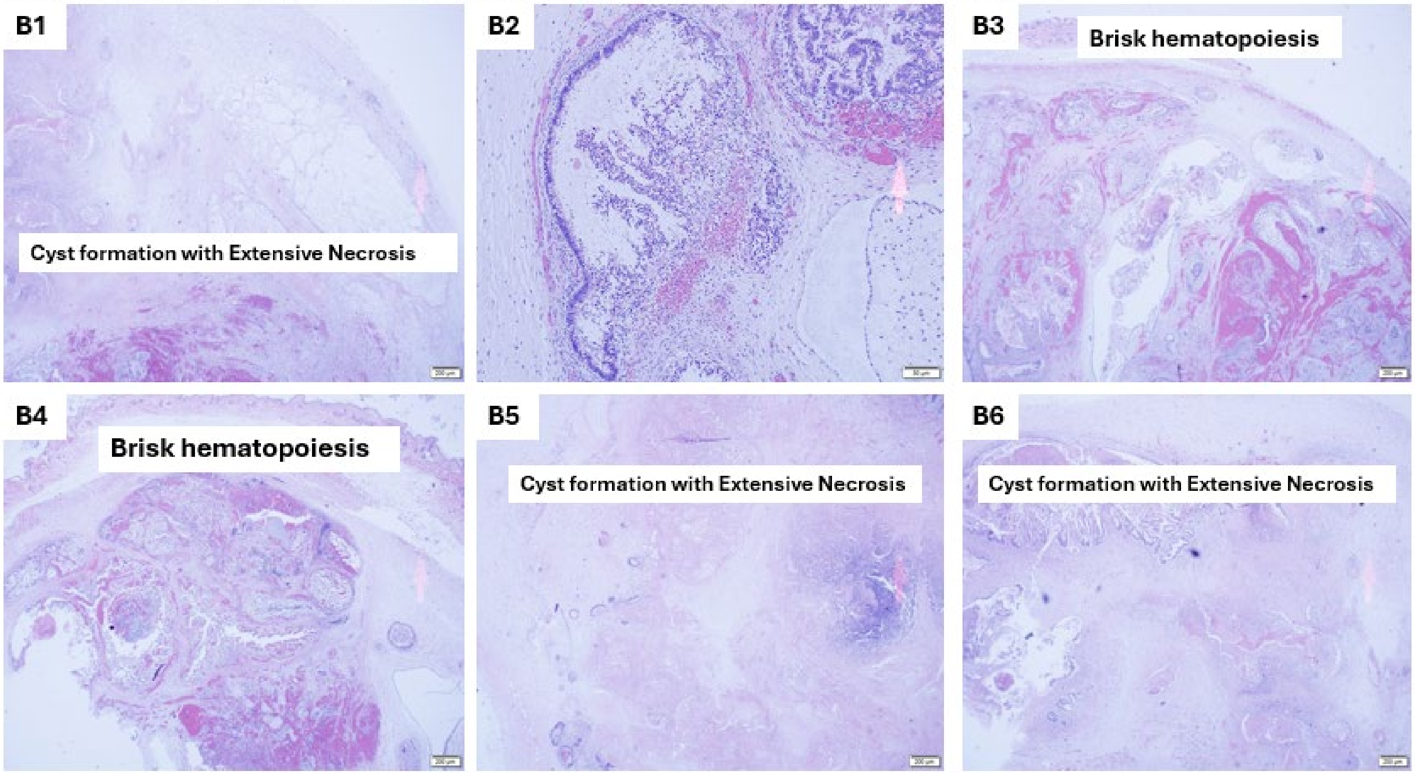

C. T#84.1L and T#84.1R (Teratomas, 20% necrosis and cystic)

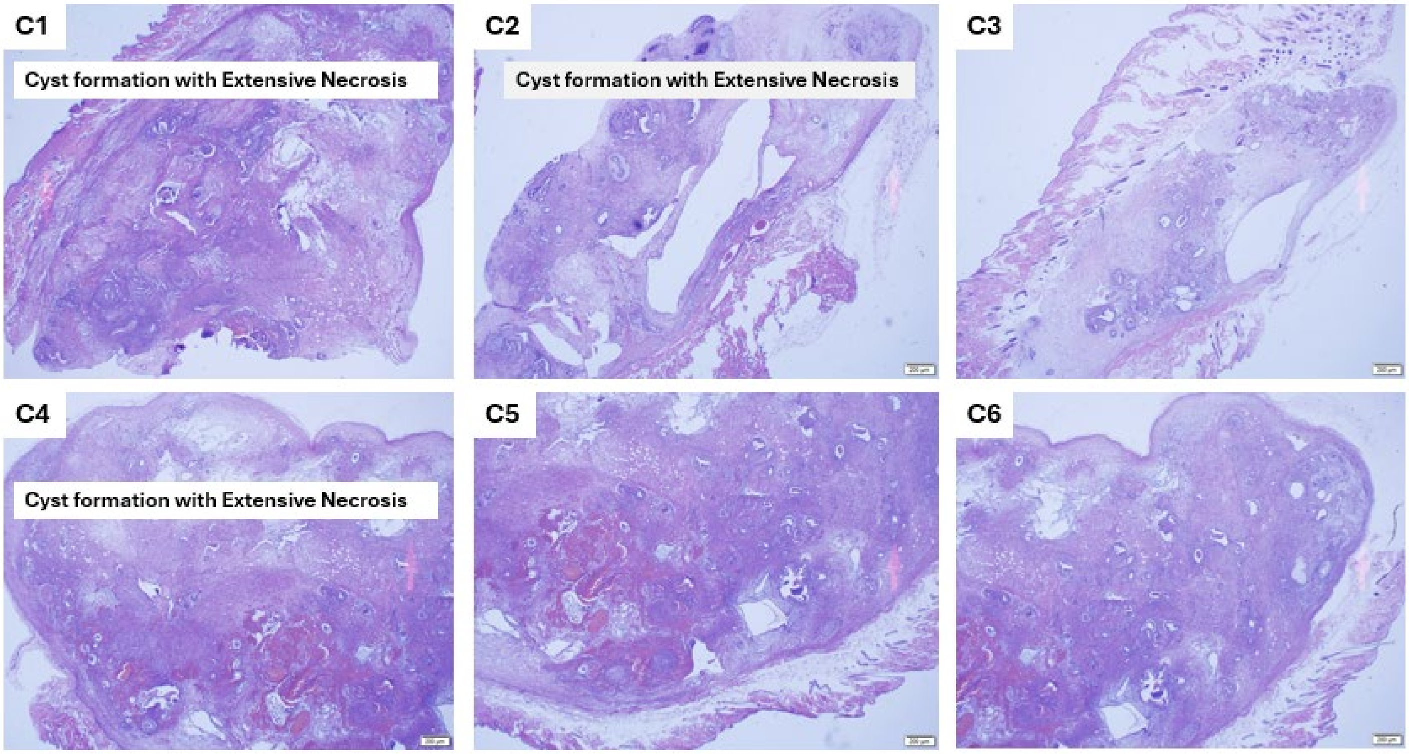


D. T#84.2L and T#84.2R (Teratomas, ~20% necrosis and cystic)

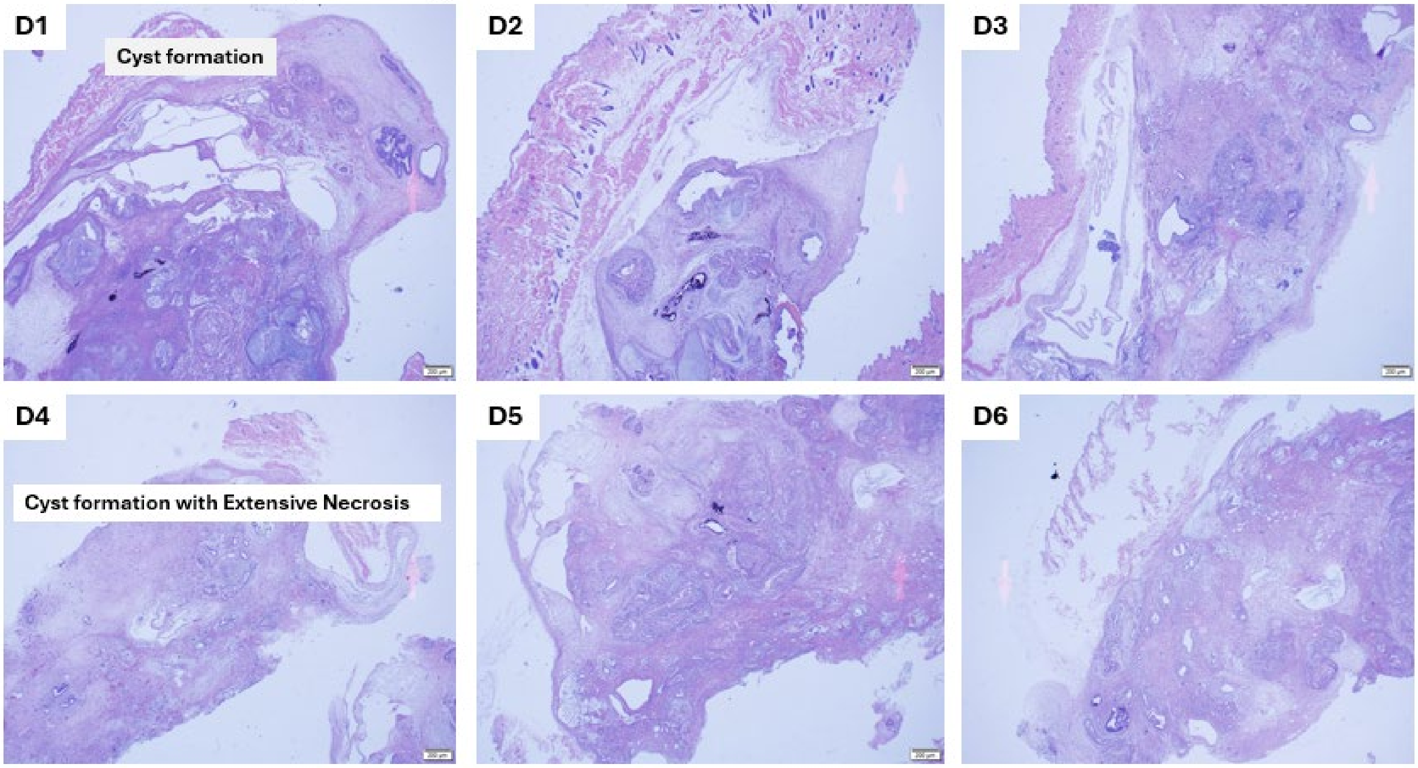

**E. T#84.3L and T#84.3R (Teratomas, ~60% necrosis and cystic)**

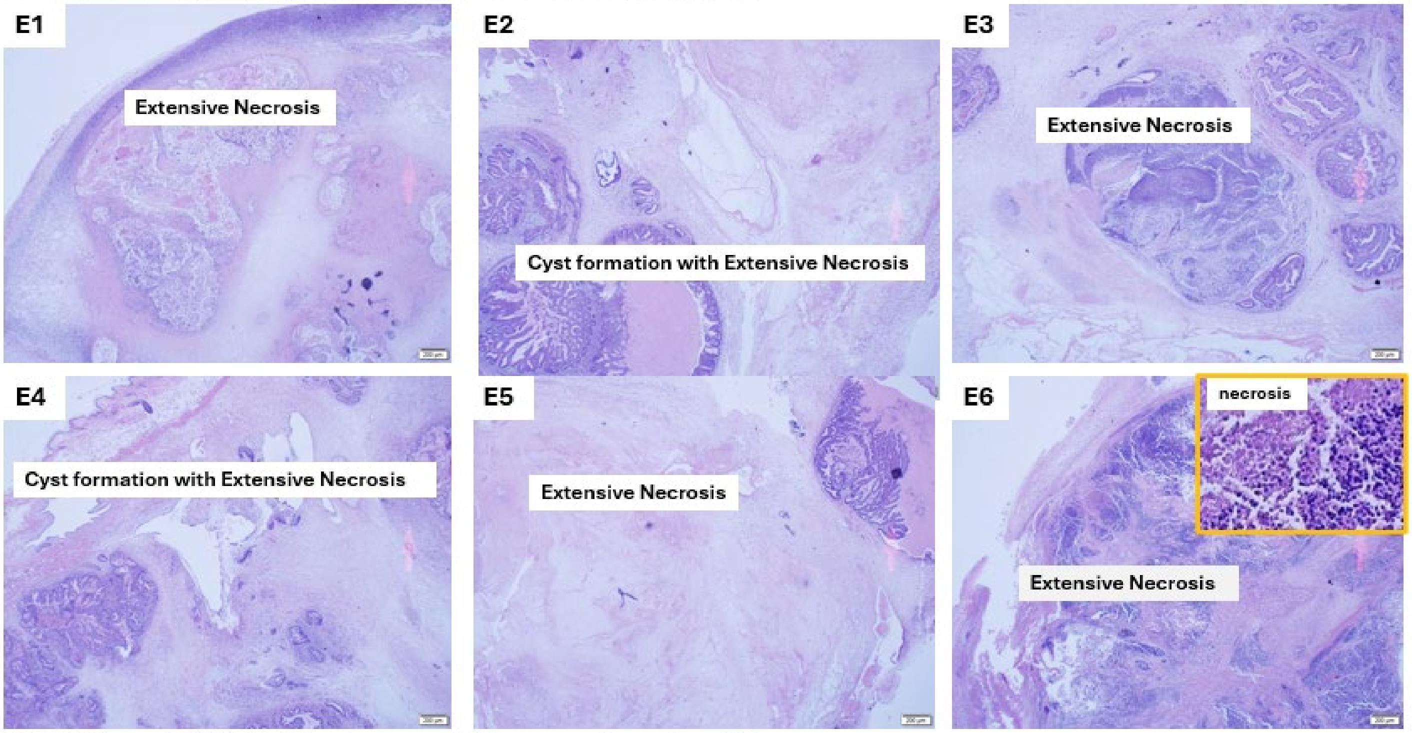


**F. T#84.4L and T#84.4R (Teratomas, ~30% necrosis and cystic)**

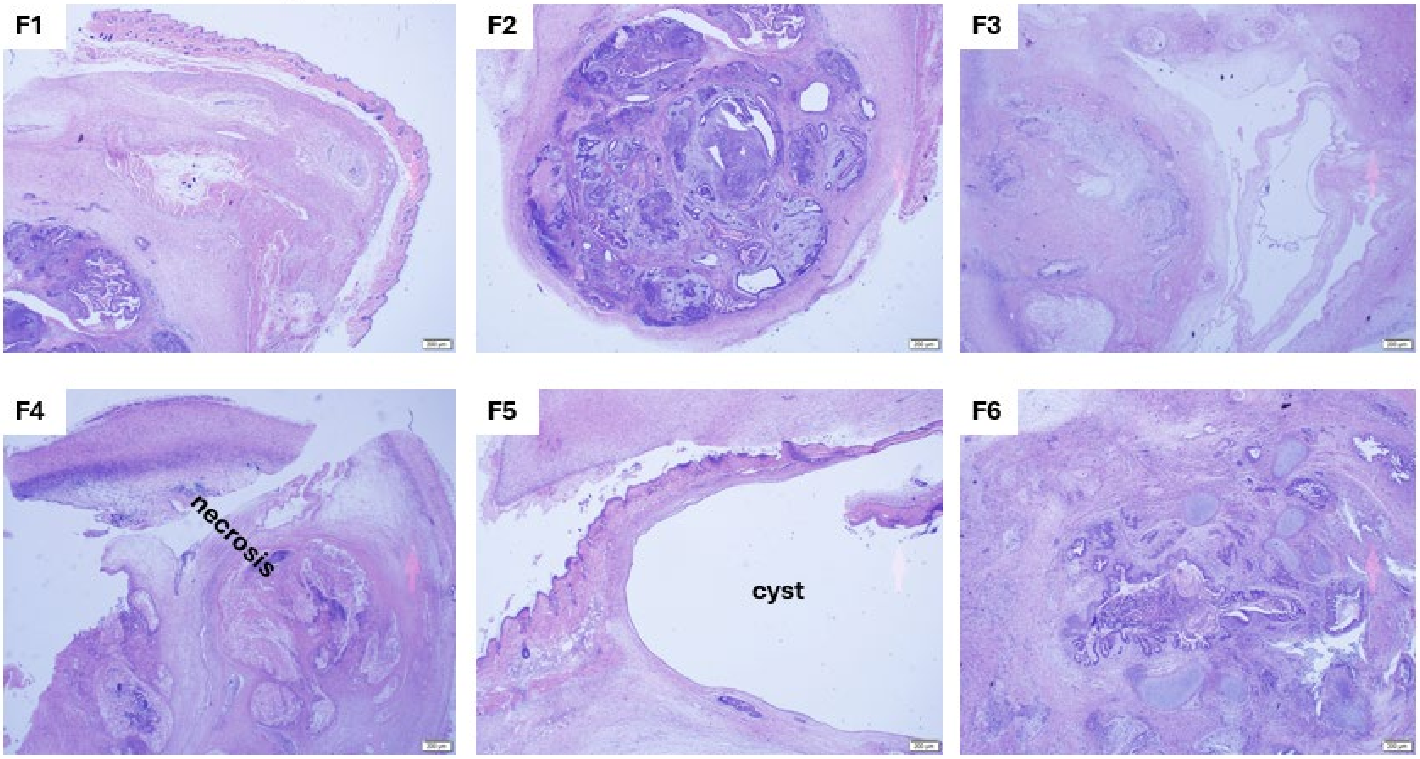


**Figure S7. Bilateral teratomas formed after hEGCLC_A4_L82 injection.** Mice were injected with conventional numbers ($1\times10^6$ cells) of hEGCLC_A4_L82 **(A)** mouse #83.4 **(B)** mouse #83.5 **(C)** mouse #84.1, or with very high numbers ($2\times10^7$ cells) of hEGCLC_A4_L82 **(D)** mouse #84.2 **(E)** mouse #84.3 **(F)** mouse #84.4. All hEGCLC_A4_L82 derived tumors, despite the difference in numbers of cells injected, showed similar histology and resembled the teratomas derived from injections with conventional numbers of hiPSC_A4_L82, with frequent cyst formation, foci of necrosis and no evidence of malignant somatic transformation to MCC-like tumors. Brisk hematopoiesis was noted in bilateral teratomas of mouse #83.5.

A.

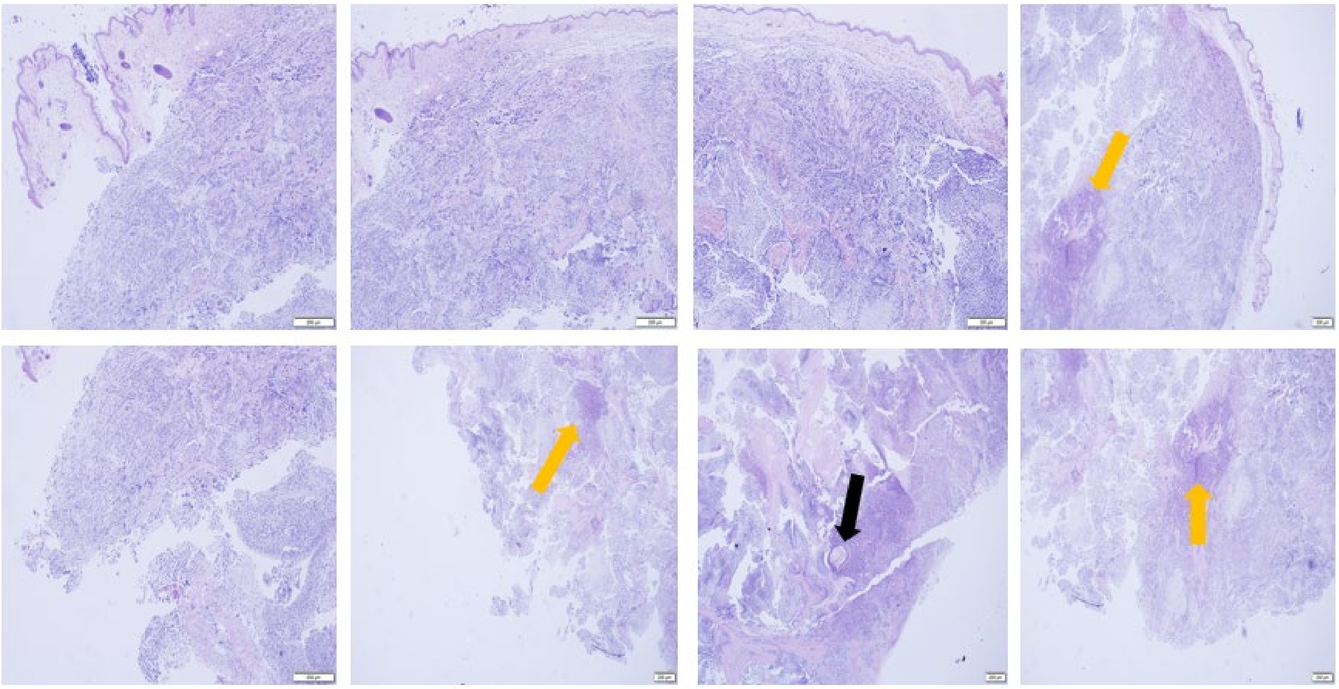

B.

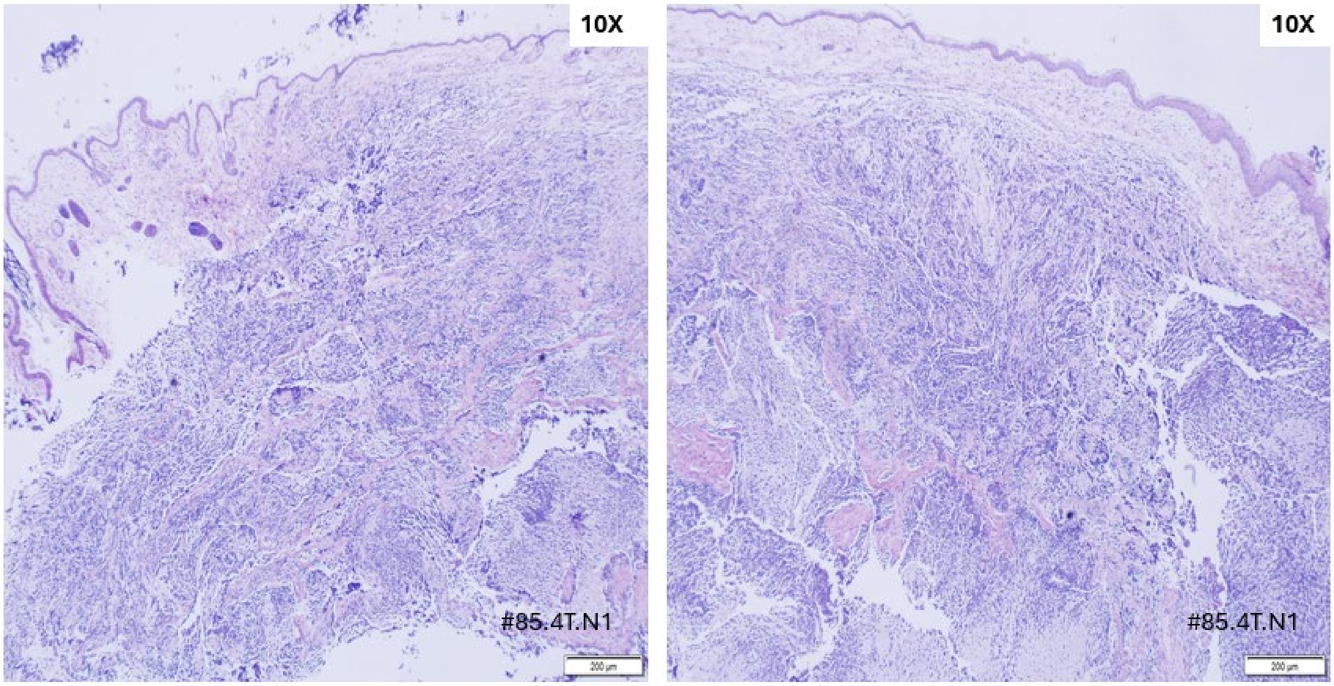

C.

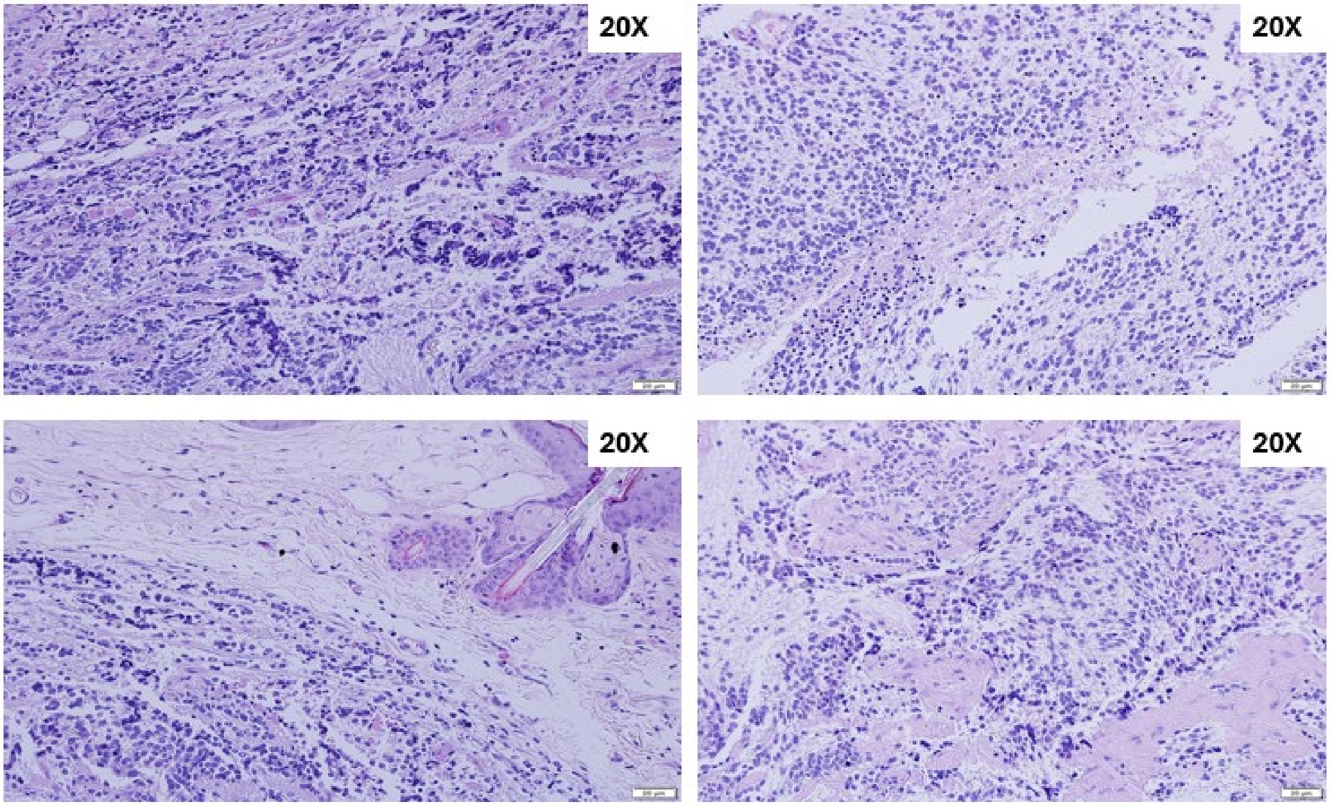


D.

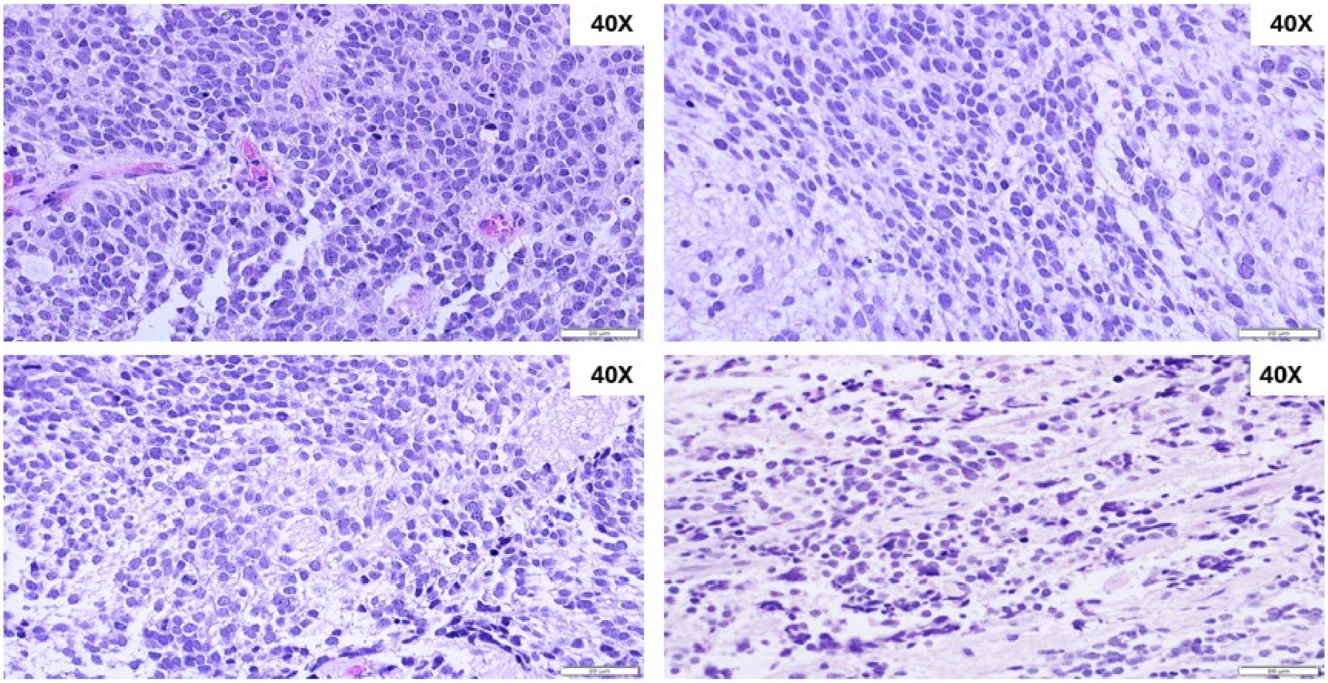

E.

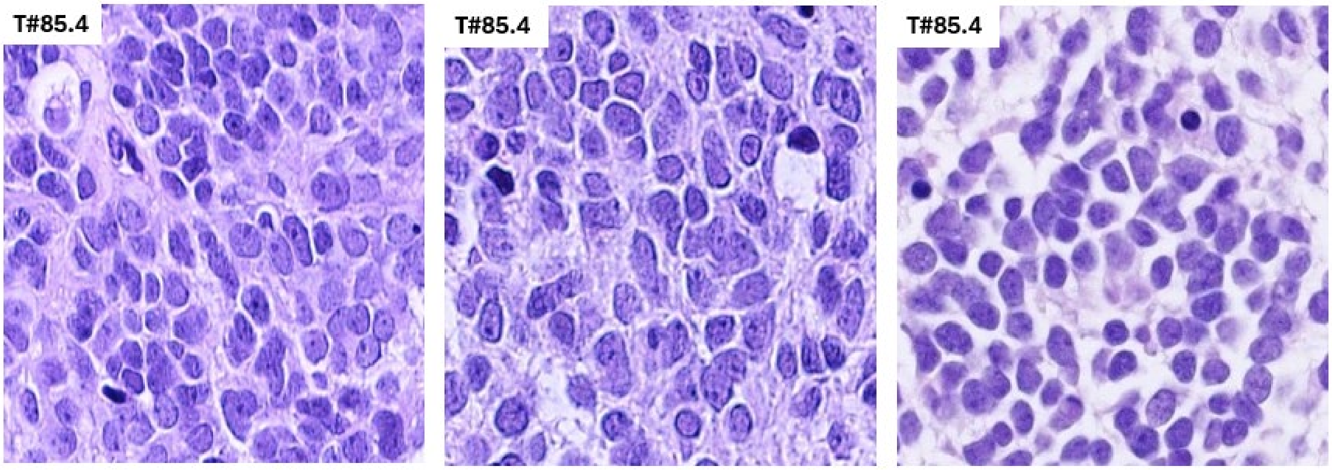


F.

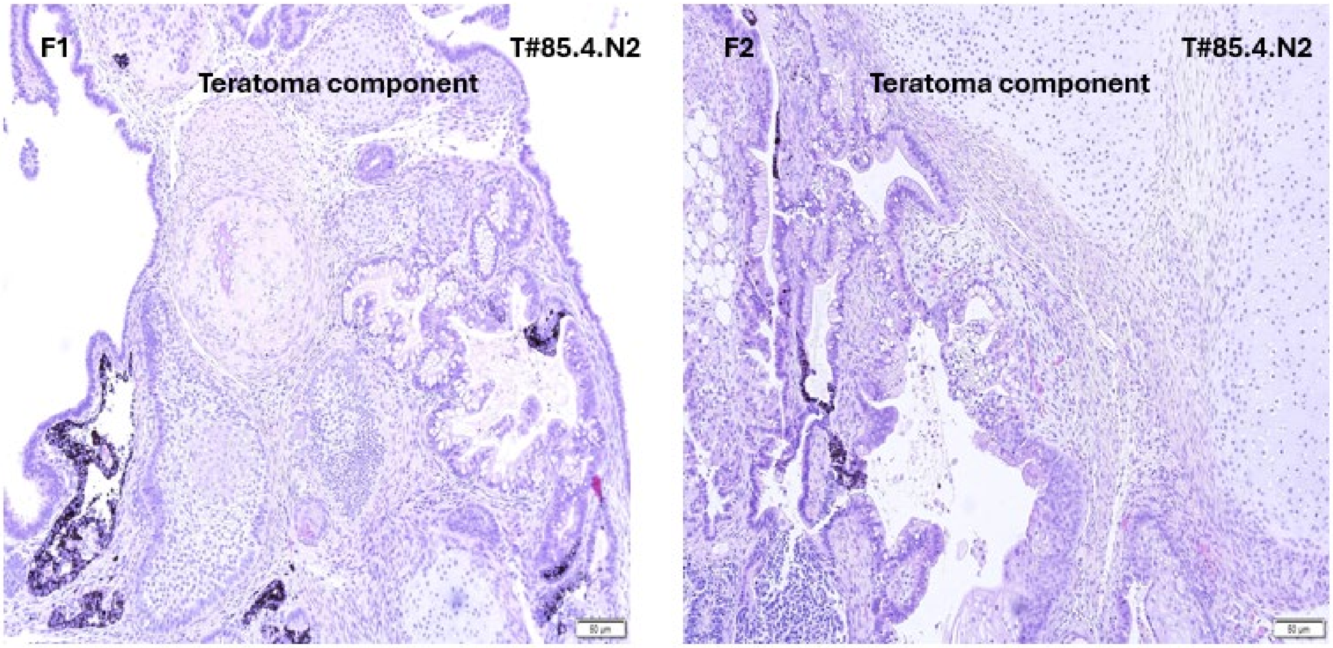


**Figure S8. Representative sections of hPGCLC_A4_L82 derived VP-MCC-like tumor consisting of two tumor nodules T#85.4.N1 & T#85.4.N2** **(A)** Serial sections of the entire T#85.4.N1 at 2X consisting of pure VMLT in sheets or occasional trabecular growth pattern (orange arrows) with a single tiny focus of epidermal squamous differentiation (black arrow) and no teratoma component. **(B)** Representative section of T#85.4.N1 at 10X. **(C)** Representative section of T#85.4.N1 at 20X. **(D)** Representative section of T#85.4.N1 at 40X. **(E)** High power fields (HPFs) show VMLT cells in both T#85.4.N1 and T#85.4.N2 with typical cytologic features of MCC including high N/C ratio, fine and salt pepper chromatin, inconspicuous nucleoli and frequent mitotic figures. **(F)** Teratoma components of T#85.4.N2 with ectodermal (squamous epidermal), mesodermal (cartilage) and endodermal (glands) differentiation.

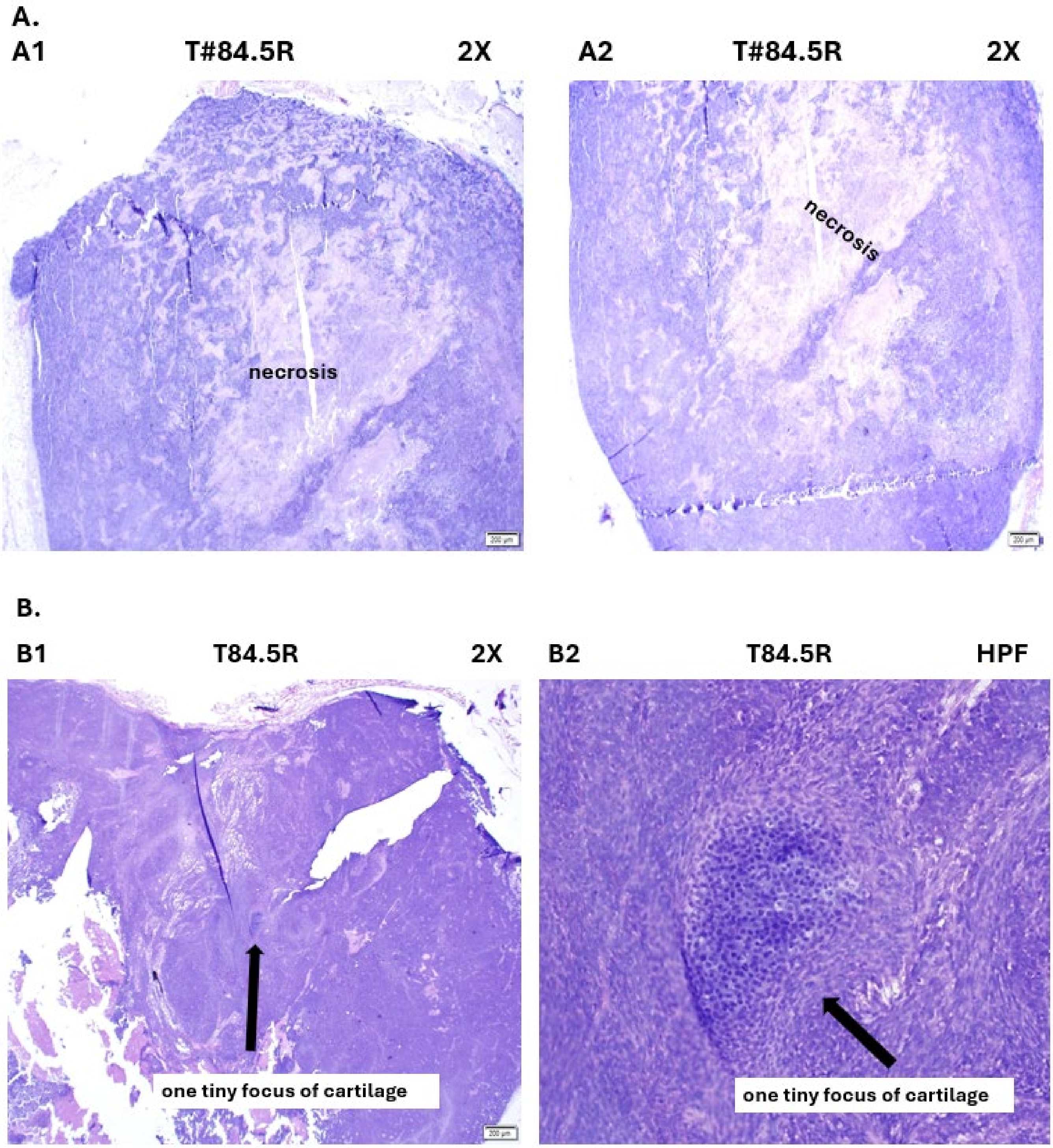
A.
A1 T#84.5R 2X
necrosis
A2 T#84.5R 2X
necrosis
B.
B1 T84.5R 2X
one tiny focus of cartilage
B2 T84.5R HPF
one tiny focus of cartilage

C.

C1 T#84.5L 2X

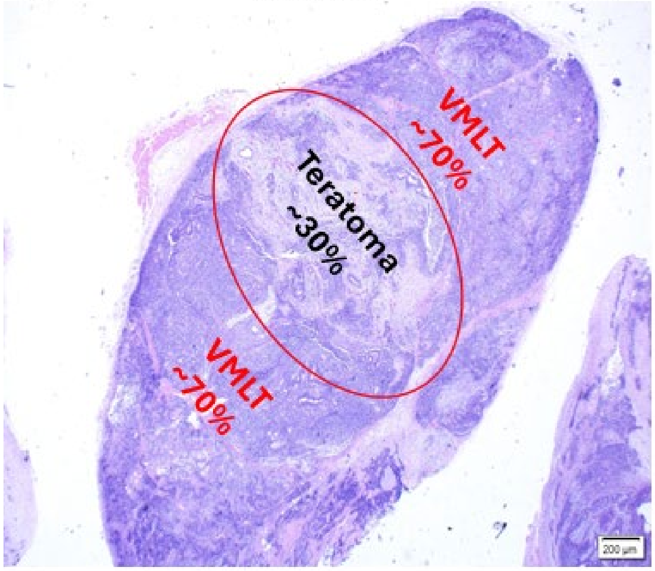


C2 T#84.5L 4X

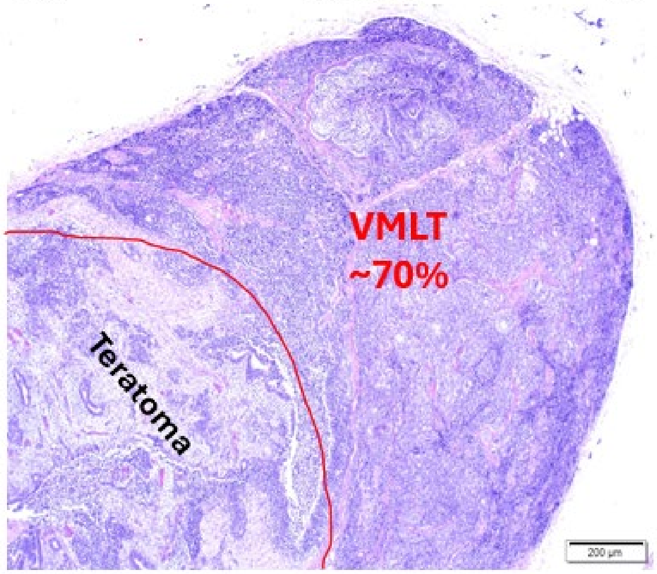


D.

D1 T#84.5R 20X

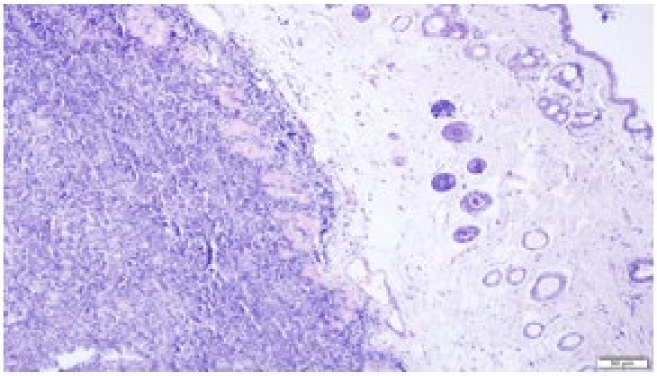

D2 T#84.5R 20X

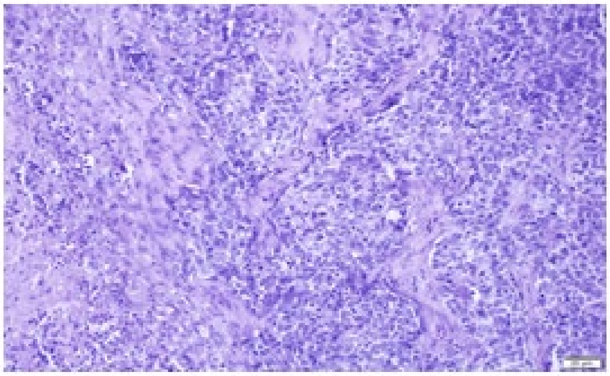

D3 T#84.5L 20X

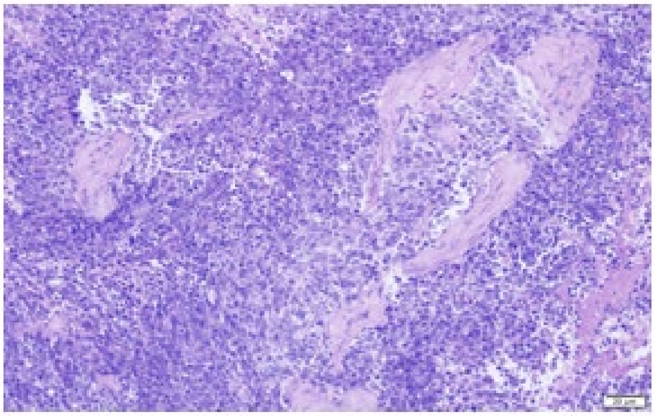

D4 T#84.5L 20X

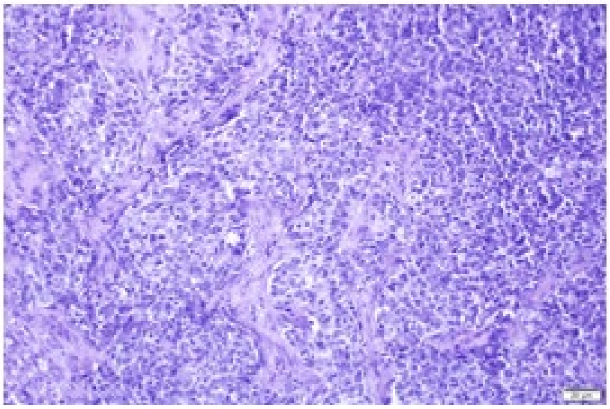

E.

E1 T#84.5R 40X

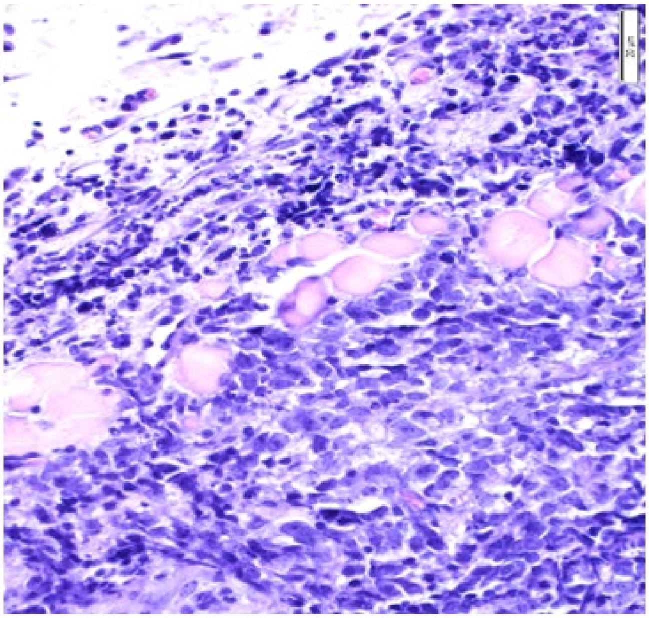

E2 T#84.5L 40X

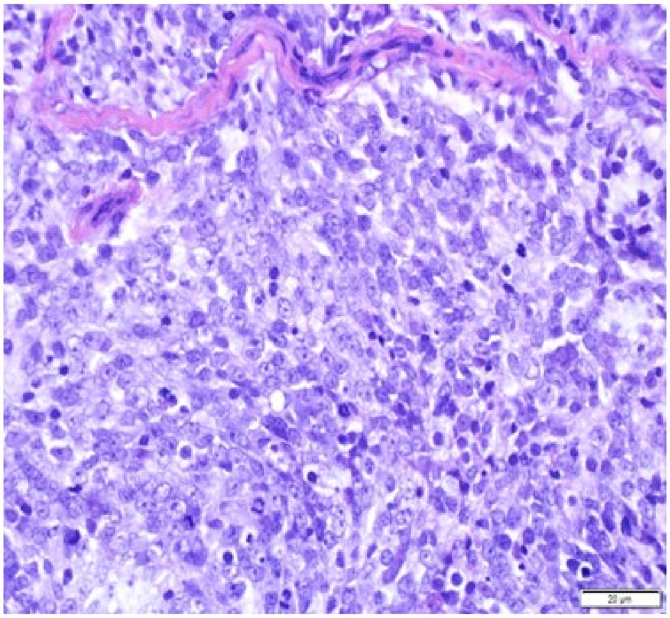

F.

F1 T#84.5R

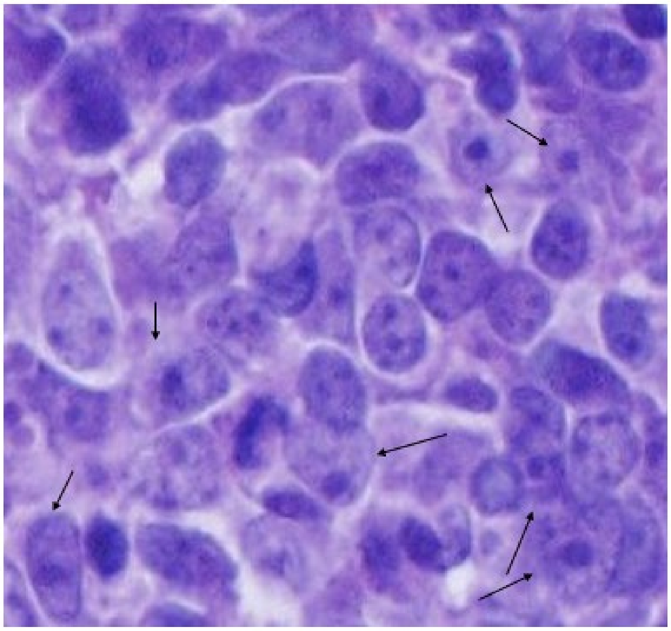

F2 T#84.5L

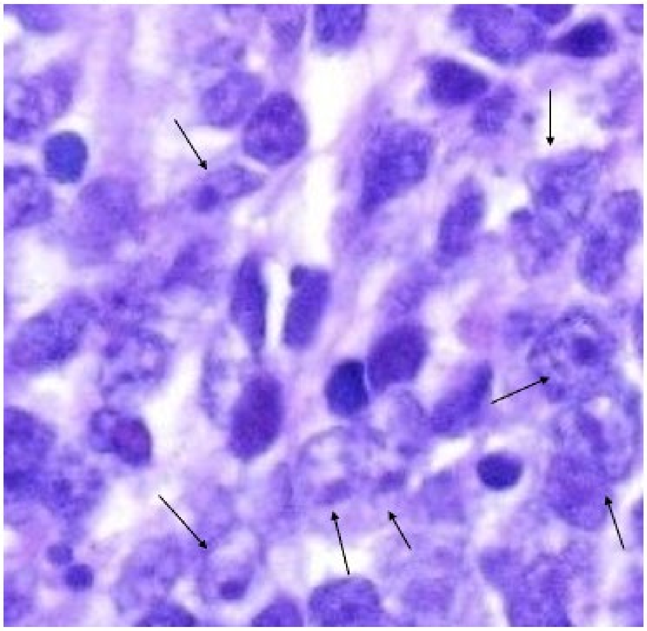

G.

G1 T#84.5L Teratoma
Endoderm & cartilage differentiation

G2 T#84.5L Teratoma
Squamous epidermal differentiation

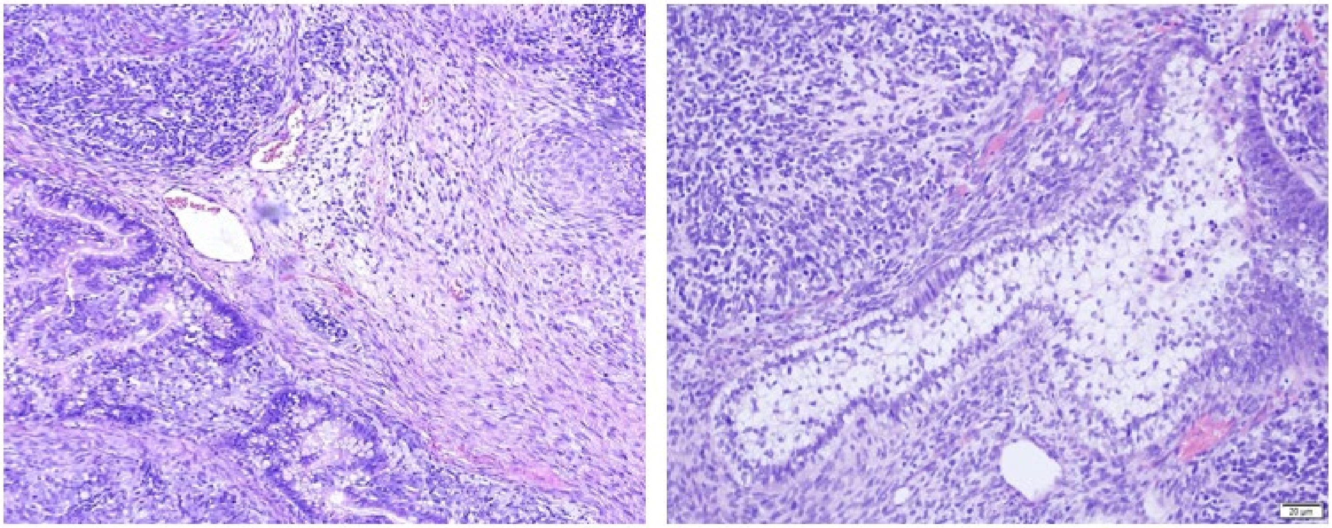


**Figure S9. Sections of VP-MCC-like tumors (VMLTs) of T#84.5R and T#84.5L. (A)** Two representative sections covering the entire T#85.1R tumor show only VMLT with central necrosis and no teratoma component (2X). **(B) (B1)** Representative section of the entire T#84.5R tumor with all VMLT except for a tiny focus of cartilage in the center (black arrow) (2X). (**B2)** HPF shows details of the tiny single focus of cartilage. **(C)** Representative section of the entire T#84.5L tumor shows a minor central teratoma component (~30% of tumor) surrounded by an extensive peripheral VMLT component (~70% of the tumor). **(D)** Sections of VMLT in T#84.5R and T#84.5L show sheets of small blue cells, with dermal invasion shown in **D1** (20X). **(E)** Sections of VMLT of T#84.5R and T#84.5L show VMLT cells infiltrated through mouse dermal striated muscle layer when invaded into dermis (40X). **(F)** HPF views of cytomorphology of VMLT cells in T#84.5R **(F1)** and T#85.1L **(F2).** As well as typical VP-MCC-like cells with salt and pepper chromatin and inconspicuous nucleoli, intermixed larger atypical cells with paler vesicular chromatin, prominent large nucleoli and more abundant cytoplasm are identified (black arrows). **(G)** Sections of central teratoma component with three germ layer differentiation in T#84.5L.

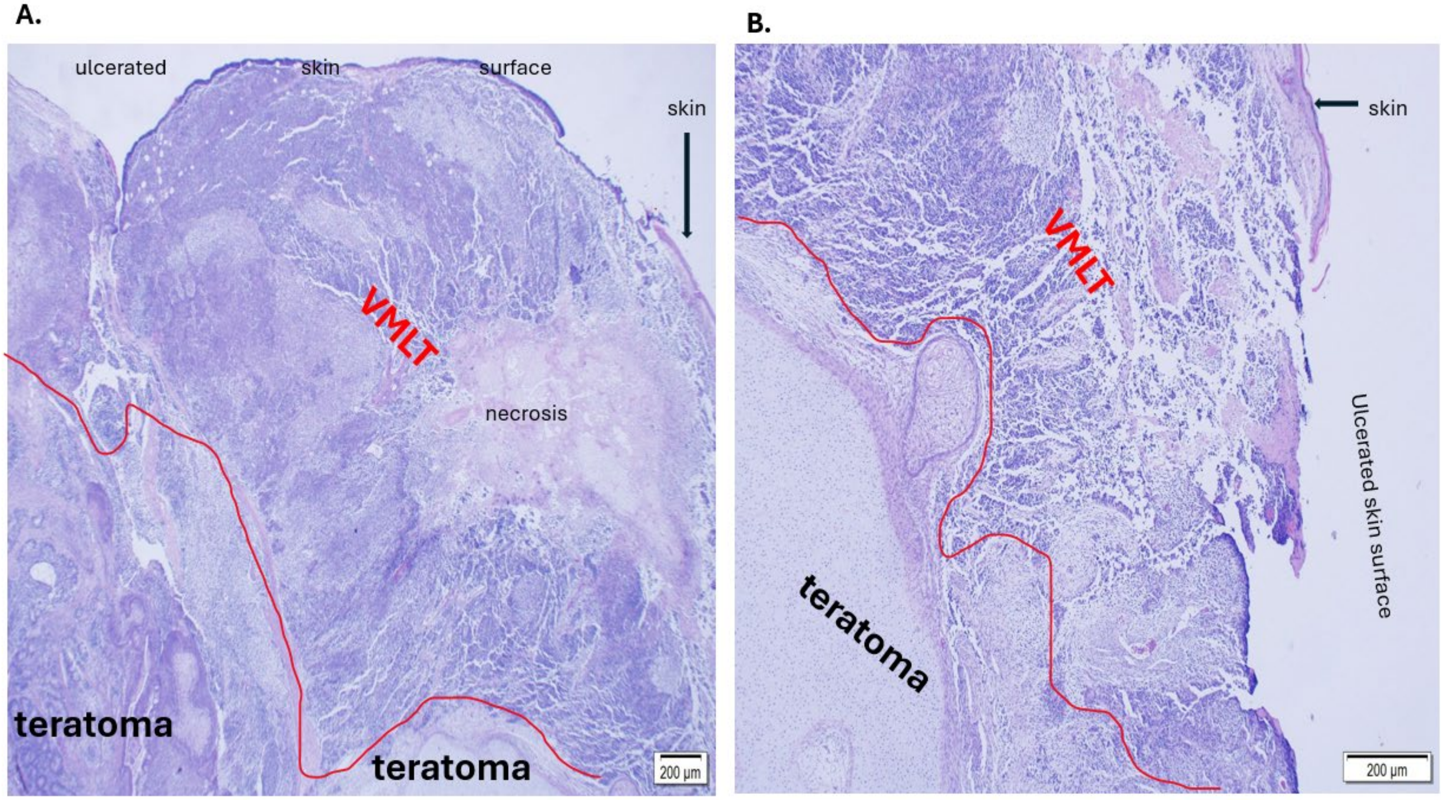


**Figure S10. Tumor T#85.3.** Large sheets of VP-MCC-like tumor cells at skin surface covering deeper teratoma component in T#85.3 tumor (2X).

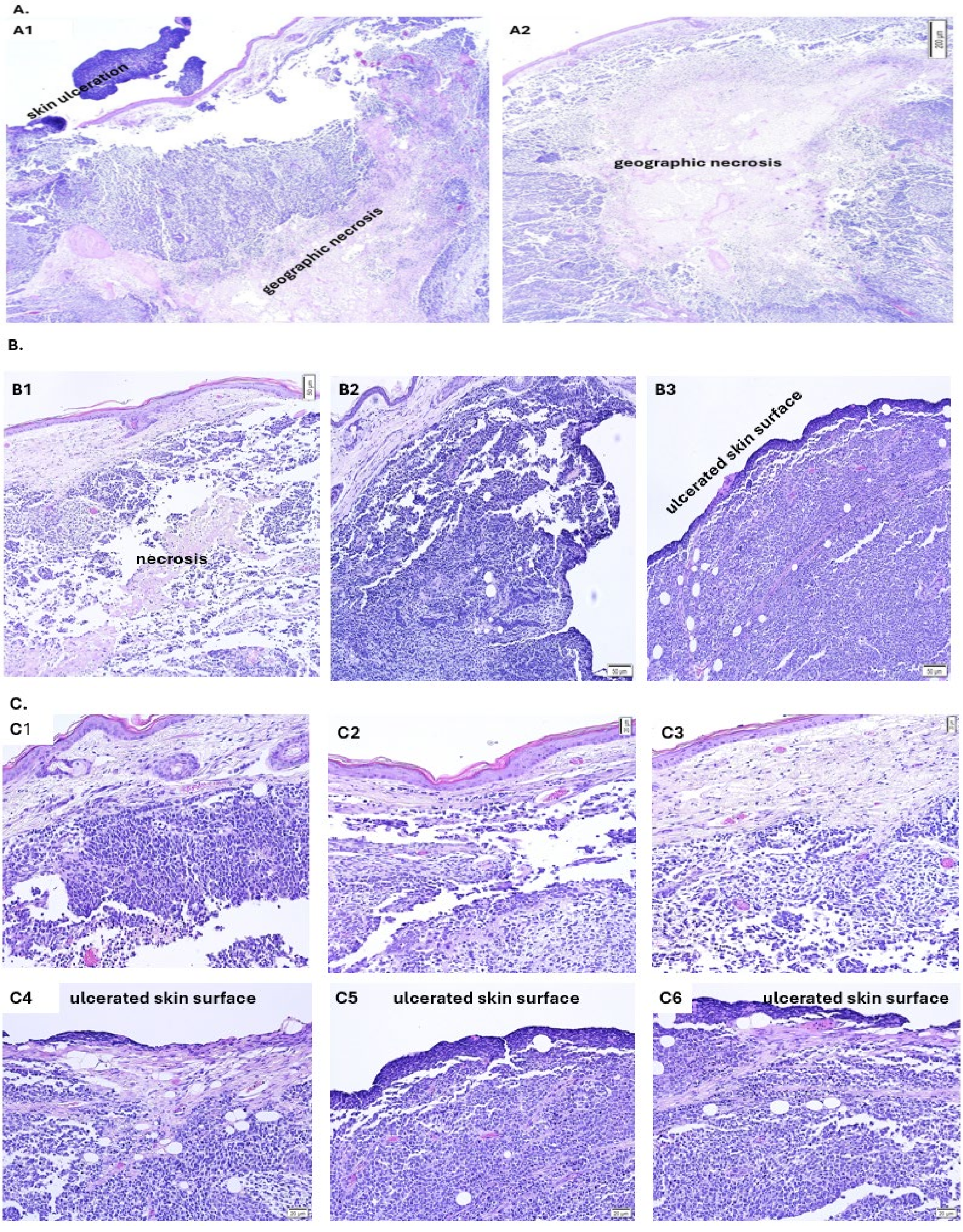
A.
A1
skin ulceration
geographic necrosis
A2
geographic necrosis
B.
B1
necrosis
B2
B3
ulcerated skin surface
C.
C1
C2
C3
C4
ulcerated skin surface
C5
ulcerated skin surface
C6
ulcerated skin surface

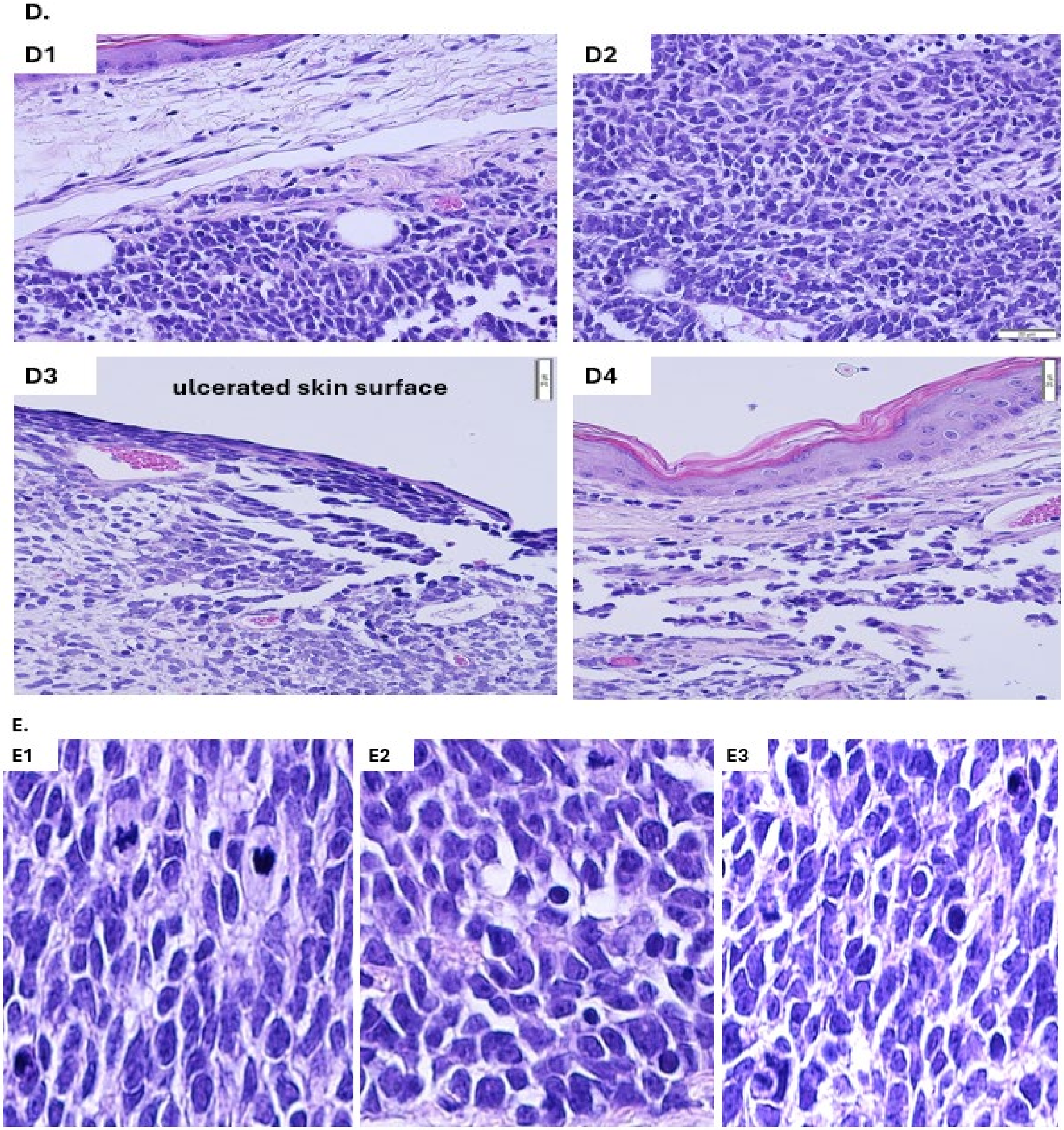


**Figure S11. Representative sections of VP-MCC-like tumors (VMLTs) in superficial mouse skin of T#85.3 with extensive necrosis and ulceration**. **(A)** 2X **(B)** 10X **(C)** 20X **(D)** 40X **(E)** HPF views of cytomorphology of VMLT cells like those of T#85.4 in Figure S5 (E).

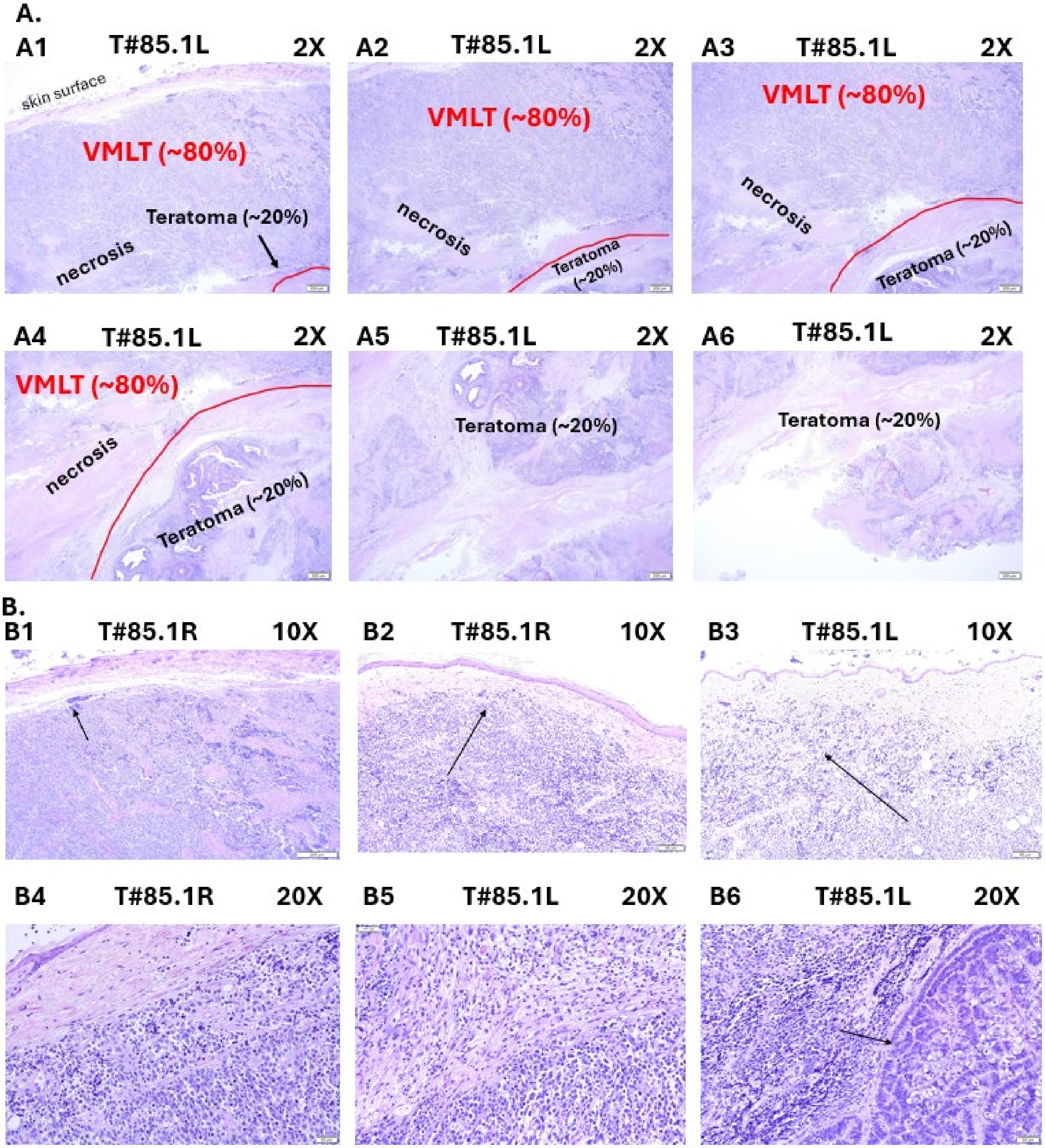

A.
A1
T#85.1L
2X
skin surface
VMLT (~80%)
Teratoma (~20%)
necrosis
A2
T#85.1L
2X
VMLT (~80%)
necrosis
Teratoma (~20%)
A3
T#85.1L
2X
VMLT (~80%)
necrosis
Teratoma (~20%)
A4
T#85.1L
2X
VMLT (~80%)
necrosis
Teratoma (~20%)
A5
T#85.1L
2X
Teratoma (~20%)
A6
T#85.1L
2X
Teratoma (~20%)
B.
B1
T#85.1R
10X
B2
T#85.1R
10X
B3
T#85.1L
10X
B4
T#85.1R
20X
B5
T#85.1L
20X
B6
T#85.1L
20X

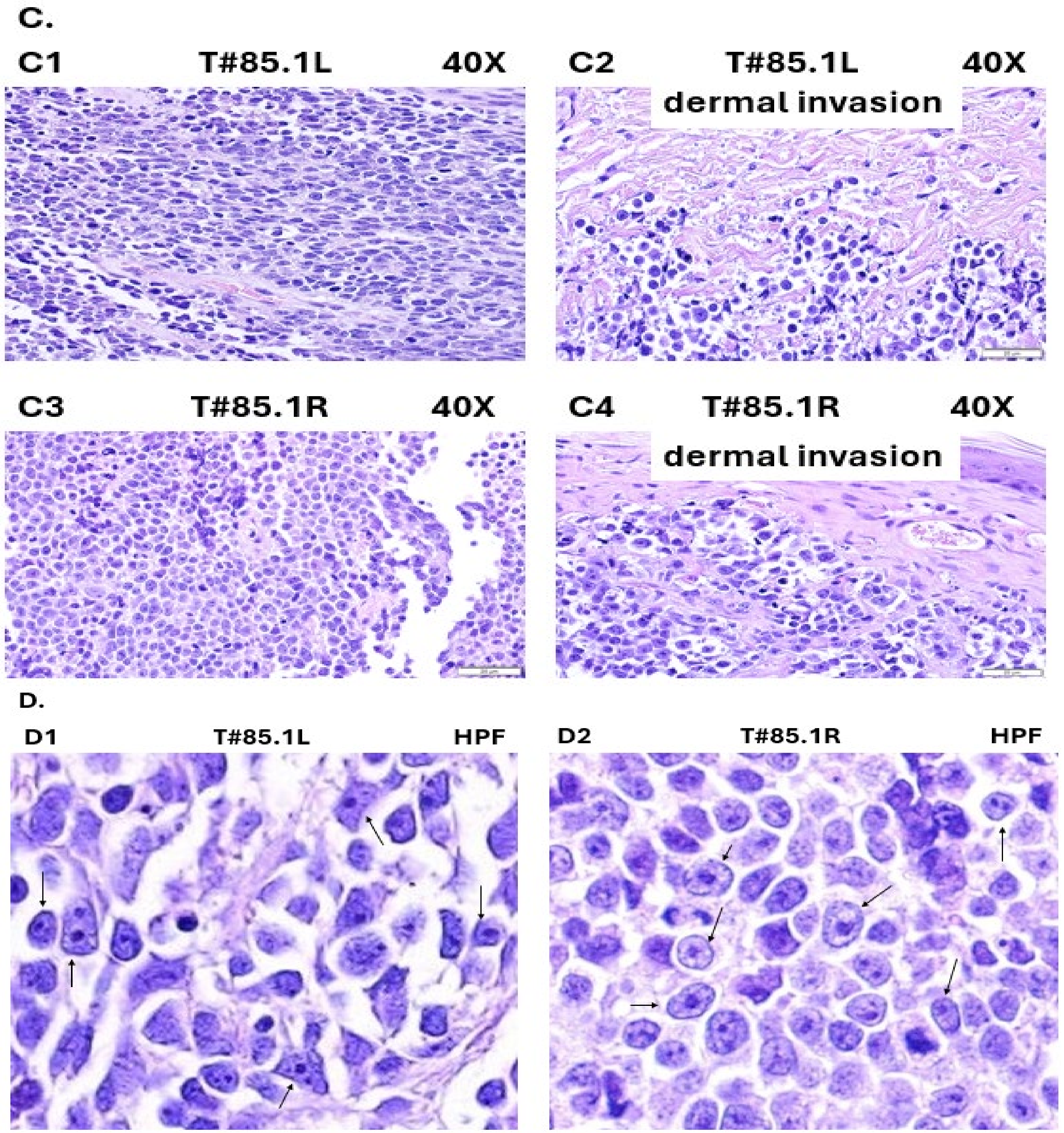


**Figure S12. Representative sections of VP-MCC-like tumors (VMLTs) of T#85.1L & T#85.1R.** (**A**) Serial sections of T#85.1L to show extensive VMLT with necrosis in superficial mouse skin to deeper minor teratoma component at 2X. (**B**) Sections of VMLT of tumor T#85.1L at 10X and 20X showed LVI (black arrow in B1), dermal invasion (B4, B5 and black arrows in B2 and B3) and sheets of small blue cells with rare foci of trabecular growth pattern (black arrow in B6). (**C**) Sections of VMLT of both T#85.1L and T#85.1R tumors at 40X showed sheets of small blue cells (C1 and C3 respectively) and dermal invasion of VMTL cells toward skin surface. (**D**) High power field views showed cytomorphology of VMLT cells of T#85.1L (D1) and T#85.1R (D2). Besides typical VP-MCC like cells with salt and pepper chromatin and inconspicuous nucleoli, intermixed larger atypical cells with paler vesicular chromatin, prominent large nucleoli and more abundant cytoplasm (black arrows) were also identified.

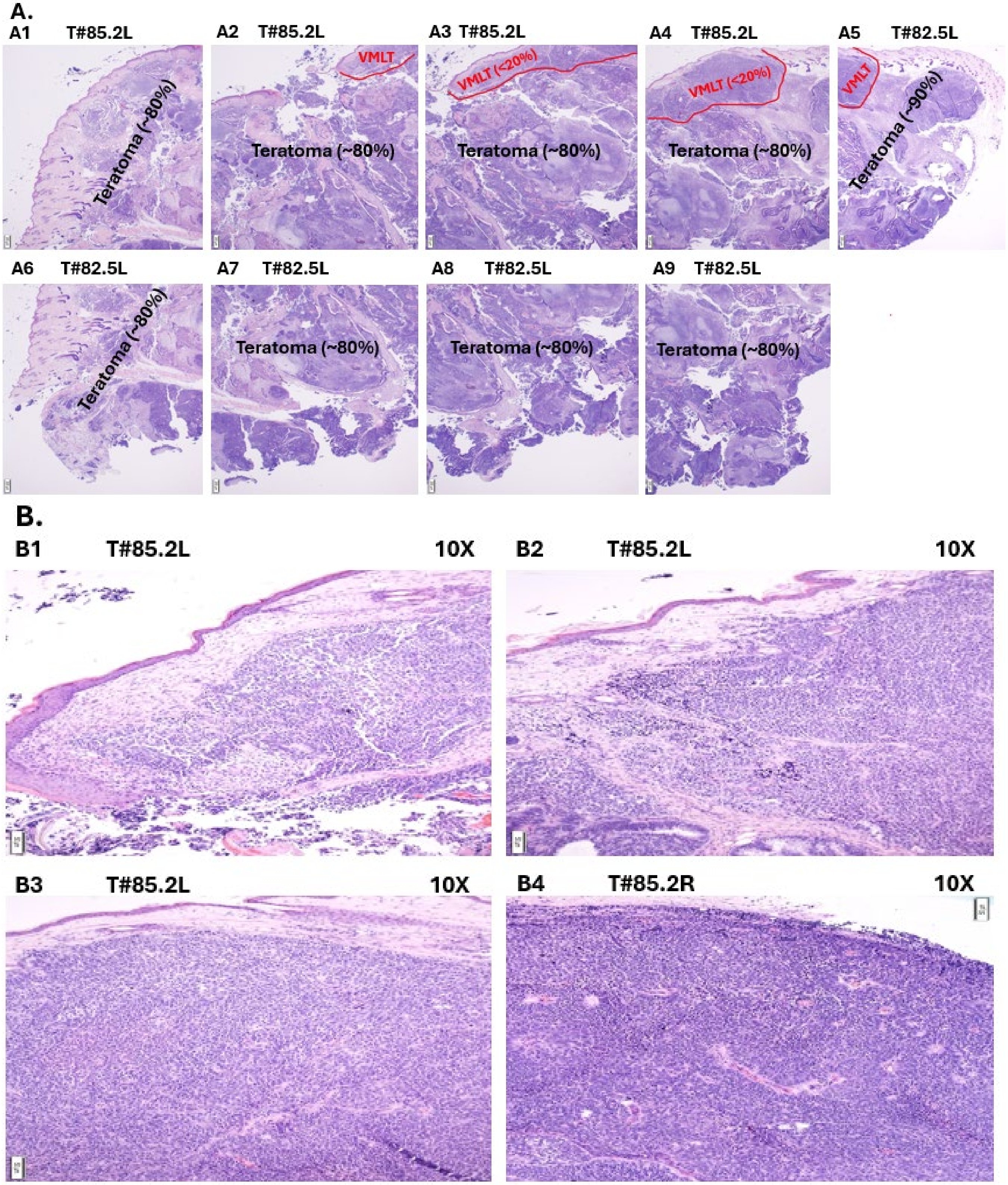
A.
A1 T#85.2L
Teratoma (~80%)
A2 T#85.2L
VMLT
Teratoma (~80%)
A3 T#85.2L
VMLT (<20%)
Teratoma (~80%)
A4 T#85.2L
VMLT (<20%)
Teratoma (~80%)
A5 T#82.5L
VMLT
Teratoma (~90%)
A6 T#82.5L
Teratoma (~80%)
A7 T#82.5L
Teratoma (~80%)
A8 T#82.5L
Teratoma (~80%)
A9 T#82.5L
Teratoma (~80%)
B.
B1 T#85.2L 10X
B2 T#85.2L 10X
B3 T#85.2L 10X
B4 T#85.2R 10X

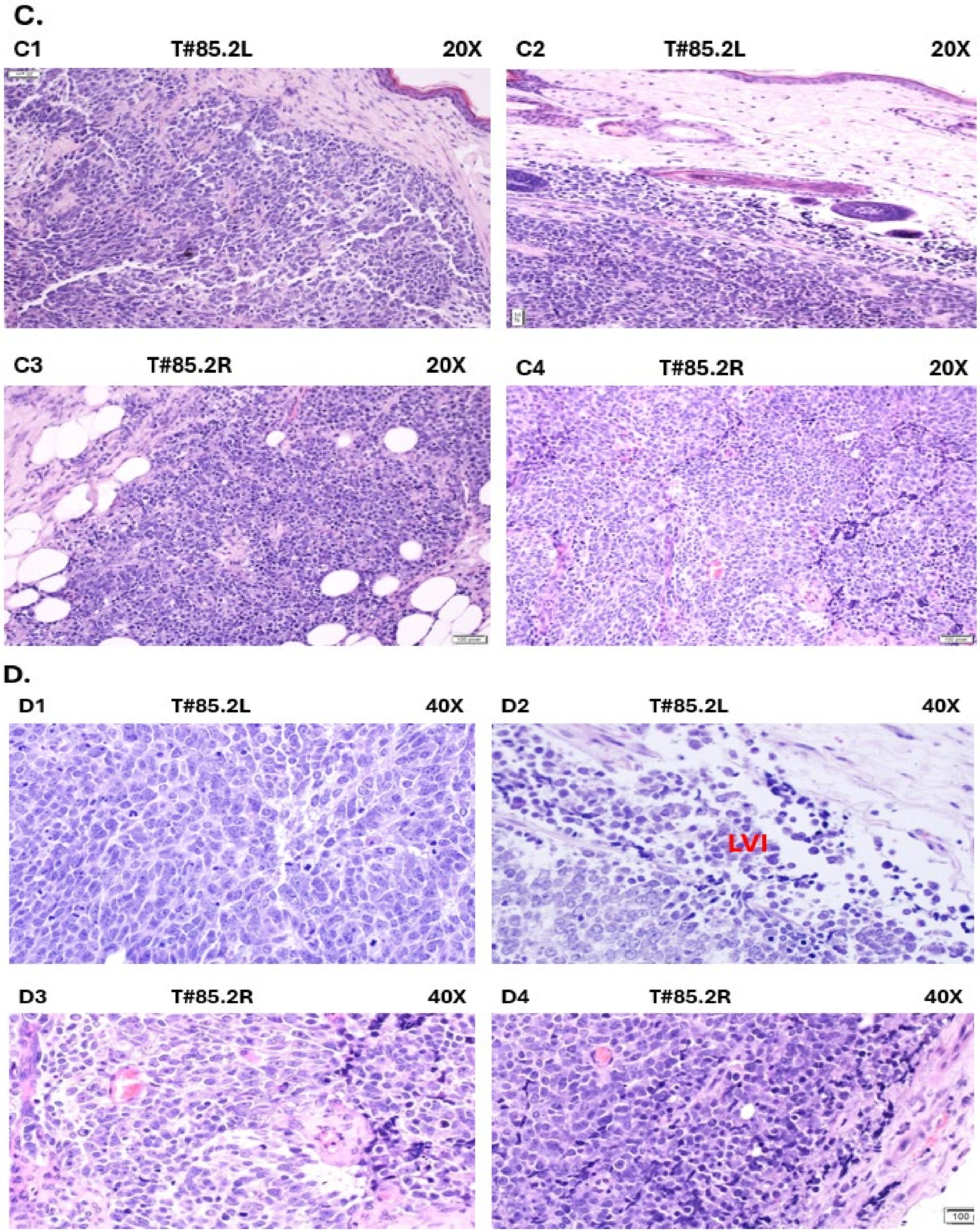
C.
C1
T#85.2L
20X
C2
T#85.2L
20X
C3
T#85.2R
20X
C4
T#85.2R
20X
D.
D1
T#85.2L
40X
D2
T#85.2L
40X
LVI
D3
T#85.2R
40X
D4
T#85.2R
40X
100

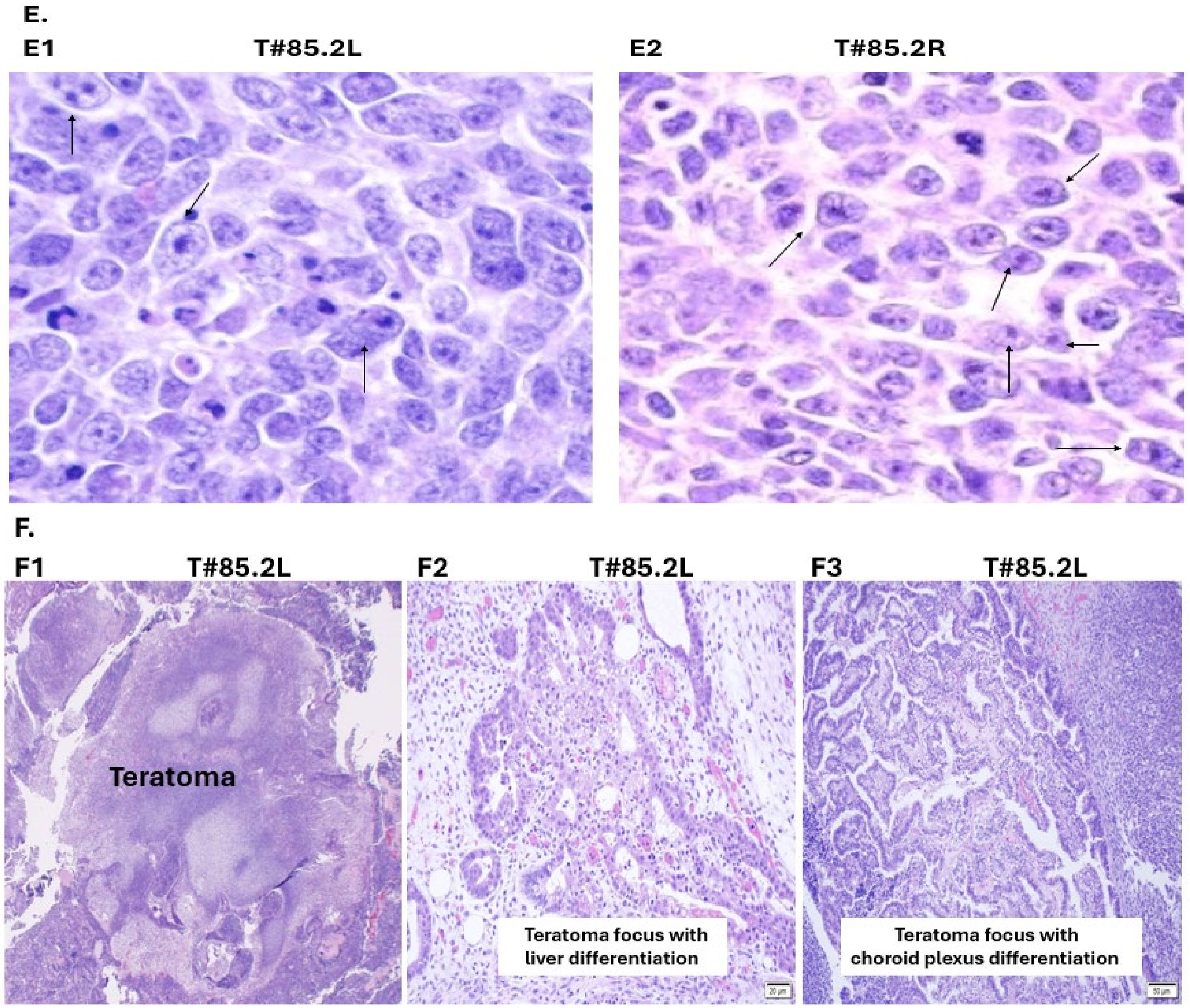


**Figure S13. Representative sections of VP-MCC-like tumors (VMLTs) of T#85.2L and T#85.2R.** (**A**) Serial sections of entire T#85.2L tumor show a minor incipient VMLT component (~20% of tumor) at right superficial mouse skin and vast majority of teratoma component (~80%) at left and deeper (2X). Sections of VMLT of tumor T#85.2L and T#85.2R at (**B**) 10X (**C**) 20X and (**D**) 40X show sheets of VMLT cells at superficial mouse skin with extensive dermal invasion (B1-B4, C1-C3, D1 and D4) and LVI (D2). (**E**) High power field views show cytomorphology of VMLT cells of T#85.2L (E1) and T#85.2R (E2). As well as typical VP-MCC-like cells with salt and pepper chromatin and inconspicuous nucleoli, intermixed larger atypical cells with paler vesicular chromatin, prominent large nucleoli and more abundant cytoplasm (black arrows) were also identified. (**F**) Extensive teratoma component with three germinal layer differentiation in T#85.2L.

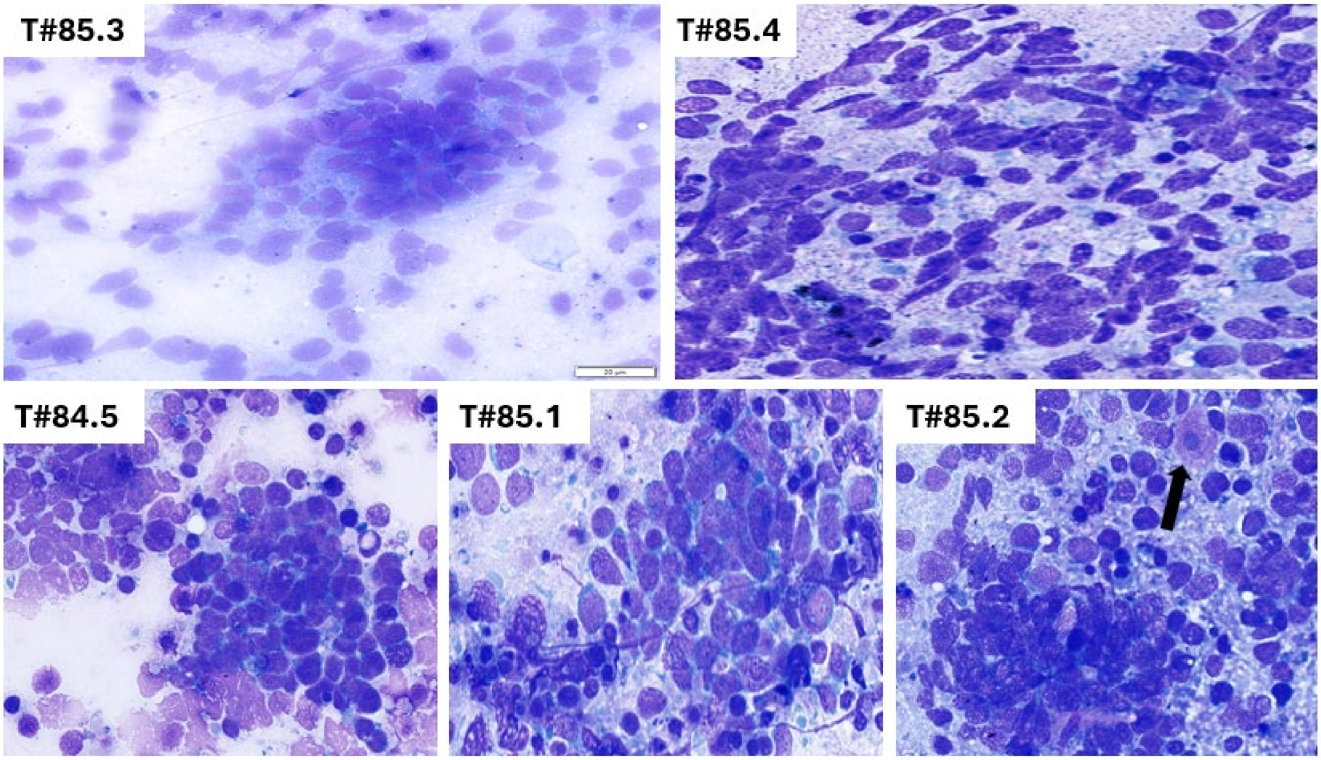


**Figure S14**. **Cytology smears of mouse xenograft VP-MCC-like tumors (VMLT tumors).** T#85.4 and T#85.3 smears show typical SCNC cytomorphology with high N/C ratio, fine chromatin, inconspicuous nucleoli, nuclear streaking and nuclear molding. T#84.5, T#85.1 and T#85.2 show some cytomorphologic features atypical for VP-MCC: more abundant cytoplasm with some VMLT cells exhibiting prominent nucleoli (black arrow in T#85.2).

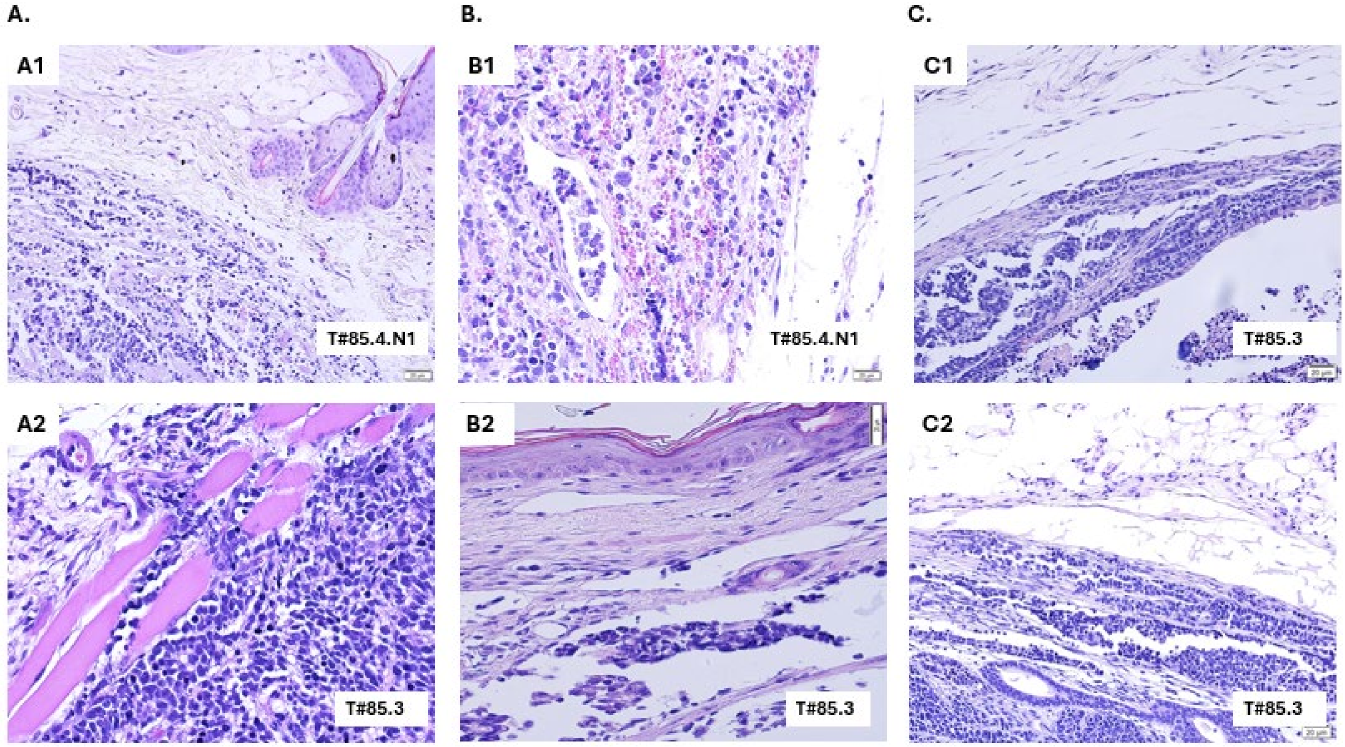


**Figure S15**. **VP-MCC tumors (VMLTs) of T#85.3 and T#85.4 exhibit malignant histological features. (A)** Dermal invasion with VMLT cells infiltrating through dermal striated muscle layer in T#85.4 and T#85.3. **(B)** Lymphovascular invasion (LVI) with clusters of VMLT cells inside lymphatic spaces in T#85.4 and T#85.3. **(C)** Infiltration and destruction of tumor capsules by VMLT cells in T#85.4 and T#85.3.

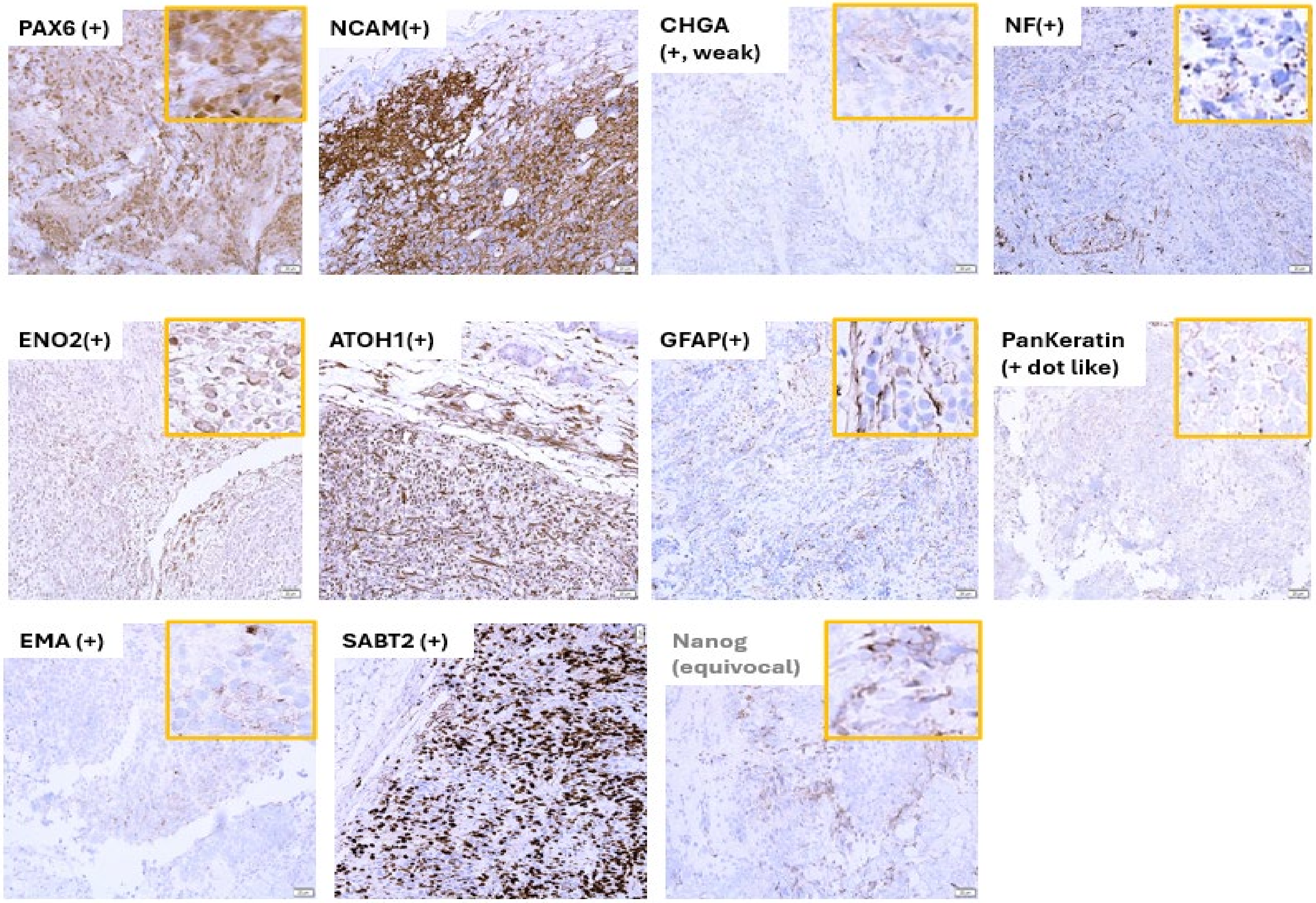


**Figure S16. Additional IHC studies of T#85.4.** T#85.4 shows protein expression profile typical for VP-MCC, including neuroendocrine genes, PAX6, NCAM, CHGN, NF, ENO2, ATOH, GFAP, epithelial genes Pan keratin and EMA, as well as SABT2. Nanog IHC stain is probably negative with only cytoplasmic staining.

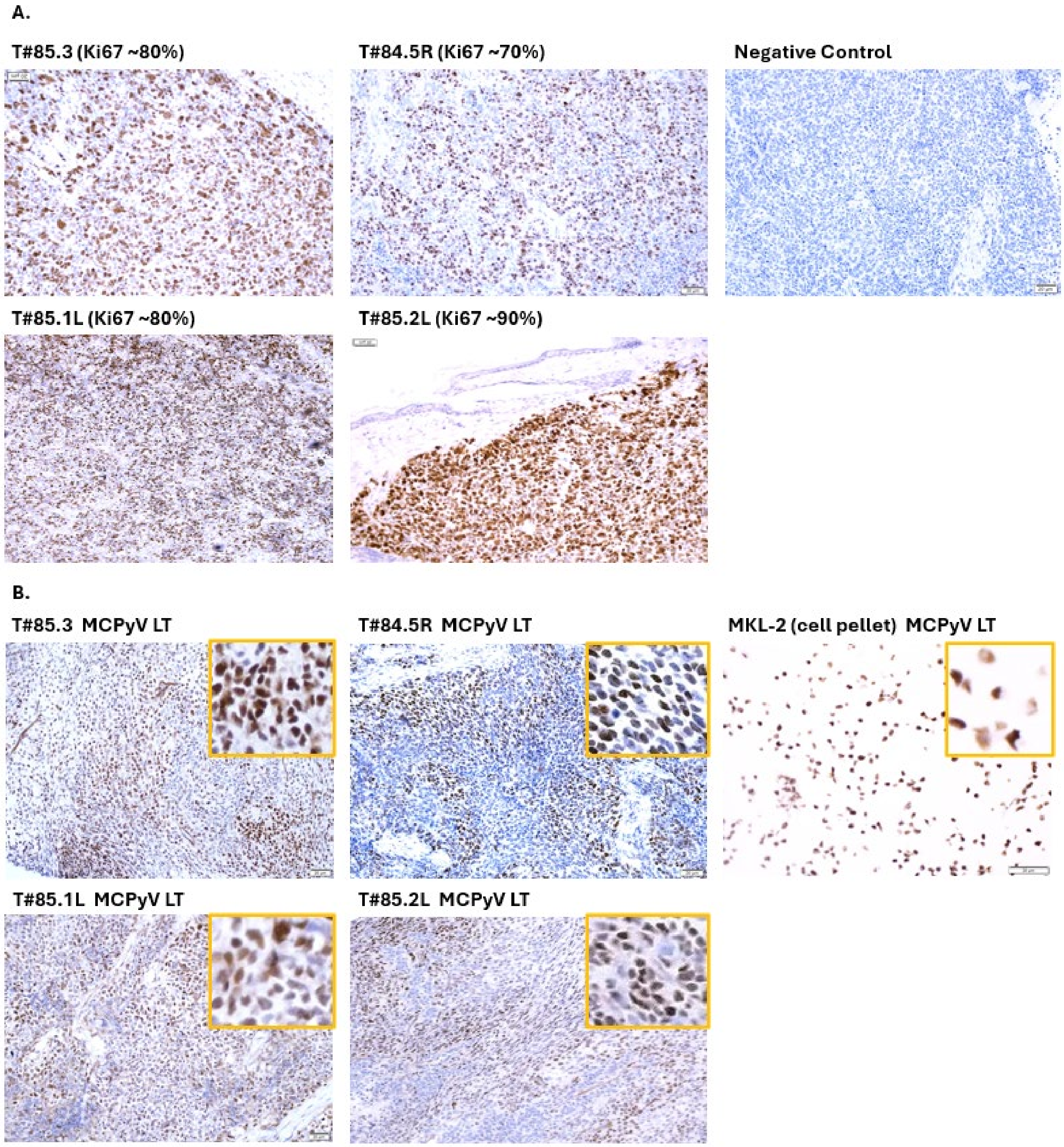


**Figure S17. IHC studies of Ki67 and MCPyV LT (A)** IHC stains of high Ki67 proliferative indexes; and (**B**) positive MCPyV in VP-MCC like tumors T#85.3 derived from hPGCLC_A4_L82, and T#84.5R, T#85.1L and T#85.2L from hiPSC_A4_:82.

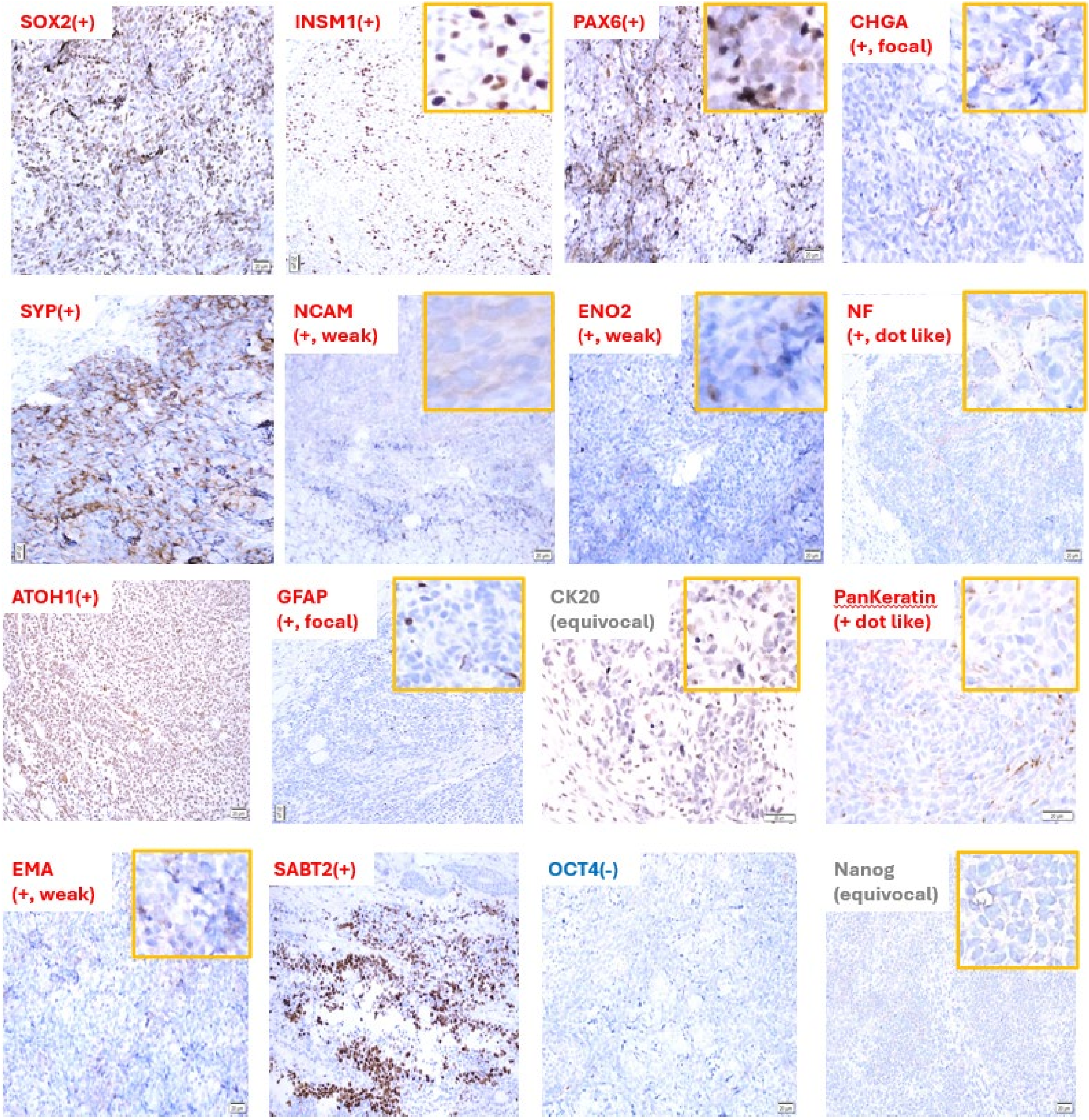


**Figure S18. Immunohistochemical stains of VP-MCC-like tumor T#85.3.**

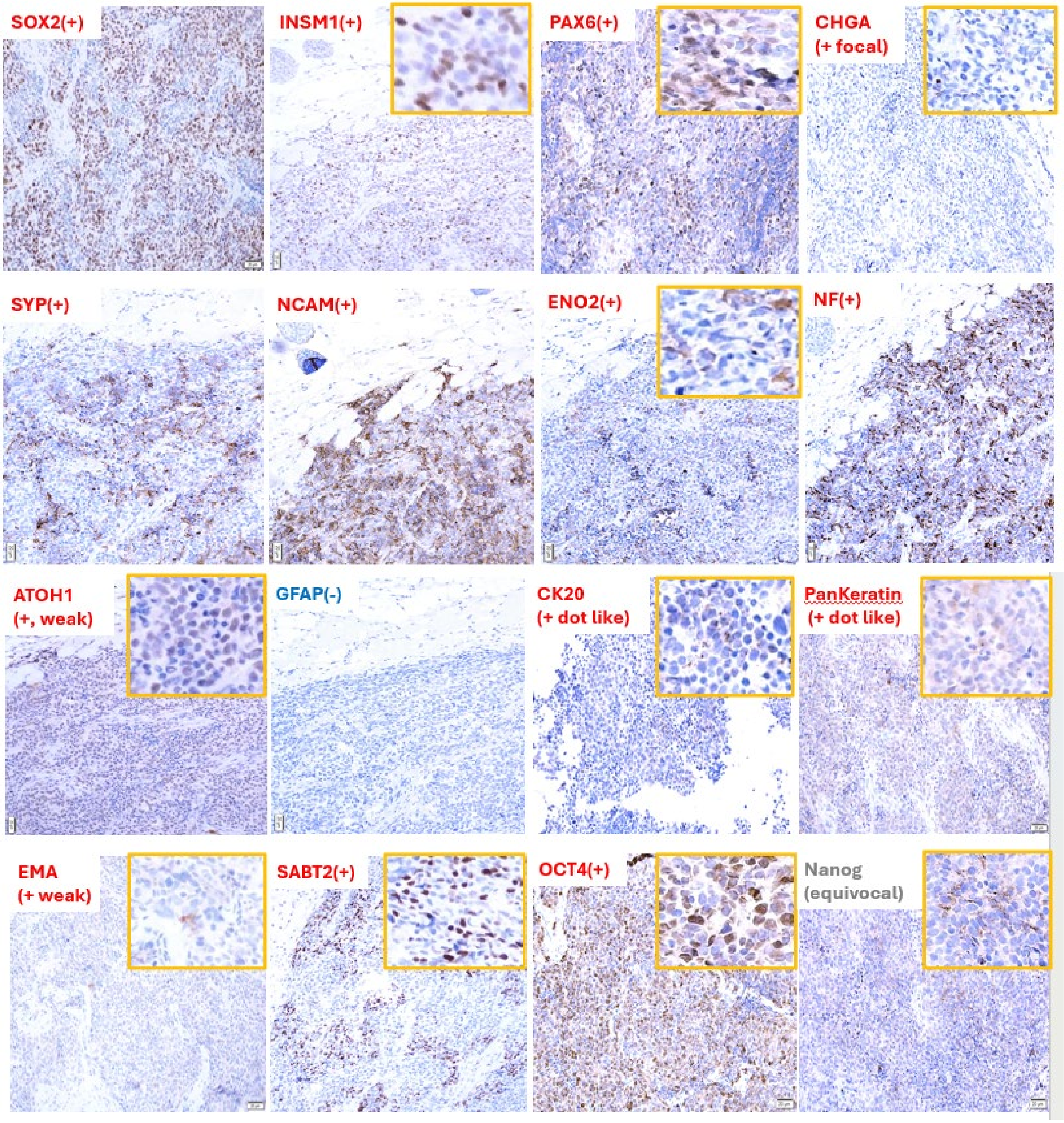


**Figure S19. Immunohistochemical stains of VP-MCC-like tumor T#84.5R.**

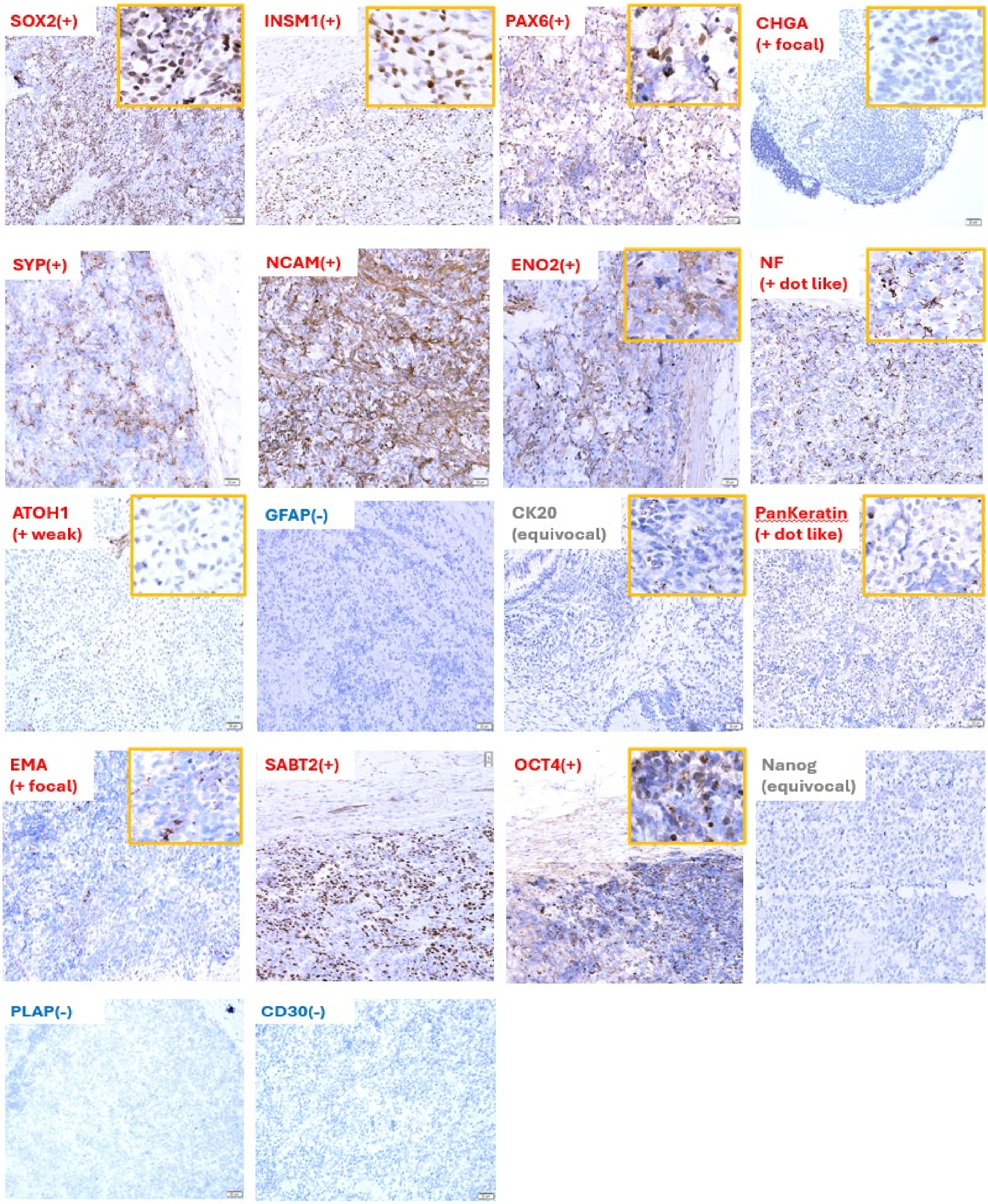


**Figure S20. Immunohistochemical stains of VP-MCC-like tumor T#85.1L.**

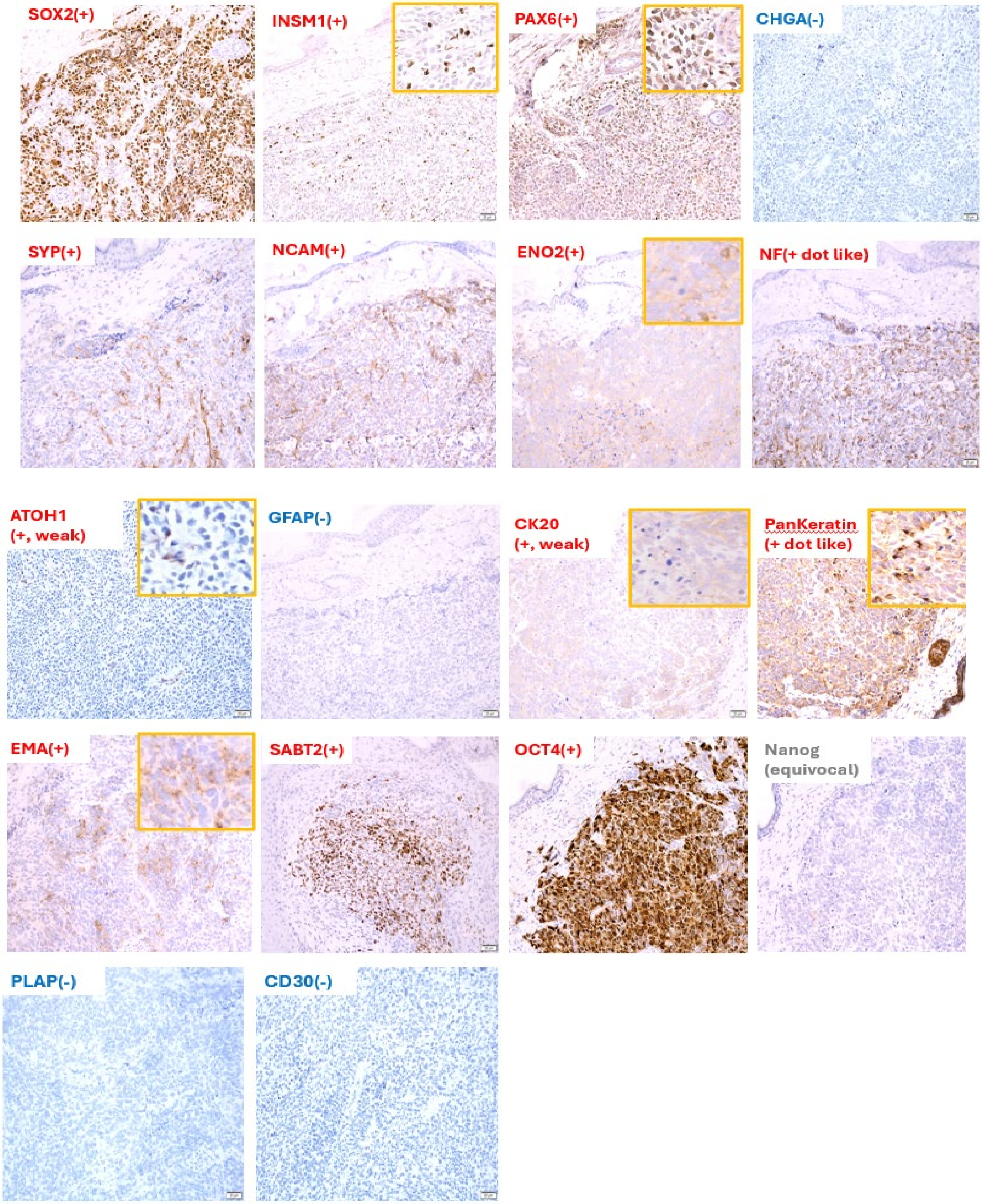


**Figure S21. Immunohistochemical stains of VP-MCC-like tumor T#85.2L.**

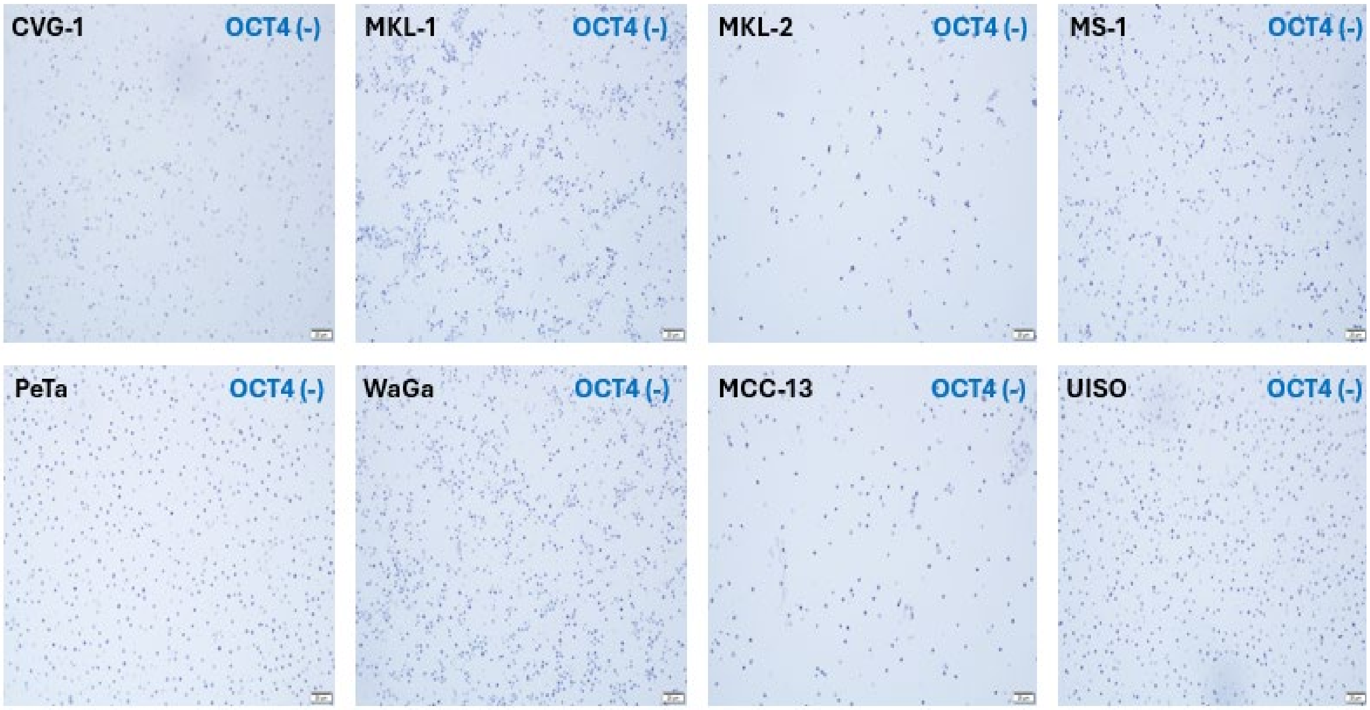


**Figure S22. Immunohistochemical stains of OCT4(-) in VP-MCC and VN-MCC cell lines.**

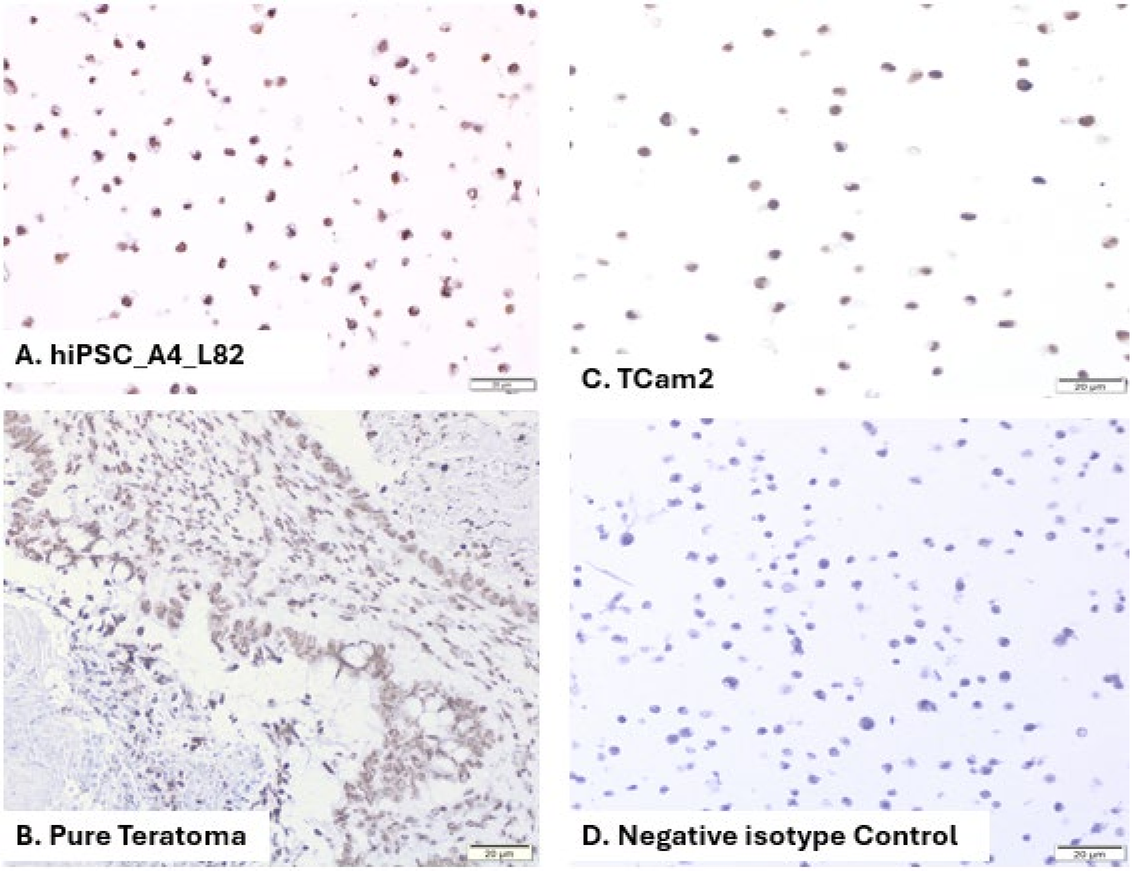


**Figure S23. Additional 5mC IHC studies. (A) hiPSC_A4_L82cell pellet, and (B) Teratoma from a pure teratoma tumor (T#83.5) Show strong 5mC stains, (C) TCam2 line cell pellet, and (D) Isotype negative control of TCam2.**

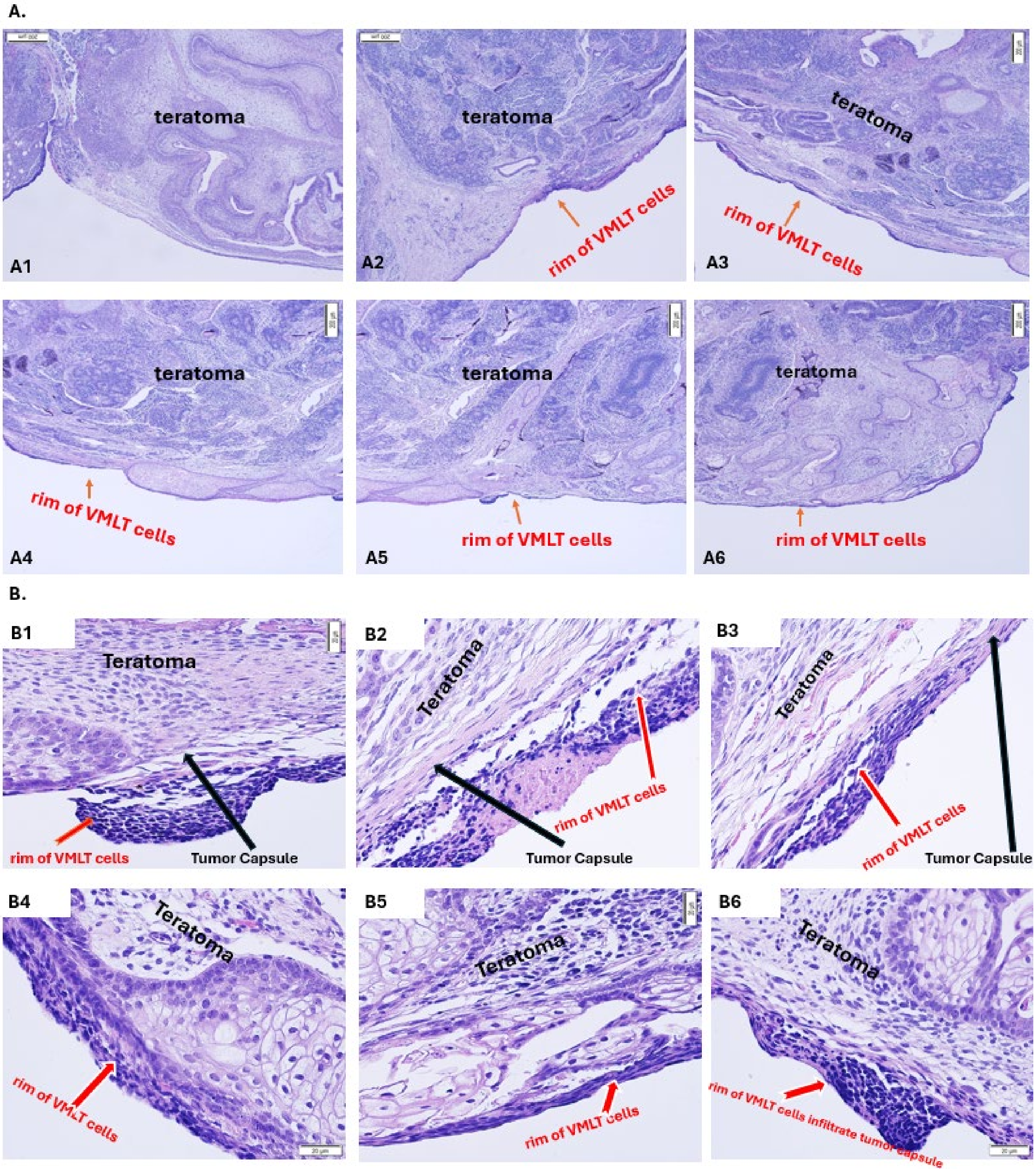
A.
teratoma
A1
teratoma
rim of VMLT cells
A2
teratoma
rim of VMLT cells
A3
teratoma
rim of VMLT cells
A4
teratoma
rim of VMLT cells
A5
teratoma
rim of VMLT cells
A6
B.
B1
Teratoma
rim of VMLT cells
Tumor Capsule
B2
Teratoma
rim of VMLT cells
Tumor Capsule
B3
Teratoma
rim of VMLT cells
Tumor Capsule
B4
Teratoma
rim of VMLT cells
B5
Teratoma
rim of VMLT
B6
Teratoma
rim of VMLT cells infiltrate tumor capsule

C.

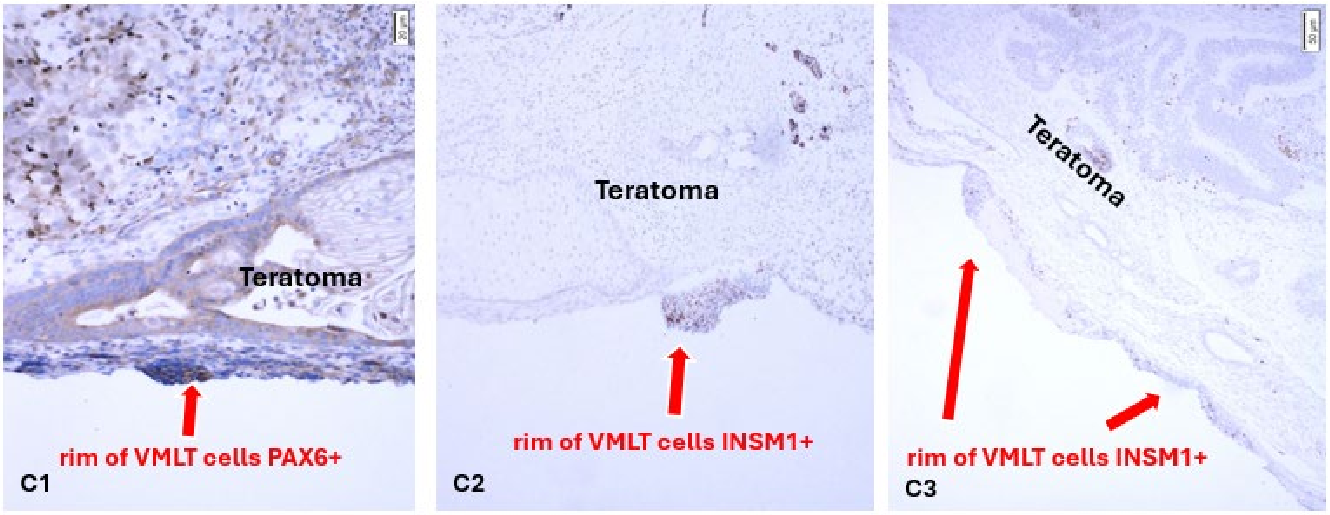


**Figure S24. Rim of VP-MCC-like tumor (VMLT) cells infiltrate deep tumor capsule covering teratoma component with a differentiation gradient in VMLT T#85.3. (A)** Serial sections of the entire deep tumor capsular surface covering teratoma component above. A thin dark rim of VMLT cells can be seen lining the deep tumor capsule. A differentiation gradient can be seen in teratoma with the outer portion more mature. (2X). **(B)** HPF view of the dark rim shows it is indeed composed of VMLT cells. Differentiated epidermal epithelium and hair follicles are at the outermost part of the teratoma. **(C)** IHC staining shows the dark rim cells are INSM1(+) and PAX6(+), consistent with VMLT cell identity.

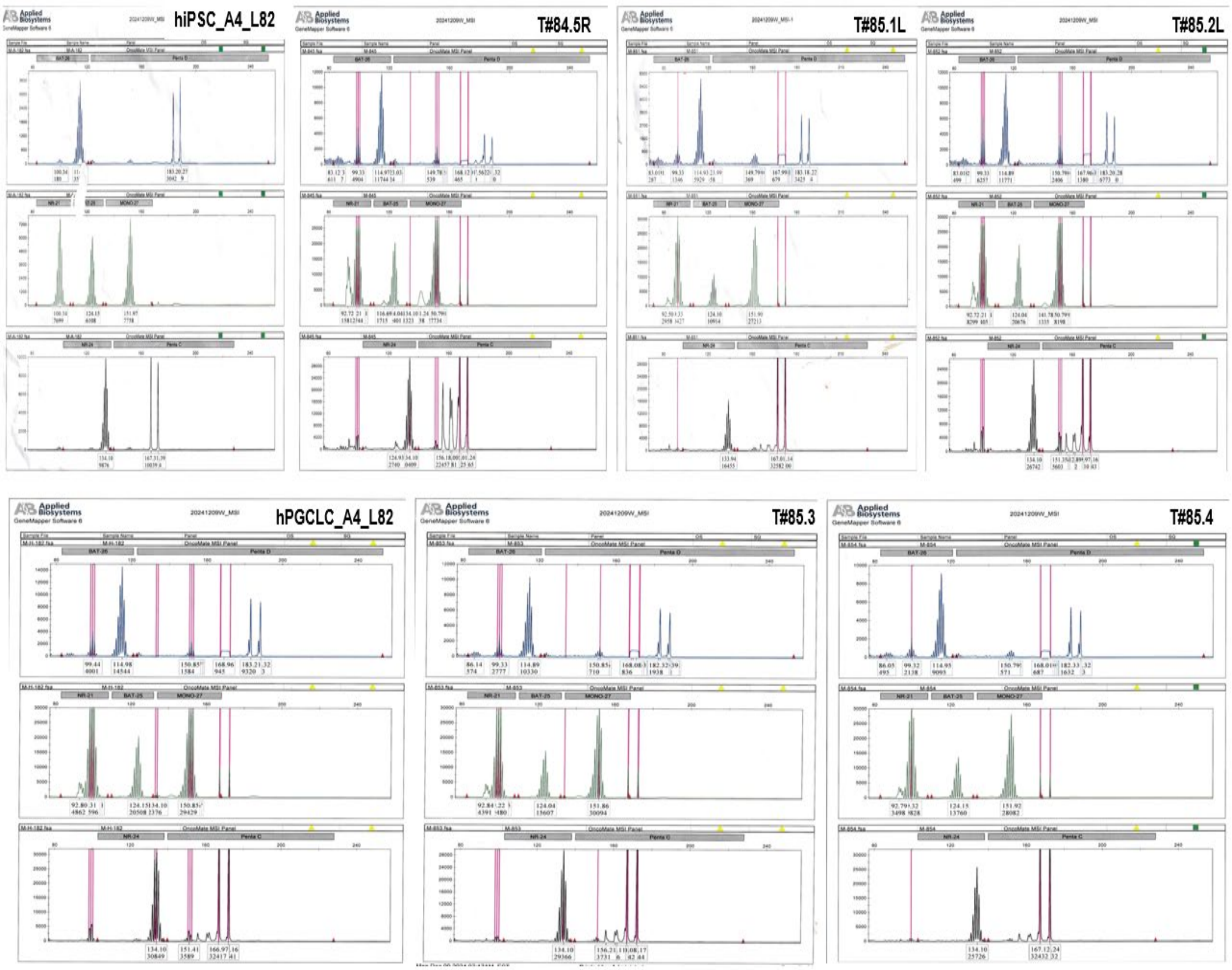


**Figure S25. Promega OncoMate MSI Analysis System was used for authentication of injected primeval stem cell lines and derived VP-MCC-like tumor (VMLT) (+) tumors.** hiPSC_A4_L82 and derived VMLT(+) tumors T#84.5R, T#85.1L and T#85.2L as well as hPGCLC_A4_L82 and its derived VMLT(+) tumors T#85.3 and T#85.4 exhibit the same STR patterns including 5 mononucleotide repeats (NR-21, BAT-26, BAT-25, NR-24, MONO-27) and two dinucleotide repeats (Penta C, and Penta D).

## Tables S1, S3, S4, S5, S6:

| Cage# | Mouse# | Physiological State Modeled | Cell Line Injected | Cell Number Injected | Time Length (Injection to Palpable Tumor) | Time Length (Palpable Tumor to Euthanasia) | VMLT Component % | Teratoma Component |
|---|---|---|---|---|---|---|---|---|
| 3783 | 1 | primed hESCs with a small subset of naïve hESCs | hiPSC_A4_L82 | 1,000,000 | 13 days | 77 days | 0 | (+) |
| | 2 | | hiPSC_A4_L82 | 1,000,000 | 27 days | 55 days | 0 | (+) |
| | 3 | | hiPSC_A4_L82 | 1,000,000 | 13 days | 70 days | 0 | (+) |
| | 4 | hEGCs (in vitro parthenogenically derives of pre-migratory hPGCs) | hEGCLC_A4_L82 | 1,000,000 | 35 days | 91 days | 0 | (+) |
| | 5 | | hEGCLC_A4_L82 | 1,000,000 | 27 days | 77 days | 0 | (+) |
| 3784 | 1 | | hEGCLC_A4_L82 | 1,000,000 | 35 days | 53 days | 0 | (+) |
| | 2 | | hEGCLC_A4_L82 | 20,000,000 | 49 days | 26 days | 0 | (+) |
| | 3 | | hEGCLC_A4_L82 | 20,000,000 | 12 days | 102 days | 0 | (+) |
| | 4 | | hEGCLC_A4_L82 | 20,000,000 | 14 days | 100 days | 0 | (+) |
| | 5 | primed hESCs with a small subset of naïve hESCs | hiPSC_A4_L82 | 20,000,000 | 17 days | 54 days | 84.5R: ~100%, 84.5L:~70% | (+) |
| 3785 | 1 | | hiPSC_A4_L82 | 20,000,000 | 09 days | 78 days | 85.1R & 85.1L: ~80% | (+) |
| | 2 | | hiPSC_A4_L82 | 20,000,000 | 17 days | 24 days | 85.2R: ~40%, 85.2L: ~20% | (+) |
| | 3 | early -hPGCs | hPGCLC_A4_L82 | 300,000 | 45 days | 53 days | ~30% | (+) |
| | 4 | | hPGCLC_A4_L82 | 300,000 | 61 days | 49 days | N1: 100%, N2: ~50% (2 tumor nodules) | N1 (-), N2 (+) |
| 3786 | 1 | | hPGCLC_A4_L82 | 300,000 | N/A | N/A (89 days from injection) | N/A | N/A |
| | 2 | | hPGCLC_A4 | 300,000 | N/A | N/A (84 days from injection) | N/A | N/A |
| | 3 | | hPGCLC_A4 | 300,000 | N/A | N/A (84 days from injection) | N/A | N/A |
| | 4 | | hPGCLC_A4 | 300,000 | N/A | N/A (84 days from injection) | N/A | N/A |

**Table S1. Mouse Xenograft Study Summary**

**Table S2. (separate file)**

**RT-qPCR Results of hPGCLC_A4_L82, hiPSC_A4_L82, five VMLTs, six VP-MCC cell lines and two VN-MCC cell lines (S2A) and statistical analysis (S2B and S2C).**

| Genes | Mouse #84.5R | Mouse #85.1L | Mouse #85.2L | Mouse #85.3 | Mouse #85.4.N1 |
|---|---|---|---|---|---|
| INSM1 | (+) | (+) | (+) | (+) | (+) |
| Chromogranin | (+) | (-?) | (-?) | (+) | (+) |
| Synaptophasin | (+) | (+) | (+) | (+) | (+) |
| NCAM | (+) | (+) | (+) | (+) | (+) |
| Pax6 | (+) | (+) | (+) | (+) | (+) |
| ATOH1 | (+) | (+) | (+) | (+) | (+) |
| Neurofilament (NF) | (+) | (+) | (+) | (+) | (+) |
| NSE | (+) | (+) | (+) | (+) | (+) |
| GFAP | (-) | (-) | (-) | (+) | (+) |
| | | | | | |
| PanKeratin | (+) | (+) | (+) | (+) | (+) |
| KRT20 | (+) | (-?) | (+) | (-?) | (+) |
| EMA | (+) | (+) | (+) | (+) | (+) |
| | | | | | |
| 4-Oct | (+) | (+) | (+) | (-) | (-) |
| Nanog | (-?) | (-?) | (-?) | (-?) | (-?) |
| Sox2 | (+) | (+) | (+) | (+) | (+) |
| | | | | | |
| SABT2 | (+) | (+) | (+) | (+) | (+) |
| | | | | | |
| McPyv LT | (+) | (+) | (+) | (+) | (+) |

**Table S3. Results Summary of IHC Study of VP-MCC-like tumors (VMLTs).**

**Table S4. (Separate Excel file)**
**Global 5mC levels by flow cytometry. Median Fluorescence Intensity (MFI) and Standard Error of the Mean (SEM) were calculated by the FlowJo software. Statistical analysis used Student T test of equal variance, and 1 tail or 2 tail method.**

| Gene | Forward | Reverse |
|---|---|---|
| **KRT8** | AGGGCCGTGGTTGTGAAGAA | AGTGCTACCCTGCATAGCGG |
| **KRT18** | GGCATCCAGAACGAGAAGGAG | ATTGTCCACAGTATTTGCGAAGA |
| **KRT20** | GAACCTAAATGACCGTCTAGCG | GGTTTCGTACCACTGCTTGATT |
| **POU3F2/BRN2** | GTGTTCTCGCAGACCACCATCT | GCTGCGATCTTGTCTATGCTCG |
| **Pax6** | CCAGGGCAATCGGTGGTAGT | ACGGGCACTCCCGCTTATAC |
| **INSM1** | ATGGCGCAACAGGAGTCAGT | ATTGGTAGGCGAGGCGAGAC |
| **Chromogranin A** | GGTTCTTGAGAACCAGAGCAGC | GCTTCACCACTTTTCTCTGCCTC |
| **ATOH1** | CCTTCCAGCAAACAGGTGAATGG | GAACGACGGGATAACATTGCGC |
| **Synaptophysin** | GGTCAGTTCCGGGTGGTCAA | GGAGCTCCCCACTGTAGCTG |
| **NCAM/CD56** | GGCATTTACAAGTGTGTGGTTAC | TTGGCGCATTCTTGAACATGA |
| **Enolase 2/NSE** | TGTGTGCCTGGGGGAGTTTT | GTGGCAACTGTGGAACGTGG |
| **[Oct4]** | AACCCACACTGCAGCAGATCA | GGGCCAGAGGAAAGGACACT |
| **Sox2** | GGCAGCTACAGCATGATGCAGGAGC | CTGGTCATGGAGTTGTACTGCAGG |
| **Nanog** | CTGCTGAGATGCCTCACACG | TGCCTTTGGGACTGGTGGA |
| **Sox17** | GGTGGACCGCACGGAATTTG | CGGAGTCATGCCCCTGGTAG |
| **TFAP2C** | TGGCCTGGGGGAAGAGTTTG | CTAAGAAGTCCAGGGGCGGG |
| **Blimp1** | CAGCCCTGGGAATACGGTGT | GTGCGTAGCCAGGGTAGGAG |
| **Nanos3** | TCTGCAAACACAACGGCGAG | GTGGCTGTAGACGGAGGTGT |
| **McPYV LT (MCV LT)** | CCACAGCCAGAGCTCTTCCT | TGGTGGTCTCCTCTCTGCTACTG |
| **RnaseP** | AGATTTGGACCTGCGAGCG | GAGCGGCTGTCTCCACAAGT |

**Table S5. Primers for RT-qPCR**

| Genes | Host | Company | Catalog Number | Dilution | Time for steaming |
|---|---|---|---|---|---|
| INSM1 | Rabbit | Milipore | 475R-95 | 1:50. | 40 mins |
| Chromogranin | Rabbit | Cell Signaling Technology | 83651S | 1:100. | 40 mins |
| Synaptophasin | Rabbit | Cell Signaling Technology | 36406S | 1:100. | 40 mins |
| NCAM | Rabbit | Cell Signaling Technology | 99746S | 1:100. | 40 mins |
| Pax6 | Rabbit | Cell Signaling Technology | 60433S | 1:50. | 40 mins |
| ATOH1 | Rabbit | Proteintech | 21215-1-AP | 1:2000. | 40 mins |
| Neurofilament (NF) | Rabbit | Cell Signaling Technology | 2837S | 1:50. | 40 mins |
| NSE | Rabbit | Cell Signaling Technology | 24330S | 1:50. | 40 mins |
| GFAP | Rabbit | Cell Signaling Technology | 80788S | 1:50. | 40 mins |
| | | | | | |
| PanKeratin | Rabbit | Proteintech | 26411-1-AP | 1:1500. | 40 mins |
| KRT20 | Rabbit | Cell Signaling Technology | 13063S | 1:400. | 40 mins |
| EMA | Rabbit | Zeta Corporation | ZR133 | 1:100. | 40 mins |
| | | | | | |
| Oct4. | Rabbit | Cell Signaling Technology | 2750S | 1:100. | 40 mins |
| Nanog | Rabbit | Cell Signaling Technology | 4903S | 1:100. | 40 mins |
| Sox2 | Rabbit | Cell Signaling Technology | 14962S | 1:300. | 40 mins |
| | | | | | |
| SATB2 | Rabbit | Cell Signaling Technology | 39229T | 1:600. | 40 mins |
| | | | | | |
| MCPyV LT | Mouse | Santa Cruz Biotechnologies | sc-136172 | 1:50. | 40 mins |
| | | | | | |
| CD30 | Rabbit | Cell Signaling Technology | 25114T | 1:50. | 40 mins |
| | | | | | |
| PLAP | Rabbit | BioGenex | MU228-5UC | 1:20. | 40 mins |
| 5-Methylcytosine | Rabbit | Invitrogen | MA5-24694 | 1:200,000. | 1 hour |

**Table S6. Primary antibodies used for IHC studies.**
